# Robust Selection Algorithm (RSA) for Multi-Omic Biomarker Discovery; Integration with Functional Network Analysis to Identify miRNA Regulated Pathways in Multiple Cancers

**DOI:** 10.1371/journal.pone.0140072

**Published:** 2015-10-27

**Authors:** Vasudha Sehgal, Elena G. Seviour, Tyler J. Moss, Gordon B. Mills, Robert Azencott, Prahlad T. Ram

**Affiliations:** 1 Department of Systems Biology, The University of Texas MD Anderson Cancer Center, Houston, Texas, United States of America; 2 Department of Mathematics, University of Houston, Houston, Texas, United States of America; Harbin Medical University, CHINA

## Abstract

MicroRNAs (miRNAs) play a crucial role in the maintenance of cellular homeostasis by regulating the expression of their target genes. As such, the dysregulation of miRNA expression has been frequently linked to cancer. With rapidly accumulating molecular data linked to patient outcome, the need for identification of robust multi-omic molecular markers is critical in order to provide clinical impact. While previous bioinformatic tools have been developed to identify potential biomarkers in cancer, these methods do not allow for rapid classification of oncogenes versus tumor suppressors taking into account robust differential expression, cutoffs, p-values and non-normality of the data. Here, we propose a methodology, Robust Selection Algorithm (RSA) that addresses these important problems in big data omics analysis. The robustness of the survival analysis is ensured by identification of optimal cutoff values of omics expression, strengthened by p-value computed through intensive random resampling taking into account any non-normality in the data and integration into multi-omic functional networks. Here we have analyzed pan-cancer miRNA patient data to identify functional pathways involved in cancer progression that are associated with selected miRNA identified by RSA. Our approach demonstrates the way in which existing survival analysis techniques can be integrated with a functional network analysis framework to efficiently identify promising biomarkers and novel therapeutic candidates across diseases.

## Introduction

MicroRNAs (miRNAs) are small non-coding RNA regulators that bind to complementary sequences on target messenger RNAs (mRNAs), resulting in the target mRNAs’ translational suppression or degradation. MiRNAs may also bind to complementary sequences in the promoter region of the target genes and cause transcriptional activation [1, 2]. Thus, changes in miRNA expression affect gene regulation, which in turn leads to changes in cellular homeostatic stability [3, 4].

Several miRNAs have been shown to play an important role in cancer [5–7]; and studies have also shown that more than 50% of miRNA genes are located in cancer-associated genomic regions [8]. Many miRNAs have been shown to play crucial roles as cancer-inducing oncomiRs or as tumor suppressor miRs [9]. For instance, miR-21 is a well-studied oncomiR that is upregulated in many different cancers, [10, 11]. and plays an important role in drug resistance [12]. Members of the miR-17-92 family also function as prominent oncomiRs [13] and can promote cancer development by negatively regulating tumor suppressor genes. On the other hand, miRNAs such as those in the let-7 family function as tumor suppressor miRs [14–16] and can inhibit cancer by inhibiting oncogenes and regulating functions such as apoptosis and cell differentiation.

Several groups have studied the capacity of miRNAs to be used as biomarkers for specific cancers [17–22]. In most of these studies, researchers used sequencing, microarrays or PCR–based techniques for global profiling of miRNAs, and have thereby identified several miRNAs that play important roles in cancer. However, these approaches suffer from multiple limitations. As shown in our paper, current methods for the analysis of miRNA or other omics data that rely on arbitrary choices such as picking thresholds for separating patients into high and low expression groups can be *very sensitive* to small random changes in the patients group, resulting in a high false discovery rate. Thus, we present an innovative robust systems analysis in which miRNAs are coupled to patient survival outcomes across different cancer types to more quickly and efficiently identify potential oncomiRs and tumor suppressor miRs.

A further limitation of current methodologies is the high number of identified miRNAs and the associated difficulty in validating so many miRNAs experimentally. In order to further narrow down the number of miRNAs to those with the highest potential in multiple cancer types, we additionally sought to integrate functional network analysis. The primary function of miRNA is in regulating mRNA levels in the cell by binding to sequences in the 3’ UTR of the mRNA, resulting in a change in the steady state levels of the mRNA and subsequent change in the functional output of the gene [23–25]. Therefore, we sought to identify functional miRNA-mRNA networks based on the correlation of the miRNA and mRNA expression levels in patient tumors in which miRNA showed clinical significance.

With the exponential increase in the amount of data that is generated from patient samples measuring various molecular characteristics at the omics or global level from each patient, the development of complementary bioinformatics and systems biology analysis tools is imperative. We herein propose a workflow that integrates the survival analysis of omics data with functional network analysis techniques to identify potential miRNA biomarkers and the pathways they influence across diverse cancer types. Since our approach takes into account the potential *non-linear* functional relationships between potential markers’ expression levels and patients’ survival outcomes, its performance exceeds that of traditional correlation analysis, which is restricted to discovering approximately *linear* functional relationships. Moreover, we propose non-parametric data analysis techniques for which no implicit normality assumptions regarding the distribution of gene expression levels are required, since the majority of omics data does not follow the normal distribution. In this study, we demonstrated the utility of this approach using patient datasets from The Cancer Genome Atlas (TCGA) to identify prognostic biomarkers and further validated the proposed workflow using a previously published dataset.

## Methods

**Fig 1:**
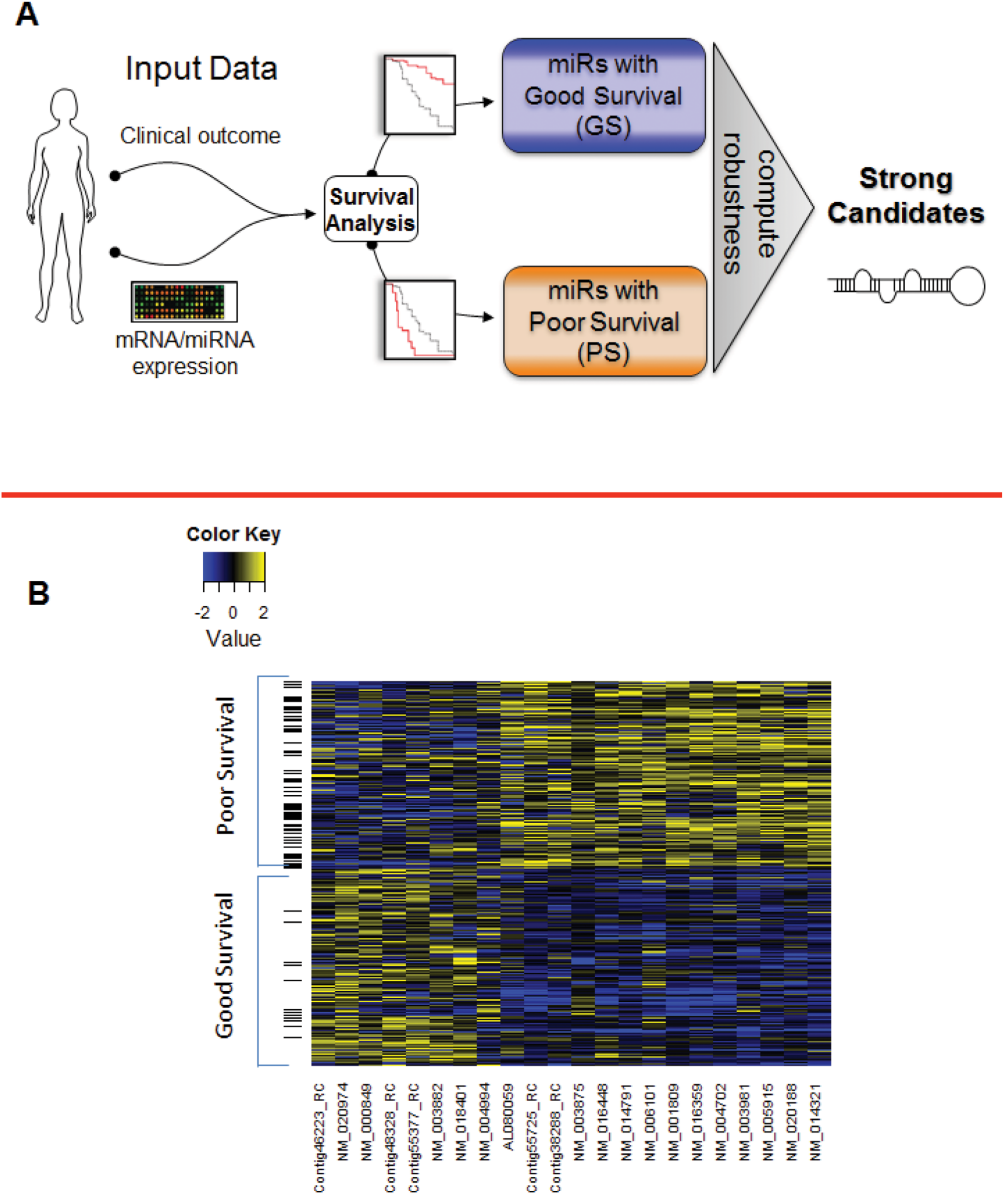
Workflow of our robust selection algorithm (RSA) and validation of the RSA using previously published datasets. (A) Schematic displaying the overview of the RSA. The inputs are clinical data and miRNA expression data; the outcomes are candidate miRNAs correlated with either good or poor survival. (B) Validation of the RSA using previously published gene signatures correlated with survival outcomes. We applied RSA to breast cancer dataset in Martin et al. And looked at the overlap of genes correlated with good and poor survival computed by RSA and from their results. Heatmap of these overlapping genes was drawn displaying the high gene intensity in yellow and low gene intensity in blue.

Because we sought to identify miRNAs that act as either tumor suppressors or as oncomiRs, we classified each miRNA with strong impact in terms of patient survivalas having either high expression linked to good patient survival (GS miRNAs) or high expression linked to poor patient survival (PS miRNAs). We reviewed patient data for clinical outcomes and miRNA expression levels; we have developed a new Robust Selection Algorithm (RSA), which we used to classify miRNAs as being associated with either good or poor survival. We introduced and computed an innovative *robust p-value* to quantify the impact of each candidate miRNA on good or poor survival (Fig 1A and Figure A and Figure B in S1 File). To demonstrate the proposed workflow, we applied our RSA and the subsequent functional pathway analysis to TCGA datasets for five cancer types: breast, ovarian, head and neck, lung, and kidney (information useful for downloading this data is found in S1 Table).

### Data and Pretreatment

TCGA contains various forms of omics data including miRNA expression, mRNA expression. It also contains clinical data from these patients giving information about the survival of these patients. Using different cancer patients’ RNA sequence data from TCGA, we extracted each miRNA’s average mature and star strand expression separately. TCGA has data available in miRNAseq form, and we were able to search 2092 miRNAs (the total miRNAs for which data is available) to identify candidate miRNAs whose differential expression correlated with survival.

TCGA miRNA expression data are acquired using either the Illumina Hiseq or Illumina GA platform. Running our initial analyses on these two platforms separately yielded disparate results. We then investigated the two platforms’ miRNA expression distributions to determine whether we could combine the two platforms’ samples to obtain a larger number of patient samples. To compare the two platforms’ miRNA distributions, we applied the Kolmogorov-Smirnov test using the null hypothesis that the two distributions are the same at 5% significance. This helped us identify which miRNAs had similar (though respectively distinct) distributions in both platforms.

We also downloaded clinical data for each of the 5 cancer types mentioned above from TCGA. From this data, we extracted patients’ survival times until death or censoring. Several patient data in TCGA were annotated as having no follow-up time and thus were systematically removed from our final dataset analysis. We then matched the patients for whom clinical and RNA sequence data were available.

### Homogenizing Data Across Platforms

TCGA miRNA expression data for different cancer types were generally acquired using different platforms. To normalize miRNA expression levels and correct for artefacts due to data generation using different acquisition modalities, we pooled all the available TCGA miRNA expression data and subjected it to a homogenization step as explained further in this section. We then used these normalized values for our final dataset analysis. This homogenization step is important as it corrects for data artefacts due to data generation through different platforms and acquisition modalities.

The two platforms’ miRNA distributions were not very similar and thus could not be combined using a standard median normalization step. Therefore, we performed the following homogenization procedure to combine the platforms’ miRNA expression distributions for each cancer type. To obtain an identical cumulative distribution function (CDF) of the homogenized expression values obtained with both platforms, we homogenized the two miRNA expression distributions derived from the two platforms. The “target” CDF is defined as the average CDF of the two platforms, namely, *F(x) = 0*.*5F1(x) + 0*.*5F2(x)*, where F1 and F2 are the cdf’s of the two platforms respectively. Let *G* be the inverse function of *F*. Each expression value *x* from platform 1 is matched to a homogenized expression value, *z(x)*, which is calculated by inverting the function *F* at the value *F1(x)*; thus, *z(x) = G(F1(x))*. Each expression value from platform 2 is homogenized similarly, with *z(y) = G(F2(y))*.

For any value, 0≤ K ≤ 1, {F(z(x)) ≤ K} iff {z(x) ≤ G(K)} iff {G(F1(x)) ≤ G(K)} iff {F1(x) ≤ K}, and similarly, {F(z(y)) ≤ K} iff {z(y) ≤ G(K)} iff {G(F2(y)) ≤ G(K)} iff {F2(y) ≤ K}.

Thus, we match the quantiles *x* and *y* in the separate distributions with their quantiles *z(x)* and *z(y)* in the combined distribution *F*.

### Robust Selection Algorithm

A literature search was performed to identify a methodology that could be used to improve existing methods of evaluating miRNAs and identifying the cancer-related pathways they influence. We identified one study that evaluated the prognostic values of specific miRNAs in several cancer types [26]; however, we have checked that the methodology of [26] is potentially quite sensitive to even small perturbations of the existing patients group, and we have validated this instability by applying it to our data.

To test the sensitivity of the methodology to patient group, we used the kidney cancer dataset downloaded from TCGA. From this dataset, we created 100 simulated datasets by randomly dropping 2% patients in each simulated dataset. On each simulated dataset, we then used the methodology of [26] to select miRs strongly correlated with patient survival. In this way, we obtained 100 lists of selected miRNA. We then enumerated all those miRNA which appeared in 99 or more of these 100 lists. The stability of the methodology was then characterized by looking at the histogram of the fraction of the selected miRNA which were stable. Since 2% variation in the patient groups is a small variation, we should require a robust methodology to select similar miRNA repeatedly. However, our simulations suggest that the methodology in [26] only selects 68% stable miRNA, with the rest being sensitive to the specific composition of the patient group (see S30 Fig for a quantification of how small changes in the data can lead to a large reduction in the stability of identified biomarkers).

Further, this and other such studies, often use a single threshold of expression data to compare the survival curves, and gives results for candidate miRNAs for a cancer type at a time. Therefore, we developed a robust selection algorithm (RSA) that uses a non-parametric statistical joint analysis of patient survival data and patient-specific miRNA expression levels to quantify the prognostic value of each miRNA. In contrast to methods that use a single threshold to compare survival data, our RSA eliminates the use of single threshold for Kaplan-Meier survival curve analysis, by choosing from a wide array of cutoffs from expression data using a range of statistically relevant cutoff values. Thus, the performance of our RSA is quite resistant to small random perturbations of the patients group.

Clinically, miRNAs whose expressions are associated with different actions are afforded different treatment. For instance, a miRNA whose high expression is correlated with longer survival (i.e., tumor suppressors) is treated differently from one whose high expression is correlated with shorter survival (i.e., oncomiRs). Therefore, we first classify each miRNA as a GS miRNA (high expression–good survival) or a PS miRNA (high expression–poor survival). This initial classification step is performed by first computing the median survival time of all available patients, from the Kaplan–Meier survival estimates and then classifying miRNAs as follows.

Using TCGA data, we first compute the Kaplan-Meier estimates of the censored survival time for the patients in which a miRNA is expressed. We then use the expression histogram data to identify two groups of patients: patients with high miRNA expression and patients with low miRNA expression. For each miRNA, *m*_*j*_, we separate patients into high miRNA expression or low miRNA expression groups using a finite grid of cut-offs,*C*, that range from the 45% quantile to the 60% quantile of the distribution of the expression levels in increments of 1%. At each such cut-off *C* we define

*G*_*high*_ = group of patients with high miRNA expression = group in which miRNA expression is larger than the (*C*+4) quantile of the expression levels distribution
*G*_*low*_ = group of patients with low miRNA expression = group in which miRNA expression is less than the *C* quantile of the expression levels distribution

The high miRNA expression and low miRNA expression groups are separated by a "neutral" group in which miRNA expression levels are between *C*% and (*C*+ 4)%. This 4% margin can be increased without impairing the analysis as long as the high miRNA expression and low miRNA expression groups are reasonably large.

For each cutoff C%, we separately compute the Kaplan-Meier estimates of the survival curves for the *G*_*high*_ and *G*_*low*_ groups. The log-rank test is used to assess the difference between the two Kaplan-Meier survival curves, and a p-value, *pval(C)*, is computed. The null hypothesis for the log rank test is that the two survival curves are the same. The optimal cut-off *C%* for separating patients into *G*_*high*_ or *G*_*low*_ is chosen to minimize *pval(C)*. Let *q*_*j*_ be the optimal chosen cut-off for each miRNA *m*_*j*_. For each miRNA *m*_*j*_, we compute the median survival times for patients in the high miRNA expression group (*Med*_*high*_) and for patients in the low miRNA expression group (*Med*_*low*_) at the optimal cut-off_*qj*_. We then classify the miRNA into the following two groups:

$$GS\text{if}{Med}_{low}\leq {Med}_{all}\leq {Med}_{high}$$

$$PSif{Med}_{high}\leq {Med}_{all}\leq {Med}_{low}$$

Examples of this type of miRNA characterization are shown in Figure B of S1 File. For each miRNA m_j_ belonging to the GS or PS groups, the preceding computation also give us *j = pval(q*_*j*_ *)*, which quantifies the significance of the potential link between miRNA *m*_*j*_ and patient survival time. Kaplan-Meier survival plots for patients with the five significant candidate miRNAs of interest across different cancer types along with the overall survival curve for patients with that cancer type are shown in S27 and S28 Figs.

### Generation of Robust p-Values

We have repeatedly noted that the p-values computed with the preceding method can be somewhat sensitive to the specific patients group. To eliminate this sensitivity, we introduce and apply an innovative resampling procedure to generate *robust p-values*. The method described in the preceding section is used to determine whether miRNA expression has a potential non-linear significant correlation with survival. For each GS miRNA or PS miRNA, we introduce a random resampling technique to compute a robust p-value, *PV(M*_*j*_ *)*, to replace the preceding p-value, *pv(m*_*j*_ *)*. To implement this resampling, for each cut-off *C%* and each fixed miRNA *m*_*j*_, we randomly drop 1% of patients from each of the two groups *G*_*high*_ and *G*_*low*_. and we compute the Kaplan-Meier survival curves for these two perturbed patients groups.

As above, we first compute the optimal cut-off that best separates the miRNA expression distribution based on the perturbed Kaplan-Meier survival plots and then compute the p-value *pv(m)* or survival at this optimal cut-off. For each fixed miRNA *m*_*j*_, repeating the randomized perturbation process 500 times generates a set of 500 virtual p-values *pv(m)*. To define a reliable upper-limit *PV(m*_*j*_ *)* for the unknown p-value *pvl(m*_*j*_ *)*, we set *PV(m*_*j*_ *)* to be equal to the 75^th^ percentile of the 500 virtual p-values. We call *PV(p*_*j*_ *)* the *robust p-value* for miRNA *m*_*j*_. The miRNAs *m*_*j*_ with significant robust p-values *PV(m*_*j*_ *)* are then classified as candidate miRNAs that are correlated with good or poor survival, thereby providing a list of miRNAs whose differential expression is correlated with either good or poor survival times. The schematic of the algorithm is shown in S29 Fig.

For our analyses, we discard all miRNAs that have an average 0 expression over the patient group. In addition, TCGA samples annotated as having no follow up time were not included in our analysis.

### Cancer Types

To identify candidate miRNAs whose differential expression is strongly linked with more than one cancer type, we applied our RSA to multiple cancer patient datasets available in TCGA. We applied our RSA to the datasets of cancer types represented by at least 400 samples and for which matched clinical and miRNA expression data were available, namely, breast (BRCA), ovarian (OVCA), head and neck (HNSC), lung (LUAD), and kidney (KIRC) cancer. The numbers of matched samples for each of these cancer types are shown in S1 Fig. Because breast cancer is a subtype-specific disease, we also investigated breast cancer subtypes individually to determine whether a specific subtype was responsible for the strong link between differential miRNA expression and patient survival.

### Validation

Martin *et al*. [27, 28] pooled matched survival and gene expression data from six different breast cancer patient datasets and found that pooling the data synergistically affected classification performance and improved gene signature stability. The authors used the pooled dataset to identify a gene expression signature correlated with patient survival. Because our RSA can be used to analyze not only miRNA expression data but also gene or protein expression data, we selected this dataset for validation. We used this dataset (accessible through the Gene Expression Omnibus) to validate the performance of our RSA in identifying mRNA correlated with patient survival. We applied our RSA to the pooled dataset from Martin et al. to identify genes whose differential expressions were correlated with patient survival. In their paper, they identified clusters of genes strongly correlated with good and poor survival. Application of our method RSA to their dataset also identified 1 cluster of genes whose high expression was strongly linked with good survival and another cluster of genes whose high expression was linked to poor survival. Moreover the two methods gave an overlap of 22 genes. A heatmap of the common genes indicating their correlation with survival is displayed in Fig 1B.

### Integrating Joint miRNA-mRNA Expression Levels to Generate Functional Networks

To identify the pathways regulated by each candidate miRNA our RSA selected, we gathered patient-specific joint miRNA-mRNA expression data from TCGA and analyzed them to generate miRNA-mRNA correlation networks. Correlations were computed using a multivariate linear model that accounts for mRNA expression level variations induced by DNA copy number alterations and promoter methylation at the gene locus. We computed ranked lists of genes and corresponding regression coefficients as described previously [29]. To reduce potential misrepresentation of the data due to stromal contamination in the samples, we removed genes associated with the extracellular matrix (S8 Fig). Instead of focusing on individual genes that are strongly correlated with a given candidate miRNA, we used NetWalker [30], a software suite that integrates gene expression data and molecular interaction data to score known interactions, to identify whole interaction networks that were positively or negatively correlated with the candidate miRNA. Using the miRNA-mRNA regression coefficients as input values for NetWalker, we calculated edge flux values for the known molecular interactions, and we used the interactions with the highest edge flux values (top 200 positive and top 200 negative interactions) to generate the networks. The Log2 of the beta values is displayed for all the networks.

We constructed miRNA-mRNA interaction networks for the five most robust candidate miRNAs that were significantly correlated with survival outcomes in four cancer types (i.e., LUAD, HNSC, KIRC, and OVCA). These five candidate miRNAs’ networks, which include genes that are either positively (yellow) or negatively (blue) correlated with high miRNA expression, are shown in S9–S29 Figs. To identify pathways potentially regulated by these five candidate miRNAs across diverse cancer types, we first identified the cancer types in which these miRNAs were associated with the same prognosis (i.e., either good or poor survival) and then analyzed the common gene ontology terms associated with the networks for these cancer types.

## Results

We applied our RSA to TCGA patient data that include miRNA expression levels and clinical outcomes. After pre-treating the data, which included the homogenization procedure, to remove effects of different platforms for extraction of miRNA expression, we first computed an optimal threshold that would best separate the miRNA expression levels in terms of survival outcomes computed using the Kaplan-Meier method and the log-rank test. We then clustered the miRNAs into groups, miRNAs associated with good survival (GS miRNAs) and miRNAs associated with poor survival (PS miRNAs), by comparing the median overall survival in optimal groups with the median overall survival of the whole population. Using intensive random sampling, we computed a robust p-value for each candidate miRNA to identify candidate GS miRNAs or PS miRNAs for each cancer type.

Next, we characterized the identified candidate miRNAs by chromosome location and genomic stability and constructed miRNA-mRNA functional networks. By analyzing the interactions between prognostic miRNA markers and functional pathways involved in cancer progression, we determined the main pathways these miRNA prognostic markers affect.

### miRNA–Disease Survival Network

**Fig 2:**
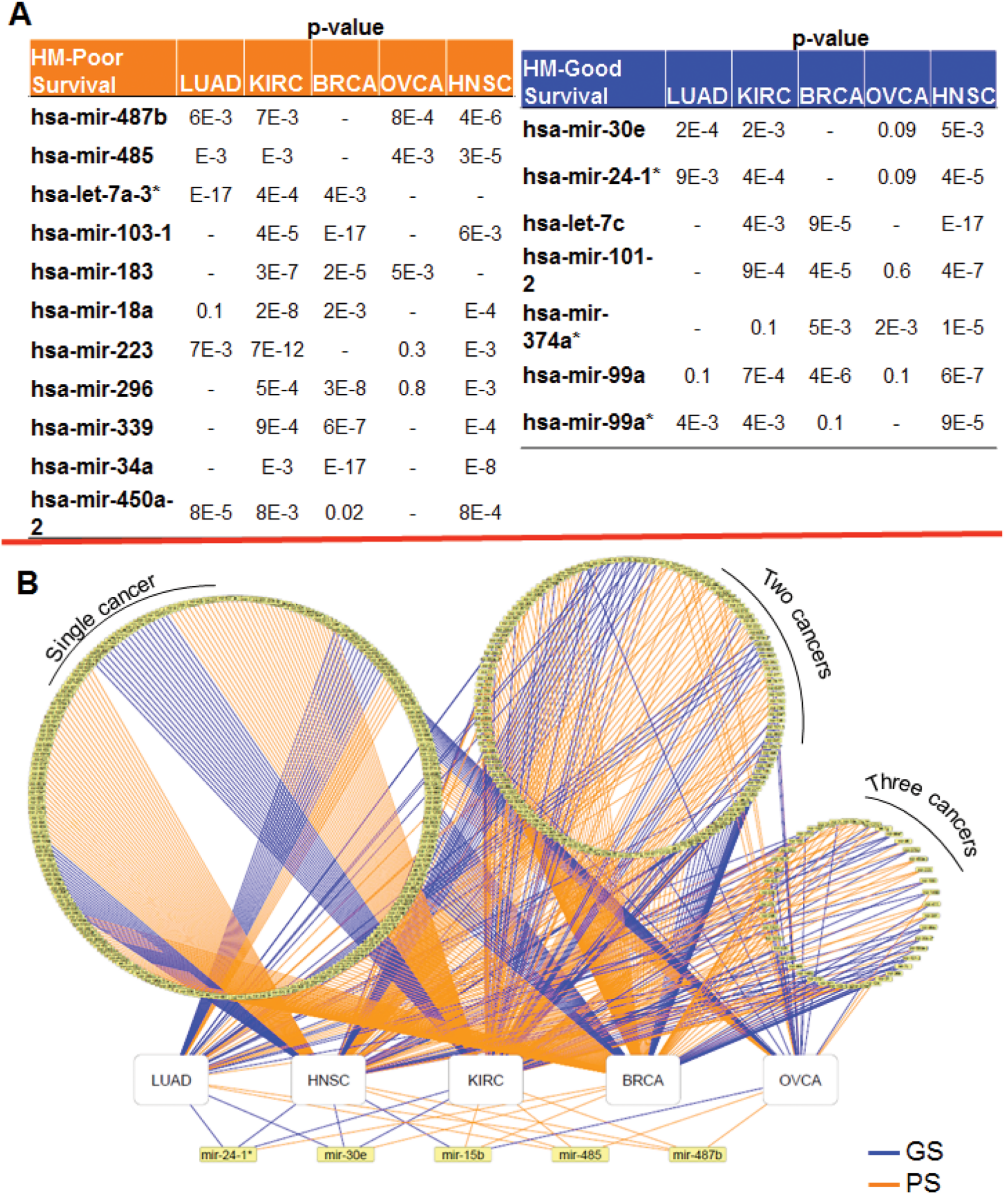
Candidate miRNAs significantly correlated with survival across cancer types. (A) Candidate miRNAs from RSA significantly (robust p-value < 0.01) correlated with good survival or poor survival in at least 3 cancer types. (B) MiRNA-disease survival network. The circles indicate the miRNAs strongly linked with patient survival across diverse cancer types. Left to right: miRNAs linked to prognosis in one cancer type, 2 cancer types, and 3 cancer types. White rectangles represent cancer types. Yellow rectangles represent miRNAs. The color of the edge between a miRNA and a cancer type, indicates whether the miRNA is correlated with good (blue) or poor (orange) prognosis in a cancer type.

For each cancer type, namely, breast (BRCA), ovarian (OVCA), head and neck (HNSC), lung (LUAD), and kidney (KIRC) cancer, we identified candidate miRNAs whose differential expression was strongly linked with patient survival in multiple cancer types. The GS miRNA and PS miRNA candidates for which a significant robust p-value indicated a correlation with survival in at least 3 different cancer types are shown in Fig 2A. We defined and constructed miRNA–disease survival networks which encoded associations between miRNA and cancer types (Fig 2B). Different circles contain miRNAs linked with prognosis in (from left to right) one, two, or three cancer types. Below these 3 circles, the miRNAs significantly linked with prognosis in four cancer types are indicated. Since our first priority was to identify targets that are valid in multiple cancer types, we selected five miRNAs (miR-24-1*, miR-30e, miR-15b, miR-485, and miR-487b) that were strongly linked with survival (robust p-value ≤ 0.01) in multiple cancer types.

### Copy Number Alterations

**Fig 3:**
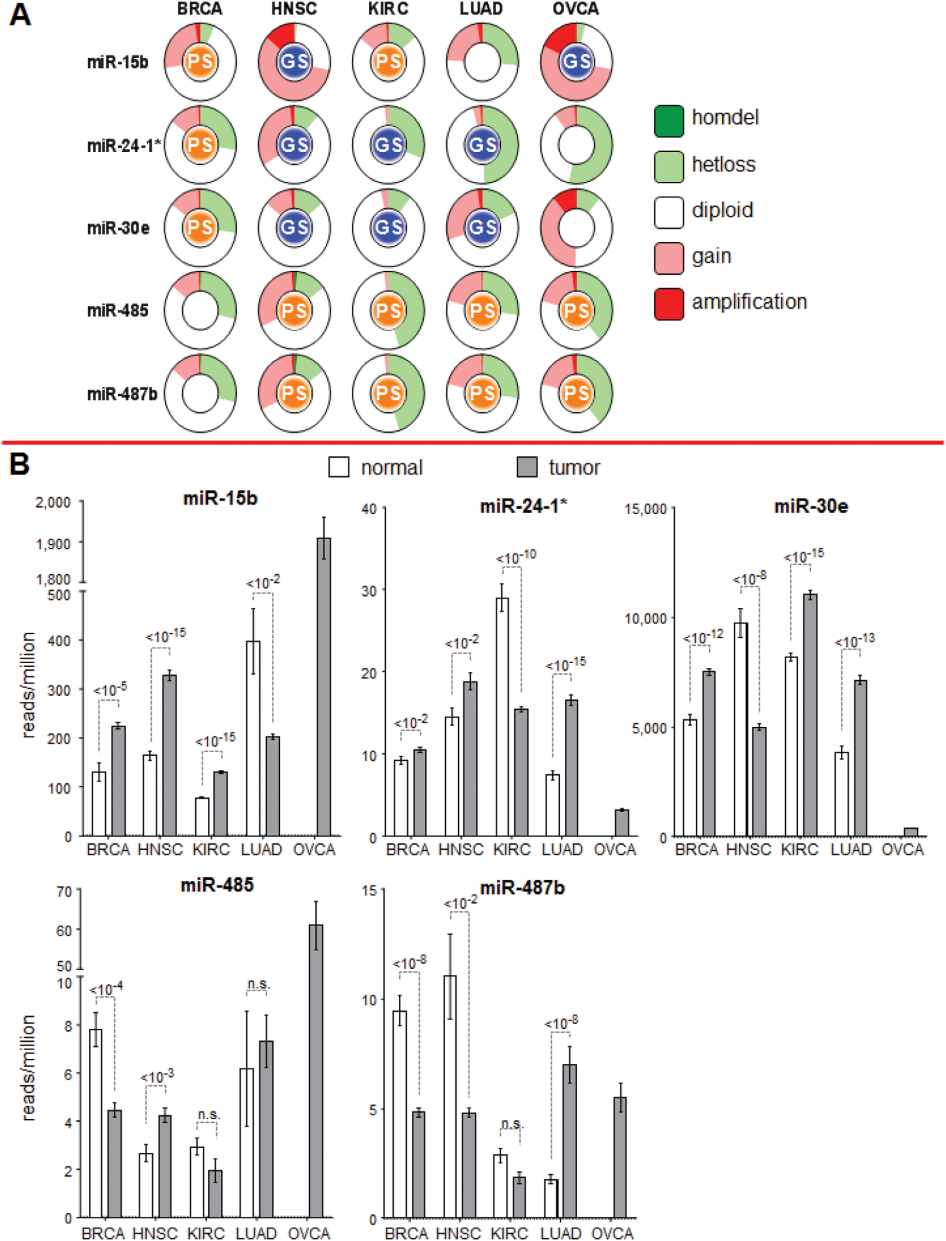
Characterization of miRNAs found to be strong candidate markers of prognosis based on copy number variation and expression. (A) Further characterization of the 5 strong candidate miRNAs in terms of copy number variation and expression. The GISTIC-identified copy number alterations at each of the chromosome loci for the miRNAs in different cancer types are displayed. The “GS” or “PS” inside each circle indicates the link with good (blue) or poor (orange) prognosis. (B) Expression in tumor and normal tissue for each of the strong candidate miRNA. For OVCA, the normal tissue data were not available.

Each candidate miRNA strongly linked with patient survival in at least 4 different cancer types was further investigated in terms of its chromosome location and expression pattern in patients. The GISTIC scores in copy number alterations for each of the chromosome locations of these miRNAs in each cancer type were obtained from the cBio data portal and are shown in Fig 3A. miR-485 and miR-487b, which are located very close to each other on chromosome 14, have similar relationships with prognosis in diverse cancer types and have similar copy number alterations across these cancer types (Fig 3A). miR-15b is strongly linked with good survival in HNSC and OVCA and displays similar copy number gains in these cancers. A gain in copy number at a given chromosome location would indicate increased expression of the relevant miRNA. For each selected miRNA, the patterns of its expression levels in normal and tumor tissues are similar to the corresponding profiles of copy number alterations (Fig 3B). (We could not make a similar comparison in OVCA, as we did not have data for normal tissue samples.)

We also computed the correlation between the copy number alterations at the chromosome location of each candidate miRNA and the changes in methylation levels for each cancer type individually and for all 5 cancer types combined (S2–S6 Figs). We found significant correlation between miRNA expression and copy number variation at those loci and between miRNA expression and methylation levels in the relevant cancer types. When we analyzed the pooled data from the 5 cancer types, we still observed significant correlations between miRNA expression and copy number variation and methylation levels. We could not perform a similar analysis on the ovarian cancer dataset because no methylation data were available for ovarian cancer patients in TCGA.

### Breast Cancer Subtypes

Given the heterogeneity of breast cancer, we also applied our RSA to data from each of 4 breast cancer subtypes (luminal A, luminal B, basal, or Her2-enriched based on the PAM50 panel). The RSA identified miR-15b, miR-24-1*, and miR-30e as being strongly linked with poor survival for these breast cancer subtypes, particularly the luminal A subtype (S7 Fig). The expression levels of these miRNAs in the basal subtype were higher than those in the luminal A, luminal B, and Her2-enriched subtypes.

### Integrating Functional Networks

**Fig 4:**
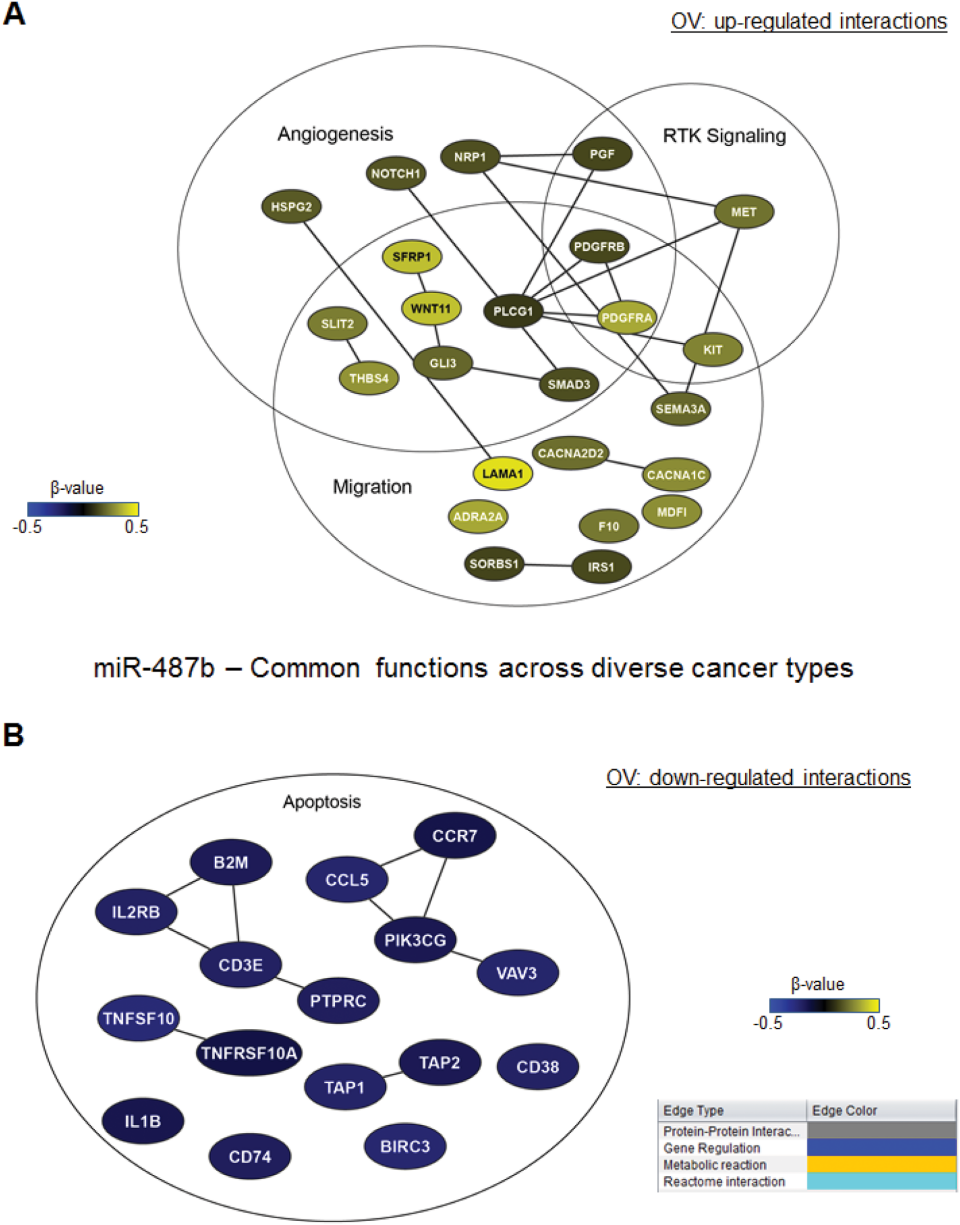
miRNA-mRNA interaction networks for miR-487b, whose functions are conserved across cancer types. miR-487b miRNA-mRNA interaction networks. mRNA networks that were positively (yellow) or inversely (blue) correlated with miR-487b in OVCA and involved in functions conserved across cancer types are shown.

We found that miR-487b is strongly linked with poor survival across the 4 cancer types. The regulatory functions of miR-487b that are preserved across these 4 cancer types and the genes that are positively (yellow) or inversely (blue) correlated with this miRNA in these cancers are shown in Fig 4A and 4B. The genes involved in angiogenesis and in receptor tyrosine kinase signaling were positively correlated with miR-487b, whereas the genes involved in apoptosis were negatively correlated with miR-487b.

**Fig 5:**
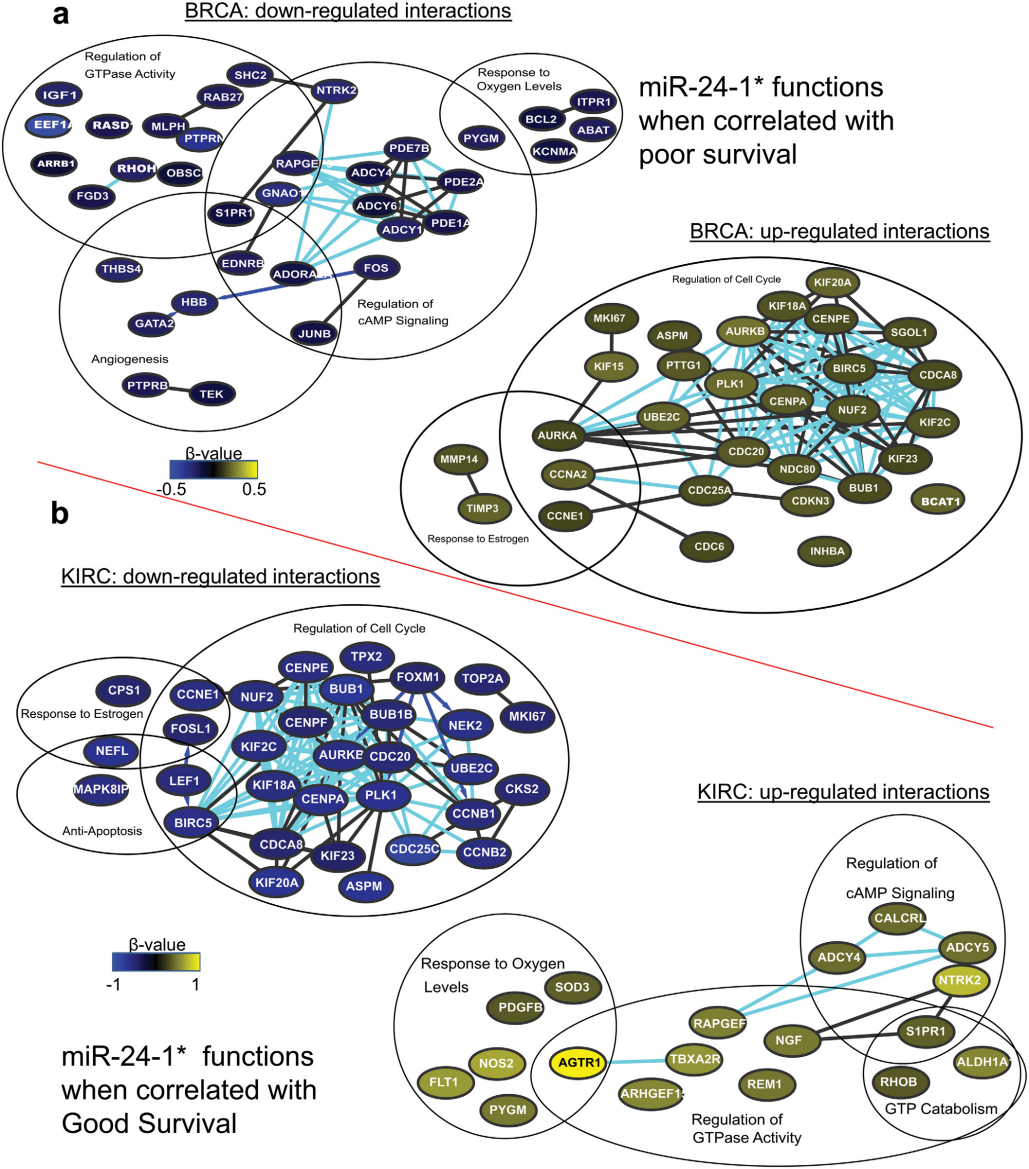
miRNA-mRNA interaction networks for miR-24-1*, whose functions are conserved across cancer types. (A) miR-24-1* miRNA-mRNA interaction networks. Networks of positively (yellow) and inversely (blue) correlated mRNA and associated functions in BRCA, in which miR-24-1* is correlated with poor survival. (B) Common functions associated with the miRNA-mRNA correlation networks when miR-24-1* is correlated with good survival in three different cancer types. The log of the beta values in KIRC is displayed.

We found miR-24-1-* to be linked with poor survival in BRCA and with good survival in HNSC, KIRC, and LUAD. In BRCA, genes involved in cell cycle regulation were positively correlated with miR-24-1*, whereas genes involved in the regulation of cAMP signaling and GTPase activity were negatively correlated with miR-24-1* (Fig 5A). In contrast, in HNSC, KIRC, and LUAD, the genes involved in cell cycle regulation were inversely correlated with miR-24-1*, whereas the genes involved in the regulation of cAMP signaling and GTPase activity were positively correlated with miR-24-1* (Fig 5B), which suggests that these functions are also positively correlated with good survival.

**Fig 6:**
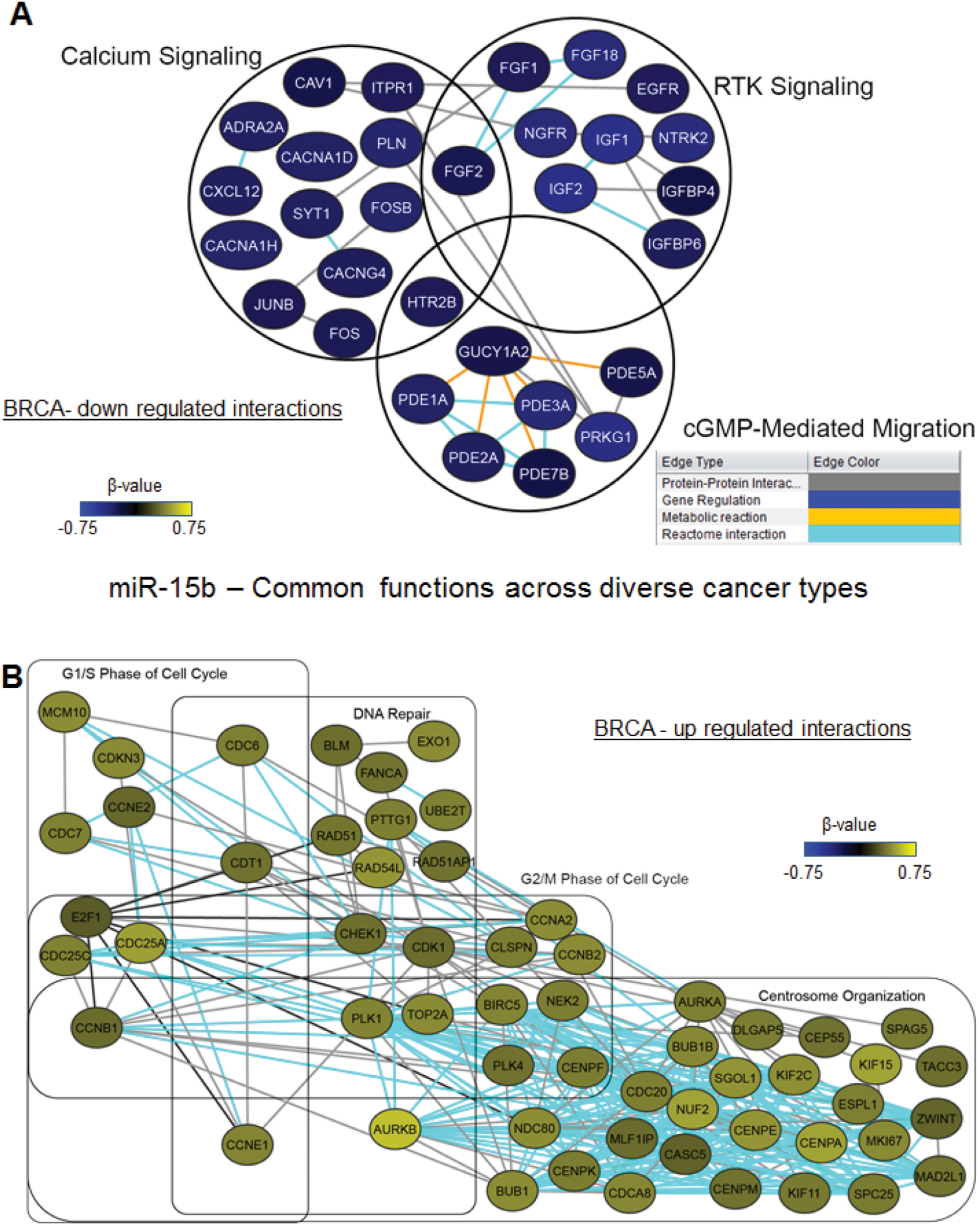
miRNA-mRNA interaction networks for miR-15b, whose functions are conserved across cancer types. (A) Inversely correlated miRNA-mRNA network in BRCA showing conserved functions across 4 cancer types. (B) Positively correlated miRNA-mRNA network in BRCA showing conserved functions across 4 cancer types.

Finally, we found miR-15b to be correlated with good survival in HNSC and OVCA but correlated with poor survival in KIRC and BRCA. The pathways associated with high miR-15b expression in these 4 cancer types are shown in Fig 6. Genes involved in different phases of cell cycle regulation and genes involved in DNA repair and centrosome organization were positively correlated with miR-15b in all 4 cancer types. Moreover, receptor tyrosine kinase signaling and calcium signaling were inversely correlated with miR-15b in all cancer types (Fig 6A).

## Discussion

Our approach identifies biomarkers that are strongly and robustly associated with patient survival. Herein, we describe an approach to the quantitative evaluation of molecular markers’ impact on specific patient outcomes that take into account the potential *non-linear* functional relationships between miRNA expression levels and patient outcomes. Our approach goes well beyond traditional correlation analysis, which is restricted to discover approximately *linear* functional relationships. Moreover, because our approach is non-parametric, one need not make an implicit Gaussian assumption about the distributions of genes’ expression levels.

The introduction of *robust p-values*, computed by intensive simulations of randomly perturbed survival data, is an innovative feature of this approach. By intensive simulations of small random perturbations of our patients groups and efficient pooling of these virtual data analyses, we generate *robust p-values* which are strongly resistant to actual perturbation of the data by noise in the expression levels or by variations in patient samples. Our methods are in fact applicable to any molecularly measured expression data (for instance, mRNA, miRNA, protein expression) and to any measured patient outcome data, including survival and disease progression data.

In contrast to previously published methods [26] for comparing survival times between two miRNA expression groups, in which a single threshold is used to compare significantly different groups, our method eliminates this choice by sampling data over a range of statistically relevant cut-off values and identifying the best cut-off for significantly comparing two sets of survival data groups. Moreover our robust p-values computation strengthens the identification of good miRNA candidates, primarily because it automatically enforces increased consistency with respect to small changes in the patients group. To further strengthen these analyses, we are currently preparing a companion paper analyzing in depth several statistical variants of this new method through theory combined with very intensive simulations, in order to determine the confidence level and the detection capacity of this robust p-value technique.

After identifying clinically relevant miRNA targets across multiple cancer types, we also further characterize these miRNA targets in terms of copy number variation, expression and methylation. The identification of correlated functional networks that may play a role in these processes is also very important to our understanding of complex disease processes such as cancer. Here we have analyzed genes expression levels data in patient tumors to determine functional miRNA-mRNA regulation networks that may impact cell proliferation and/or patient survival. These sub-networks may either be of therapeutic value or could serve as important functional multi-omic biomarkers.

Overall, our results demonstrate that enforcing robustness when using standard statistical techniques and extending the bioinformatics framework by incorporating functional network and pathway analyses more quickly and efficiently identifies potential miRNA biomarkers for the development of anticancer therapies. In addition RSA allows for the automated determination of optimal cutoffs taking into account the non-normality of the data and data obtained across different platforms and sources. The miRNA biomarkers our RSA selects and these markers’ effects on specific functional pathways make them promising candidates for the development of therapeutic strategies for diverse cancer types. A user friendly web based GUI of RSA is currently being developed enabling a pipeline for rapid analysis of multi-omics patient outcome data. Experimental testing of these biomarkers in an independent patient cohort from MD Anderson will be performed in the near future. In addition, experiments to determine the molecular mechanisms of the identified biomarkers and their functional regulation are future avenues of study.

## Supporting Information

S1 Fig The cancer types and numbers of patient samples for which miRNA expression and survival information were available from TCGA. (PDF)

S2 Fig Plots showing correlation between miR-15b expression and copy number alterations and methylation at this chromosome location in different cancer types and across all cancers. (PDF)

S3 Fig Plots showing correlation between miR-487b expression and copy number alterations and methylation at this chromosome location in different cancer types and across all cancers. (PDF)

S4 Fig Plots showing correlation between miR-485 expression and copy number alterations and methylation at this chromosome location in different cancer types and across all cancers. (PDF)

S5 Fig Plots showing correlation between miR-24-1* expression and copy number alterations and methylation at this chromosome location in different cancer types and across all cancers. (PDF)

S6 Fig Plots showing correlation between miR-30e expression and copy number alterations and methylation at this chromosome location in different cancer types and across all cancers. (PDF)

S7 Fig Breast cancer subtype–specific expression of miRNAs correlated with survival in BRCA. (PDF)

S8 Fig Extracellular matrix–associated genes that were discarded from the network analysis. (PDF)

S9 Fig Networks downregulated in the presence of high miR-487b expression in different cancer types (PDF)

S10 Fig Networks upregulated in the presence of high miR-487b expression in different cancer types. (PDF)

S11 Fig Networks upregulated in the presence of high miR-487b expression in different cancer types. (PDF)

S12 Fig Networks downregulated in the presence of high miR-15b expression in different cancer types. (PDF)

S13 Fig Networks downregulated in the presence of high miR-15b expression in different cancer types. (PDF)

S14 Fig Networks upregulated in the presence of high miR-15b expression in different cancer types. (PDF)

S15 Fig Networks upregulated in the presence of high miR-15b expression in different cancer types. (PDF)

S16 Fig Networks downregulated in the presence of high miR-24-1* expression in different cancer types. (PDF)

S17 Fig Networks downregulated in the presence of high miR-24-1* expression in different cancer types. (PDF)

S18 Fig Networks upregulated in the presence of high miR-24-1* expression in different cancer types. (PDF)

S19 Fig Networks upregulated in the presence of high miR-24-1* expression in different cancer types. (PDF)

S20 Fig Networks downregulated in the presence of high miR-485 expression in different cancer types. (PDF)

S21 Fig Networks downregulated in the presence of high miR-485 expression in different cancer types. (PDF)

S22 Fig Networks upregulated in the presence of high miR-485 expression in different cancer types. (PDF)

S23 Fig Networks upregulated in the presence of high miR-485 expression in different cancer types. (PDF)

S24 Fig Networks down or upregulated in the presence of high miR-30e expression in breast cancer, in which high miR30e expression was correlated with poor survival. (PDF)

S25 Fig Networks downregulated in the presence of high miR-30e expression in different cancer types. (PDF)

S26 Fig Networks upregulated in the presence of high miR-30e expression in different cancer types. (PDF)

S27 Fig The Kaplan-Meier survival curves for patients with each of the strong candidate miRNA in each cancer type are displayed. The curves for patients in the high–or low–miRNA expression groups, along with the overall survival curve for that population, are displayed.
(PDF)

S28 Fig The Kaplan-Meier survival curves for patients with each of the strong candidate miRNA in each cancer type are displayed. The curves for patients in the high–or low–miRNA expression groups, along with the overall survival curve for that population, are displayed.
(PDF)

S29 Fig Schematic of the computation involving robust p-value calculation. Some plots for the distribution of p-values are also displayed.
(PDF)

S30 Fig We used random resampling techniques to evaluate the stability of previously existing methodologies. Starting with a kidney cancer dataset from TCGA, we created 100 simulated datasets by dropping 2% patients from the original dataset. On each simulated dataset, we then used the methodology of Reference [26] and create a list of miRNA with p-value smaller than 0.01. In this way we obtain 100 lists. We then enumerate miRNA which occur in 99 or more of these 100 lists; we will refer to this list of miRNA as stable miRNA. The displayed PDF is obtained by computing what fractions of the miRNA selected on each simulated dataset are stable.
(PDF)

S1 File Schematic of the methodology. (Figure A) Schematic of our methodology, which involved computing Kaplan-Meier estimates and performing log-rank tests at different miRNA expression cut-offs. (Figure B) Schematic of our RSA.
(PDF)

S1 Table TCGA data was downloaded using the website https://tcga-data.nci.nih.gov/tcgafiles/ftp_auth/distro_ftpusers/anonymous/tumor/. Level 3 data was used for miRNA expression. For each cancer type, data can be found on the at the link using the platform type and last modified date mentioned in the table.
(PDF)
