# Supplementary material for: Robust Selection Algorithm (RSA) for Multi-Omic Biomarker Discovery; Integration with Functional Network Analysis to Identify miRNA Regulated Pathways in Multiple Cancers
Source: PLoS One. 2015 Oct 27;10(10):e0140072. doi: 10.1371/journal.pone.0140072 (PMC4623517; doi:10.1371/journal.pone.0140072)
Supplement: S1 Fig — (PDF) [file pone.0140072.s001.pdf]

Cancer Type	Number of Matched Samples
Ovarian (OVCA)	474
Lung (LUAD)	429
Kidney (KIRC)	451
Head and Neck (HNSC)	354
Breast (BRCA)	792

S1 Fig
