# Supplementary figures and images for: Robust Selection Algorithm (RSA) for Multi-Omic Biomarker Discovery; Integration with Functional Network Analysis to Identify miRNA Regulated Pathways in Multiple Cancers
Source: PLoS One. 2015 Oct 27;10(10):e0140072. doi: 10.1371/journal.pone.0140072 (PMC4623517; doi:10.1371/journal.pone.0140072)

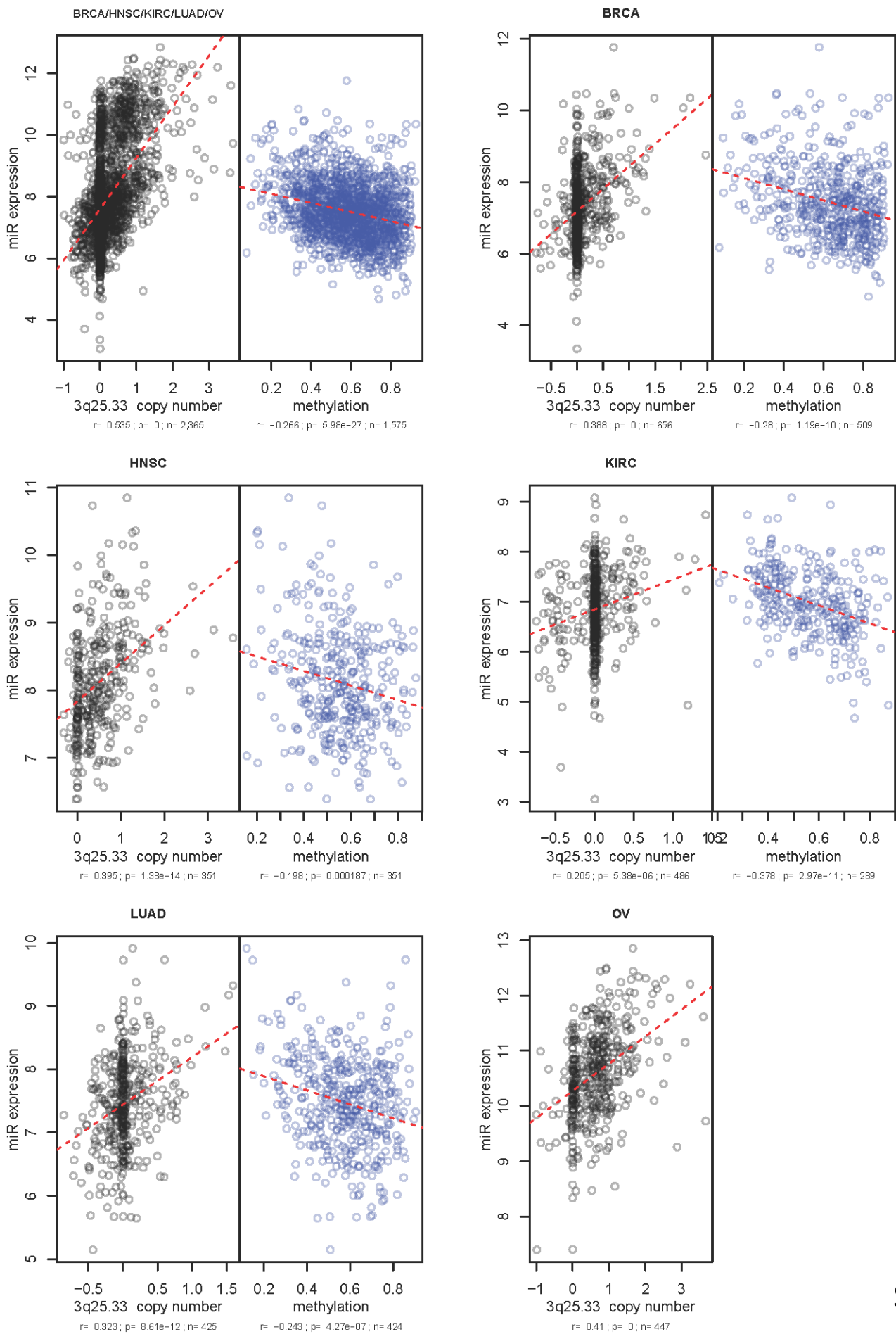

Supplement: S2 Fig — (PDF) [file pone.0140072.s002.pdf]

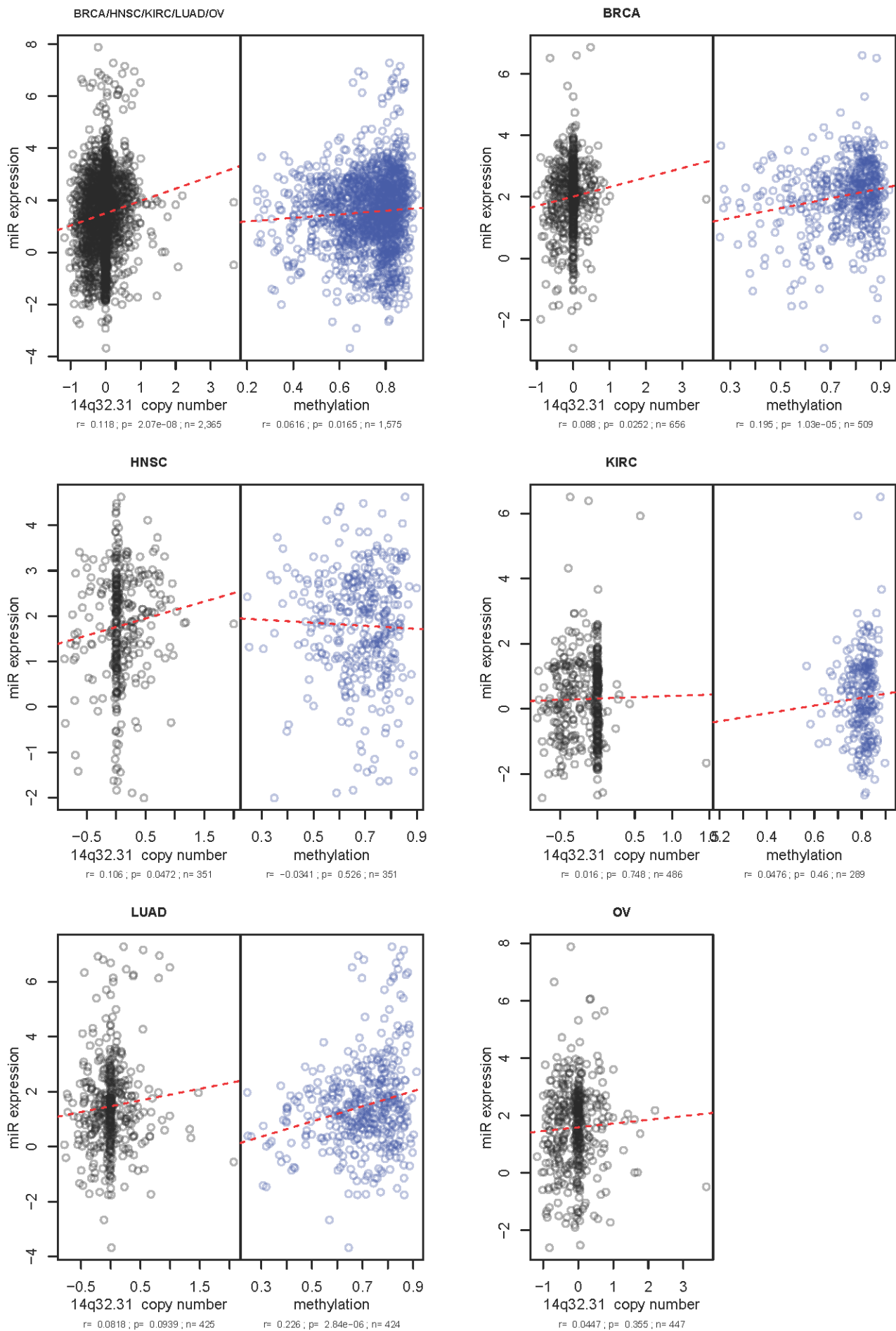

Supplement: S3 Fig — (PDF) [file pone.0140072.s003.pdf]

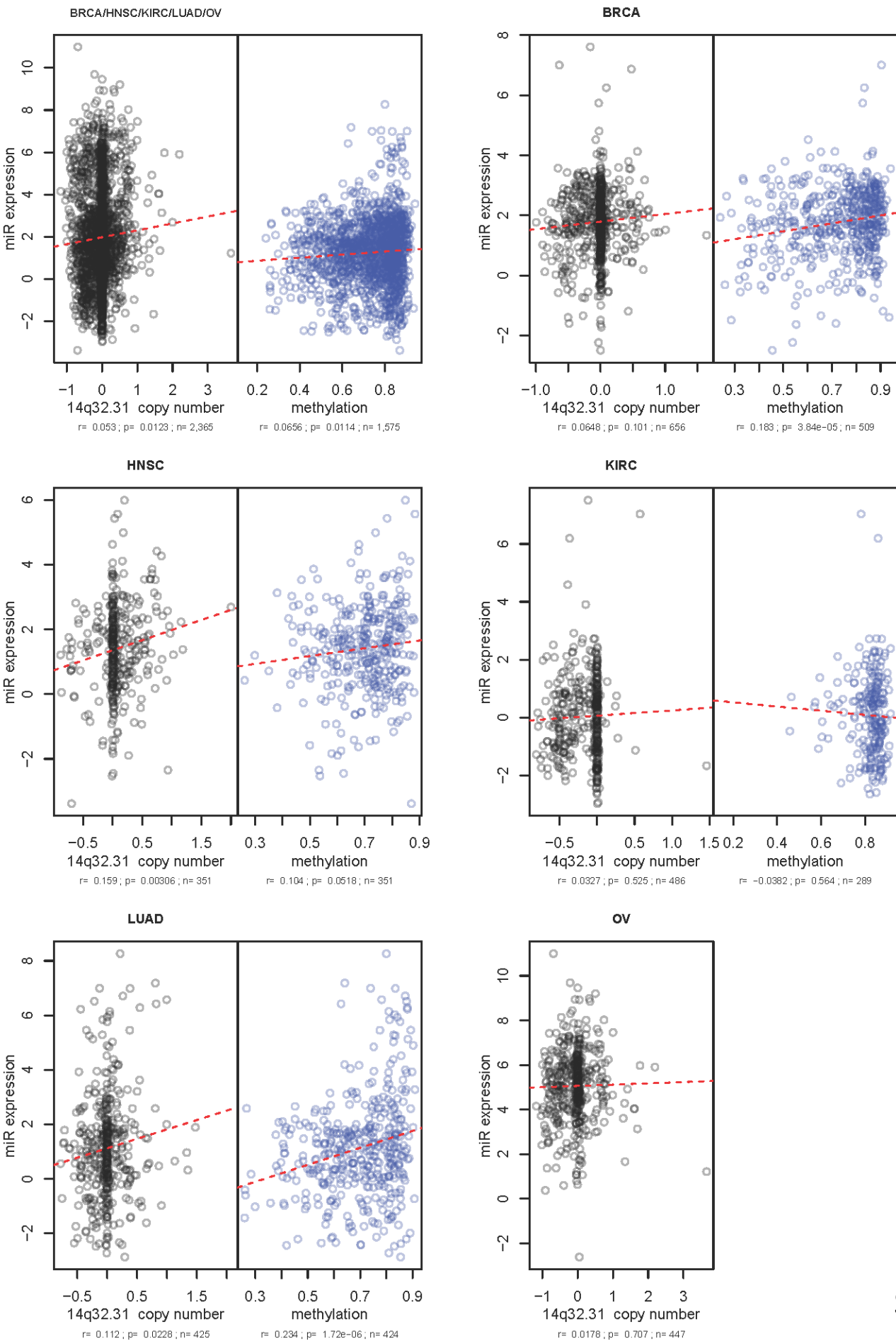

Supplement: S4 Fig — (PDF) [file pone.0140072.s004.pdf]

# hsa-mir-24-1

BRCA/HNSC/KIRC/LUAD/OV

BRCA

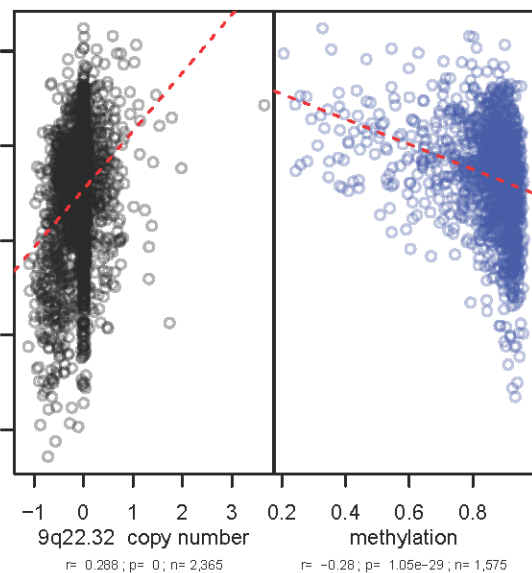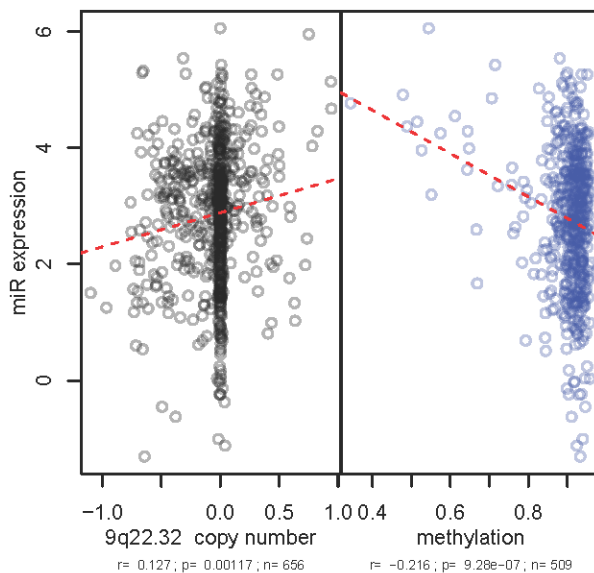

HNSC

KIRC

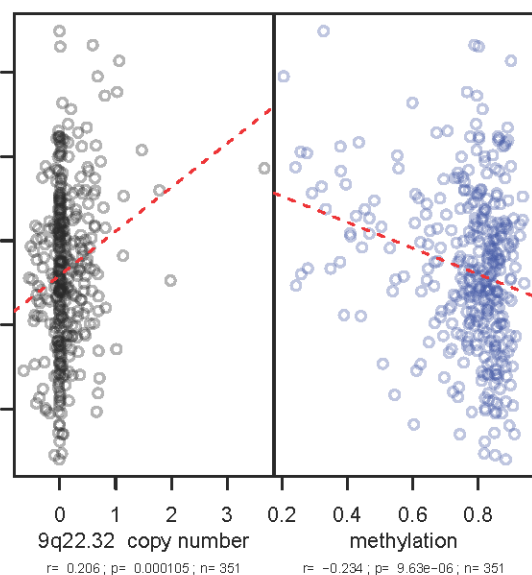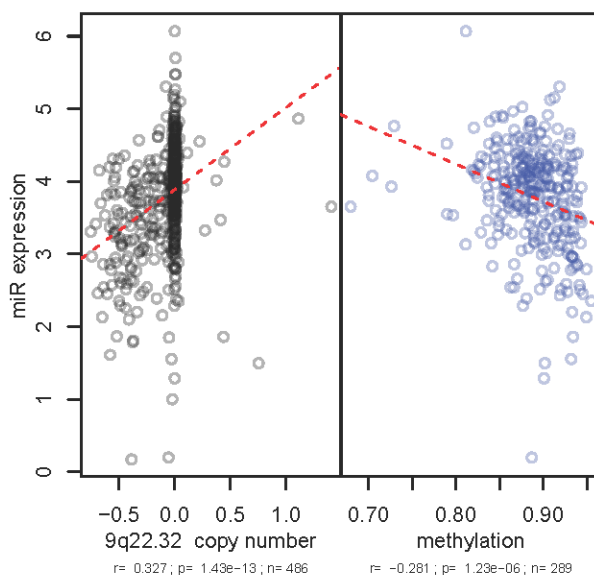

LUAD

OV

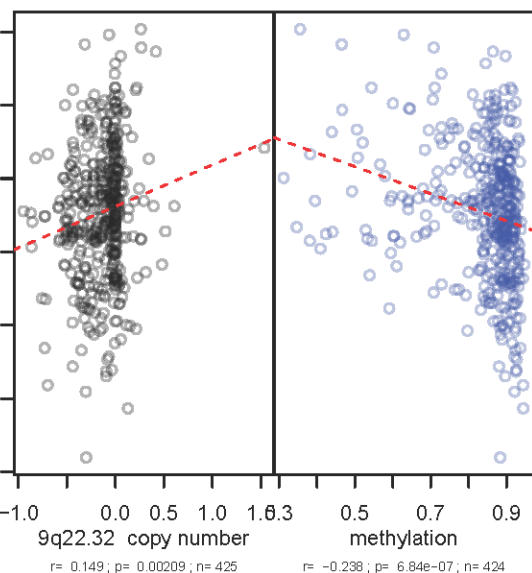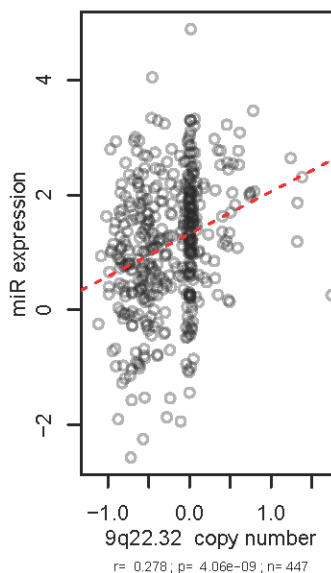

Supplement: S5 Fig — (PDF) [file pone.0140072.s005.pdf]

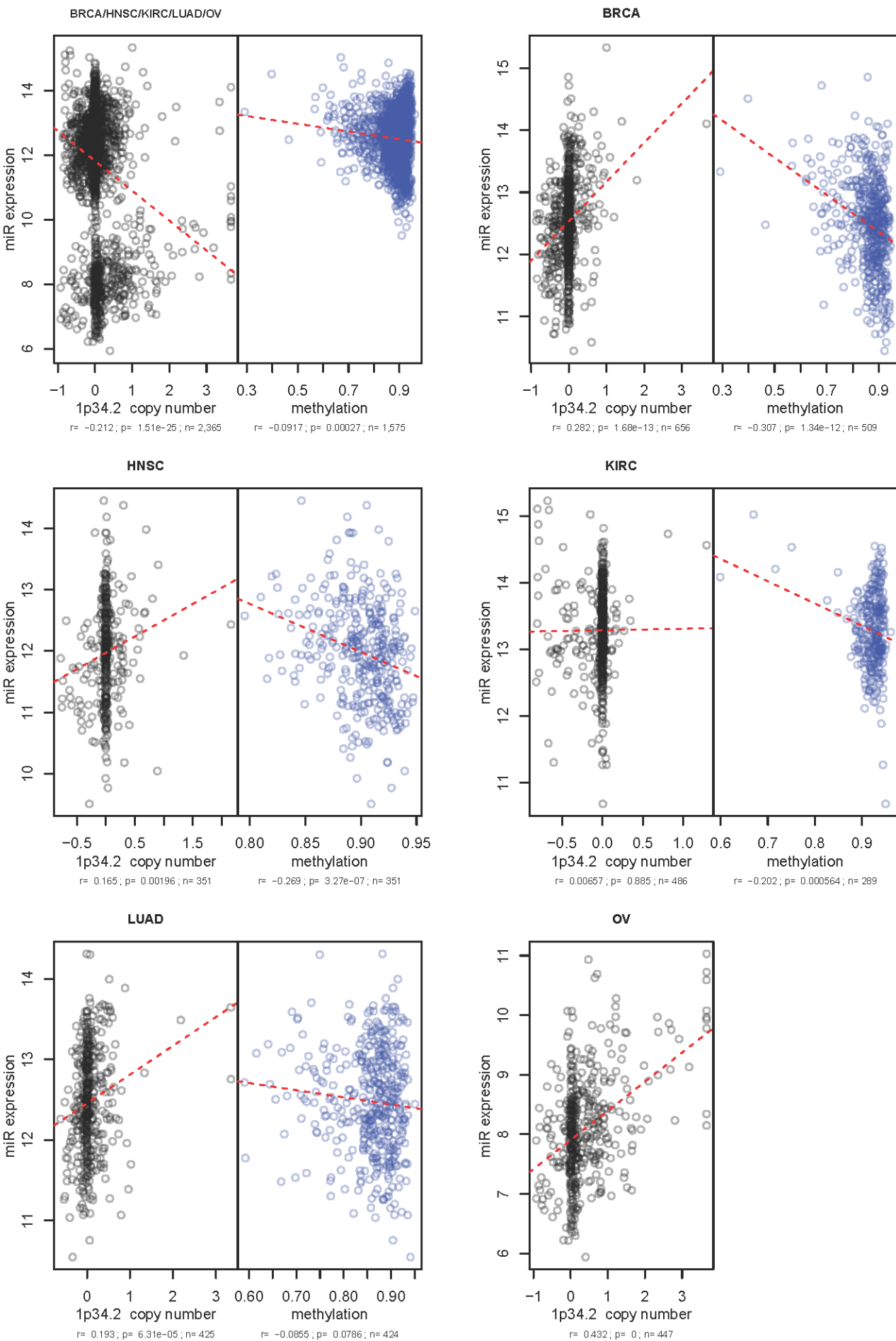

Supplement: S6 Fig — (PDF) [file pone.0140072.s006.pdf]

miR15b

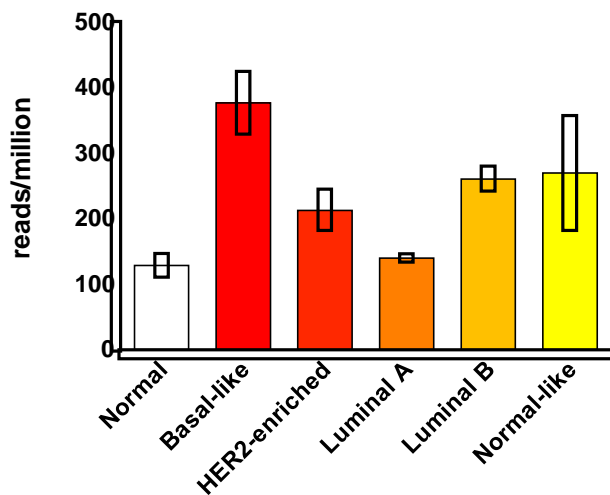

miR24-1\*

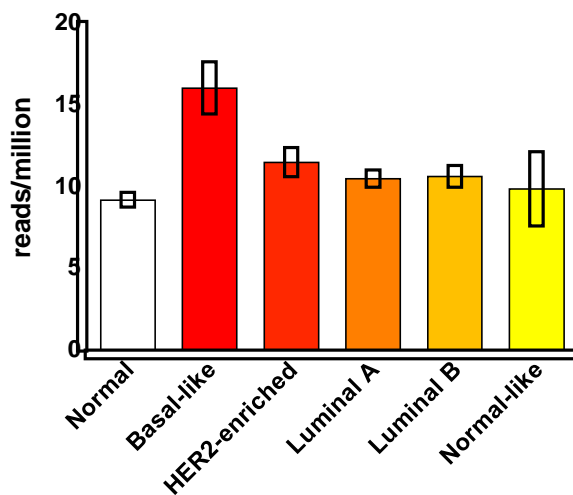

miR30e

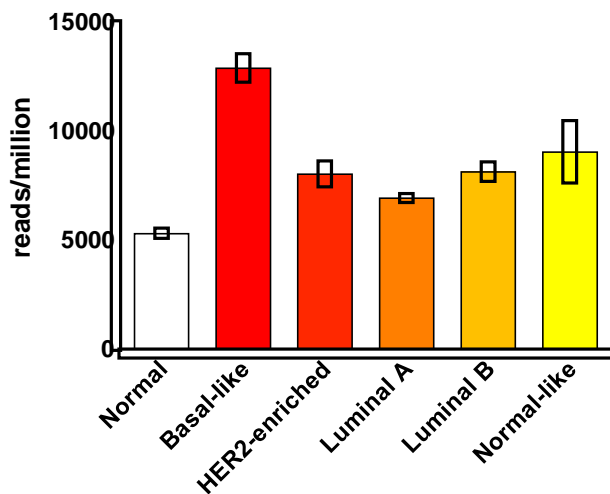

| mir               | Basal | LumA | LumB | Her2 |
|-------------------|-------|------|------|------|
| hsa-mir-15b       | PS    | PS   | GS   |      |
| hsa-mir-24-1-star | GS    | PS   | PS   |      |
| hsa-mir-30e       | PS    | PS   | PS   | PS   |

Supplement: S7 Fig — (PDF) [file pone.0140072.s007.pdf]

# miR-487b -- LUAD

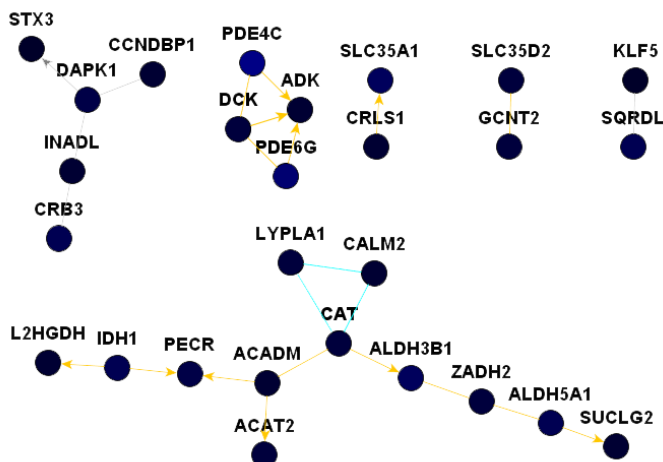

# miR-487b -- HNSC

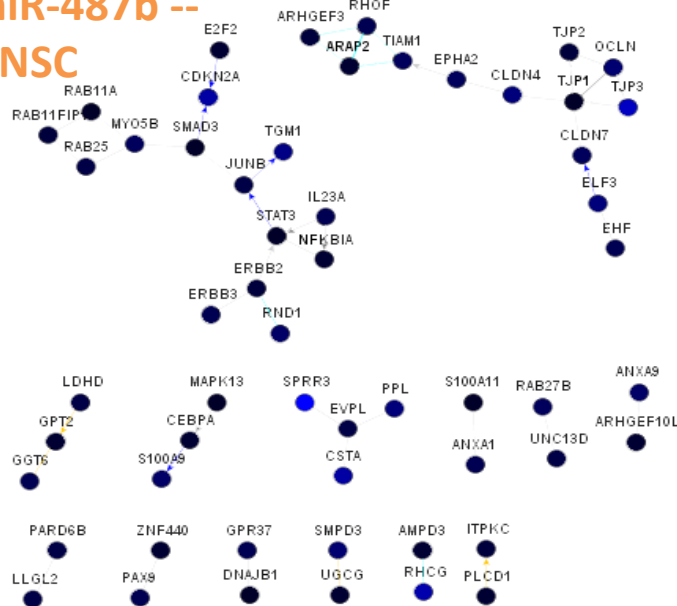

# miR-487b -- KIRC

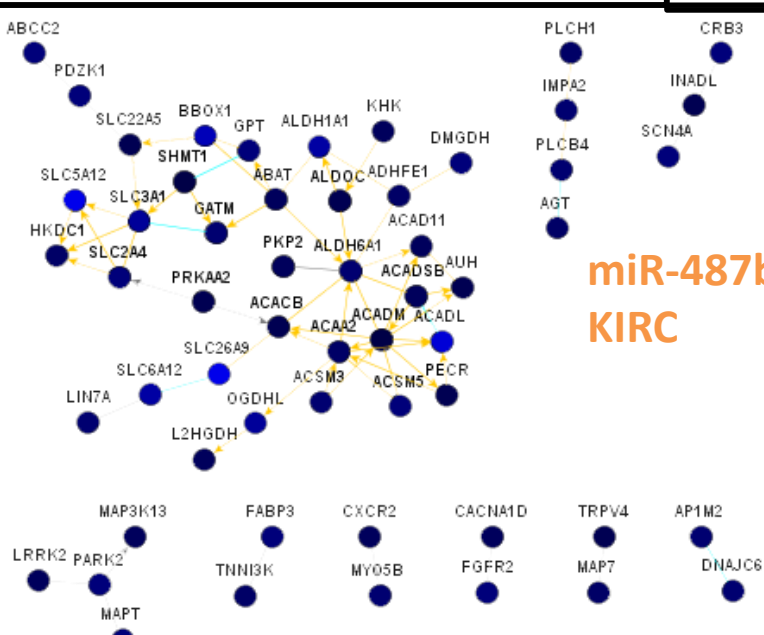

# miR-487b -- OVCA

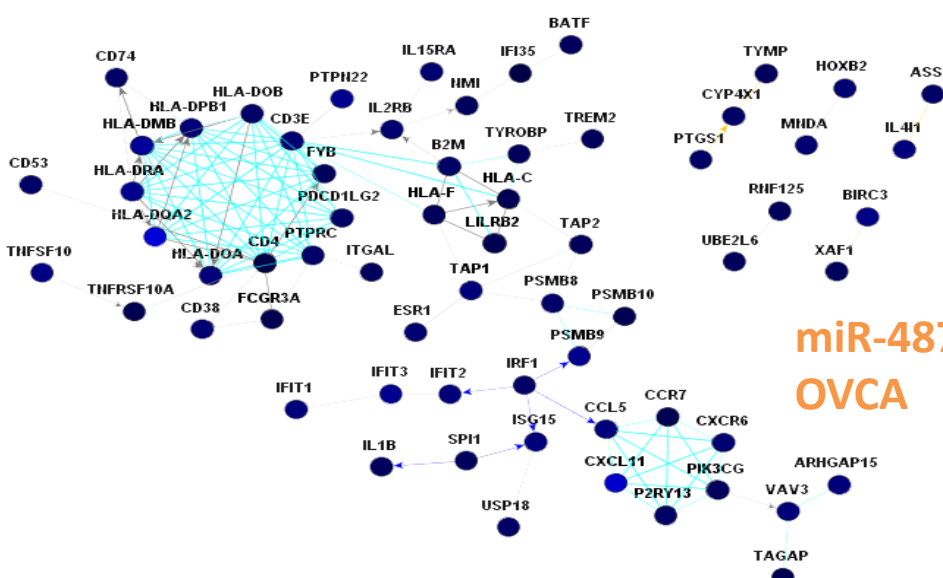

Supplement: S9 Fig — (PDF) [file pone.0140072.s009.pdf]

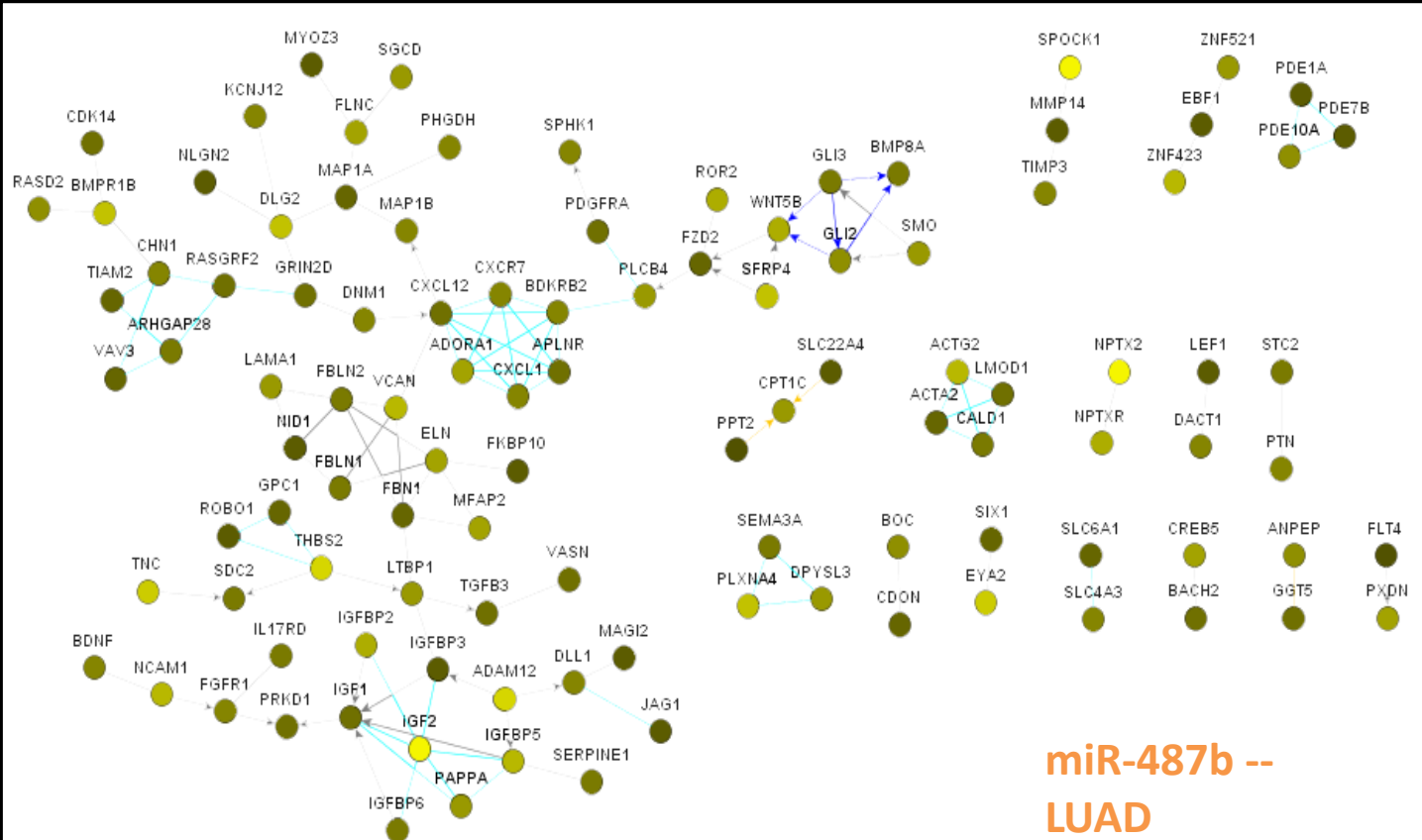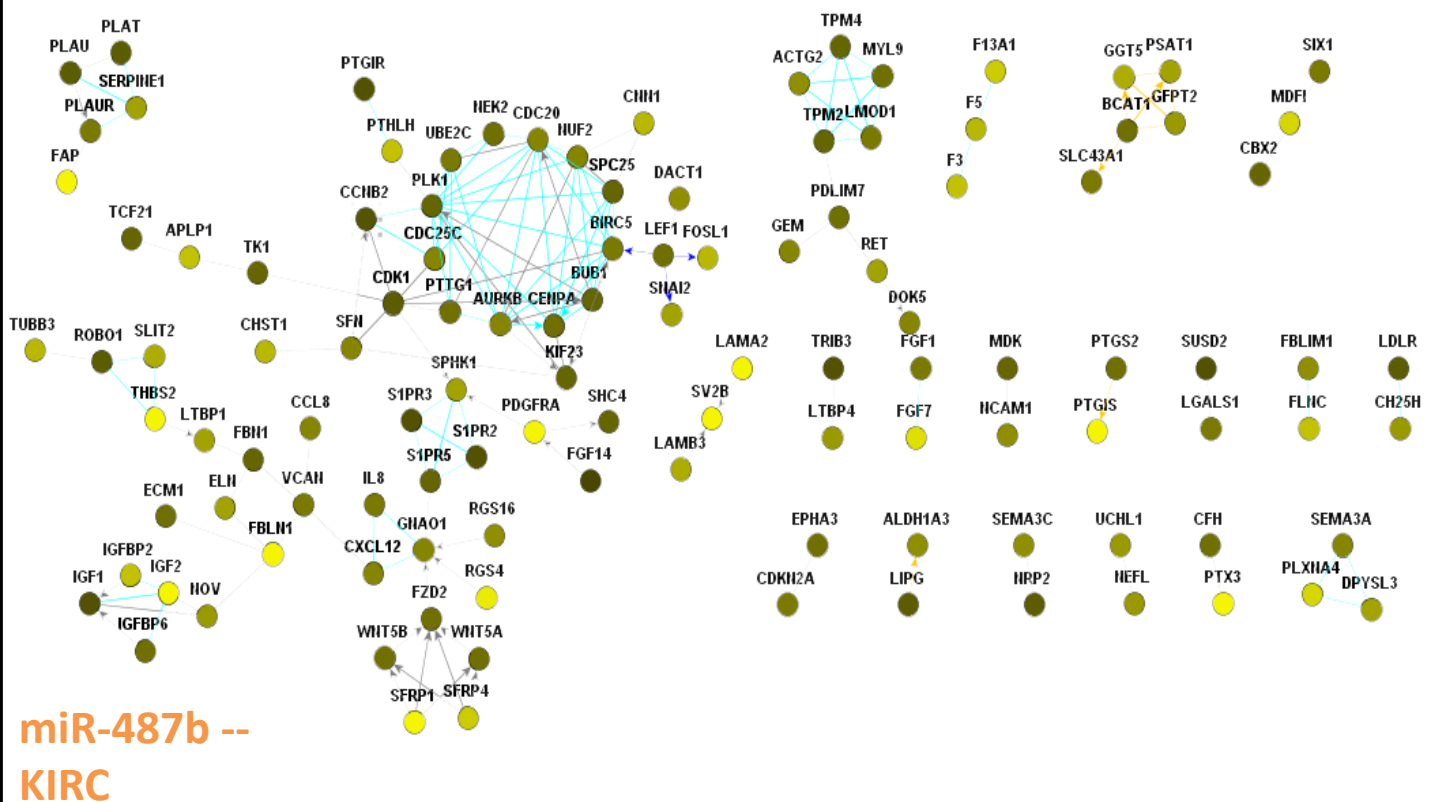

Supplement: S11 Fig — (PDF) [file pone.0140072.s011.pdf]

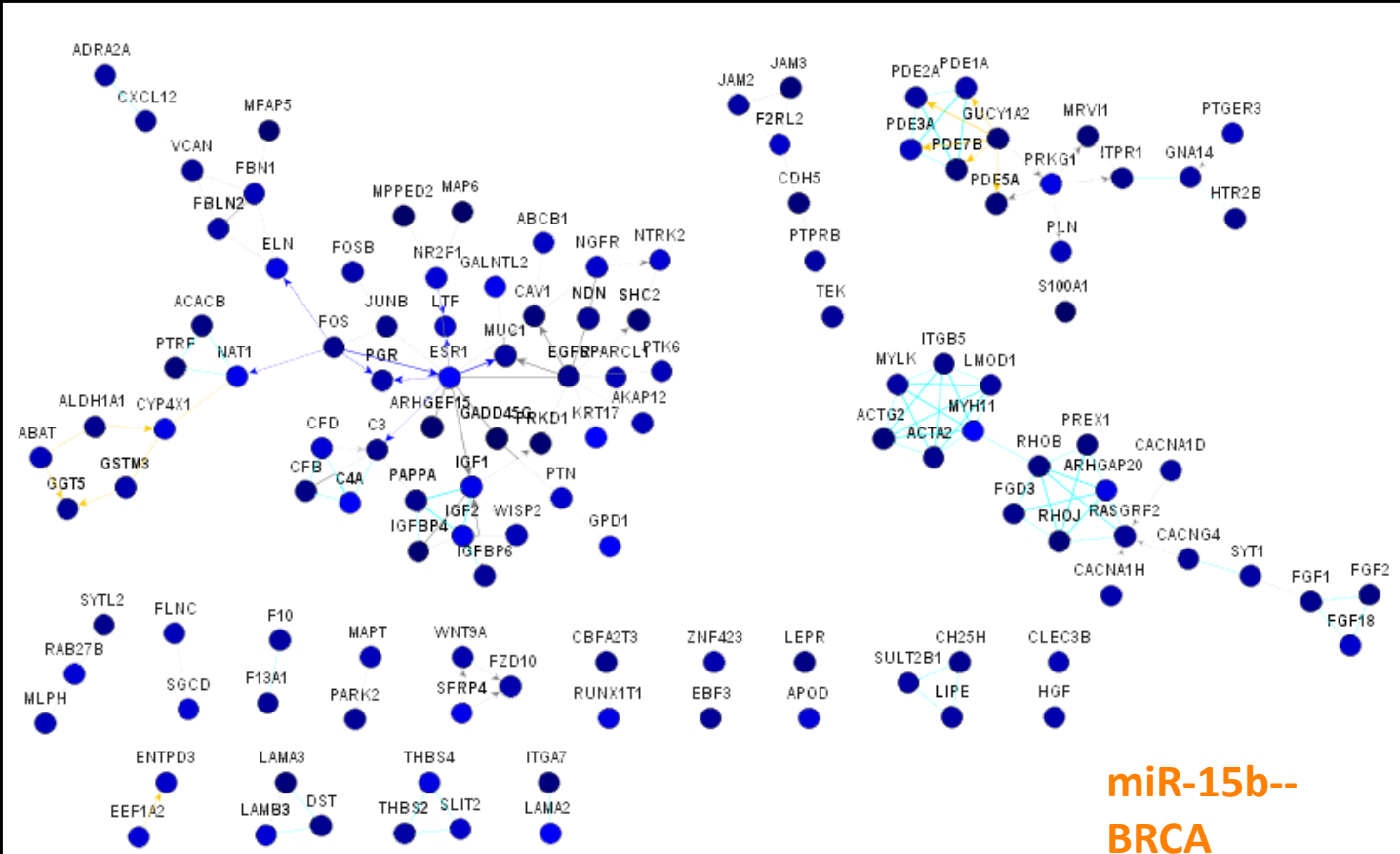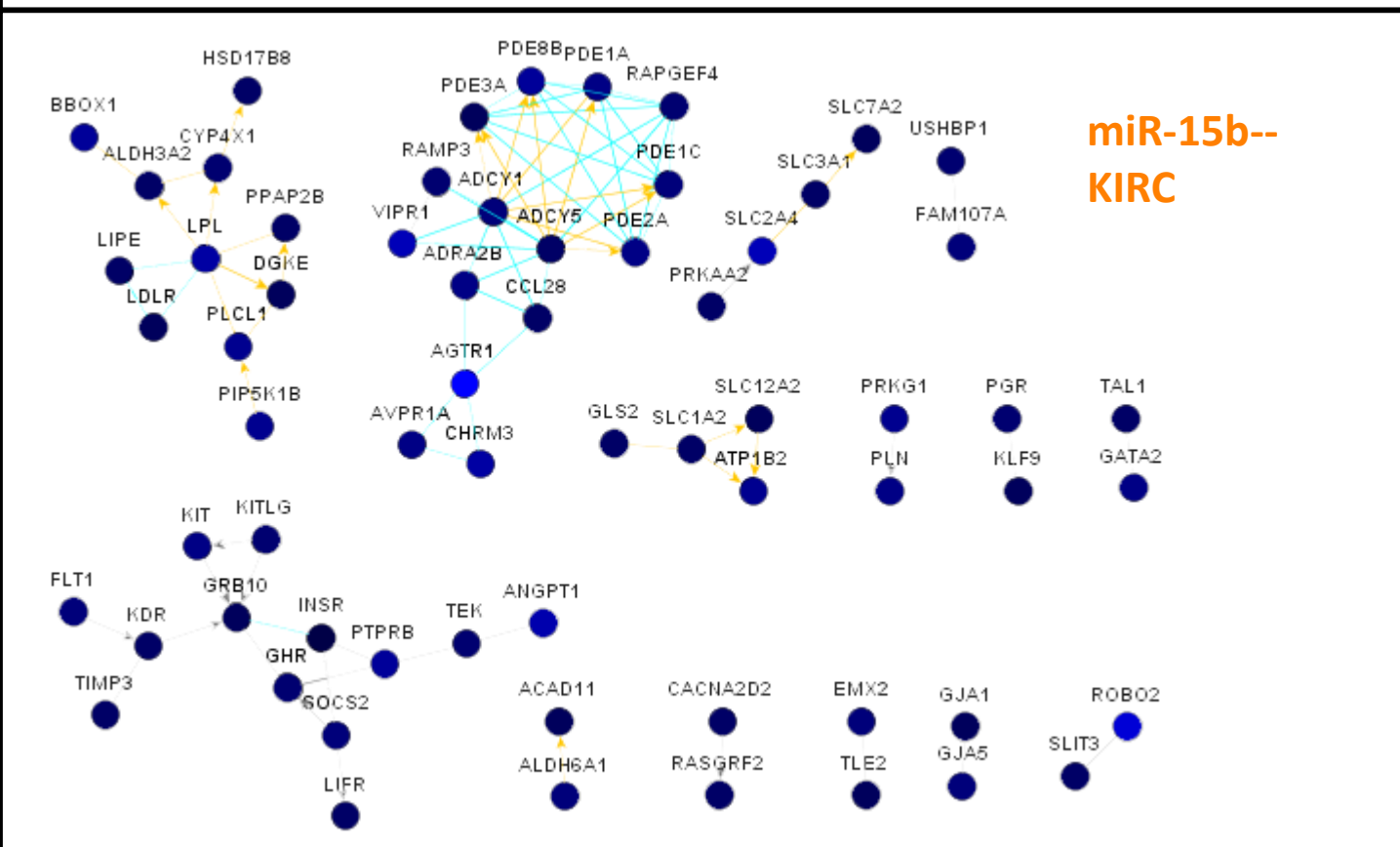

Supplement: S12 Fig — (PDF) [file pone.0140072.s012.pdf]

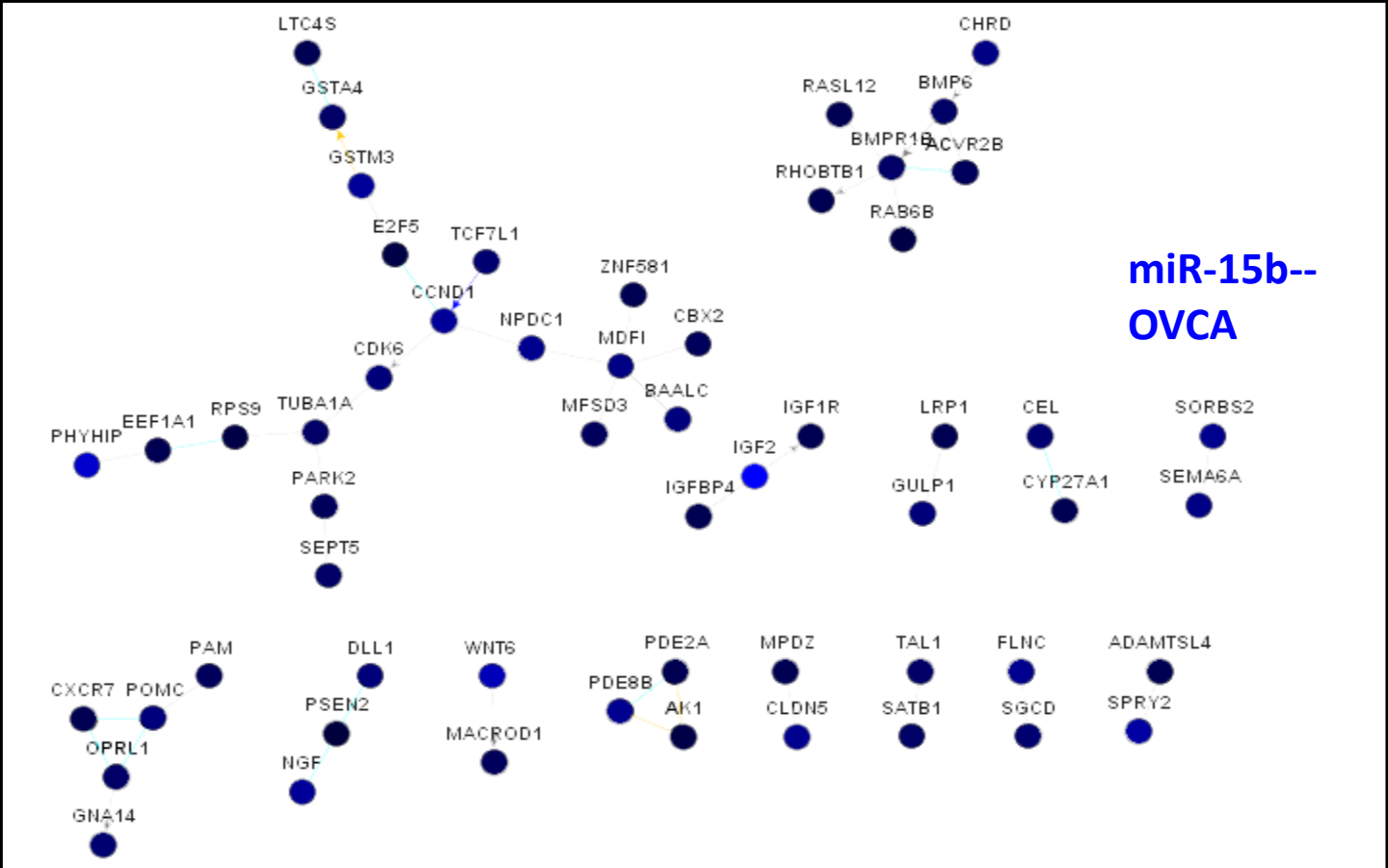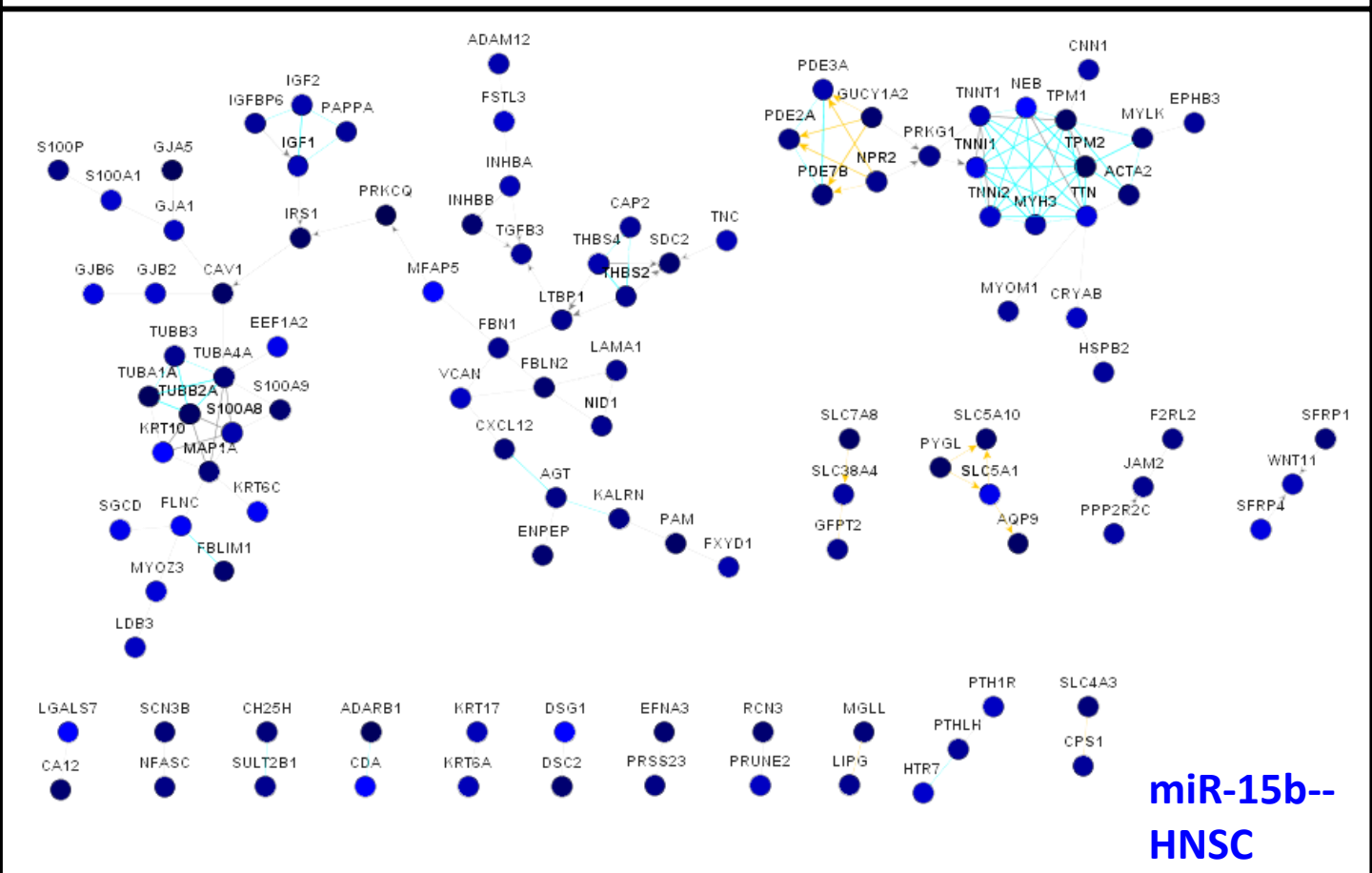

Supplement: S13 Fig — (PDF) [file pone.0140072.s013.pdf]

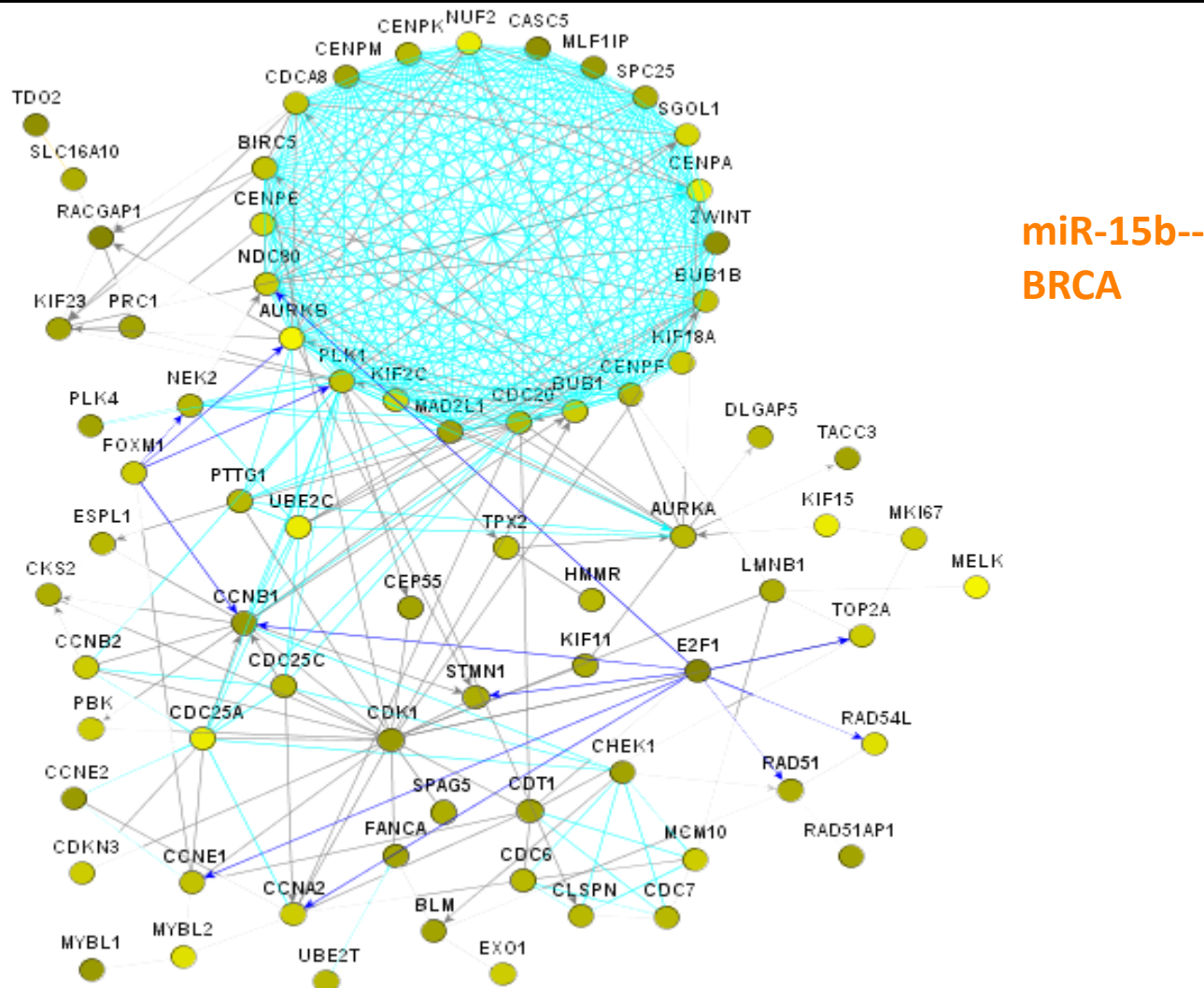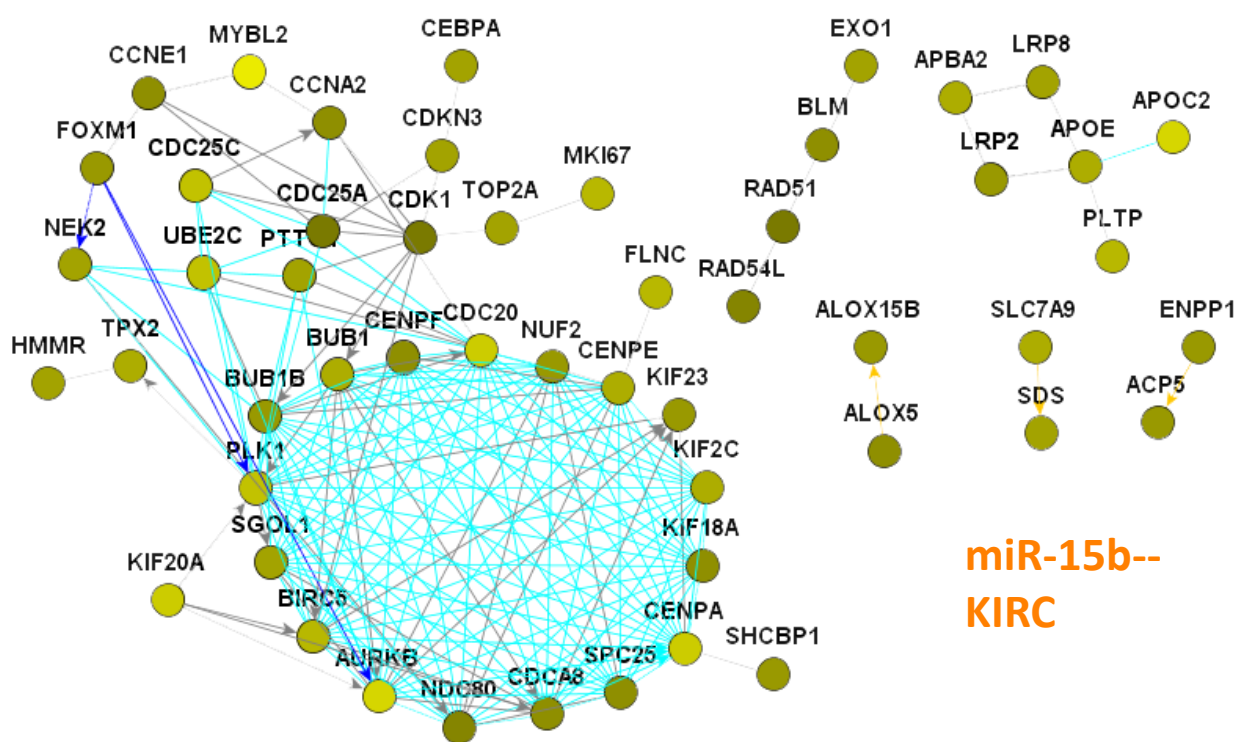

Supplement: S14 Fig — (PDF) [file pone.0140072.s014.pdf]

**miR-15b--  
OVCA**

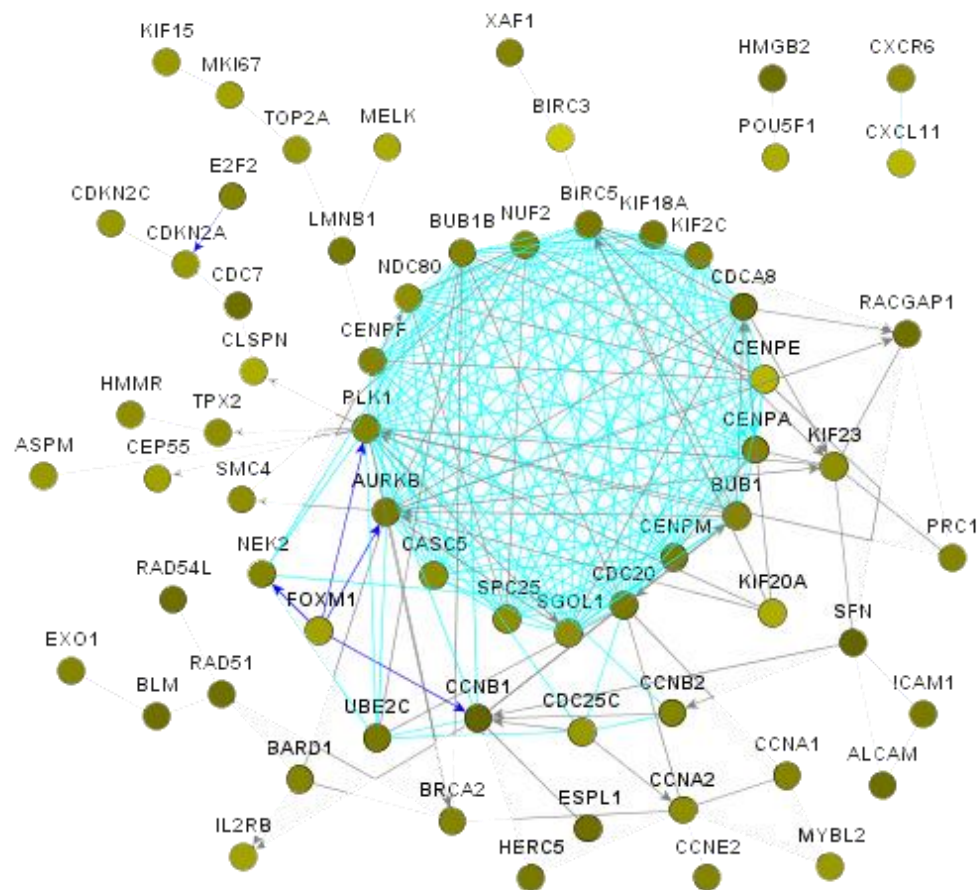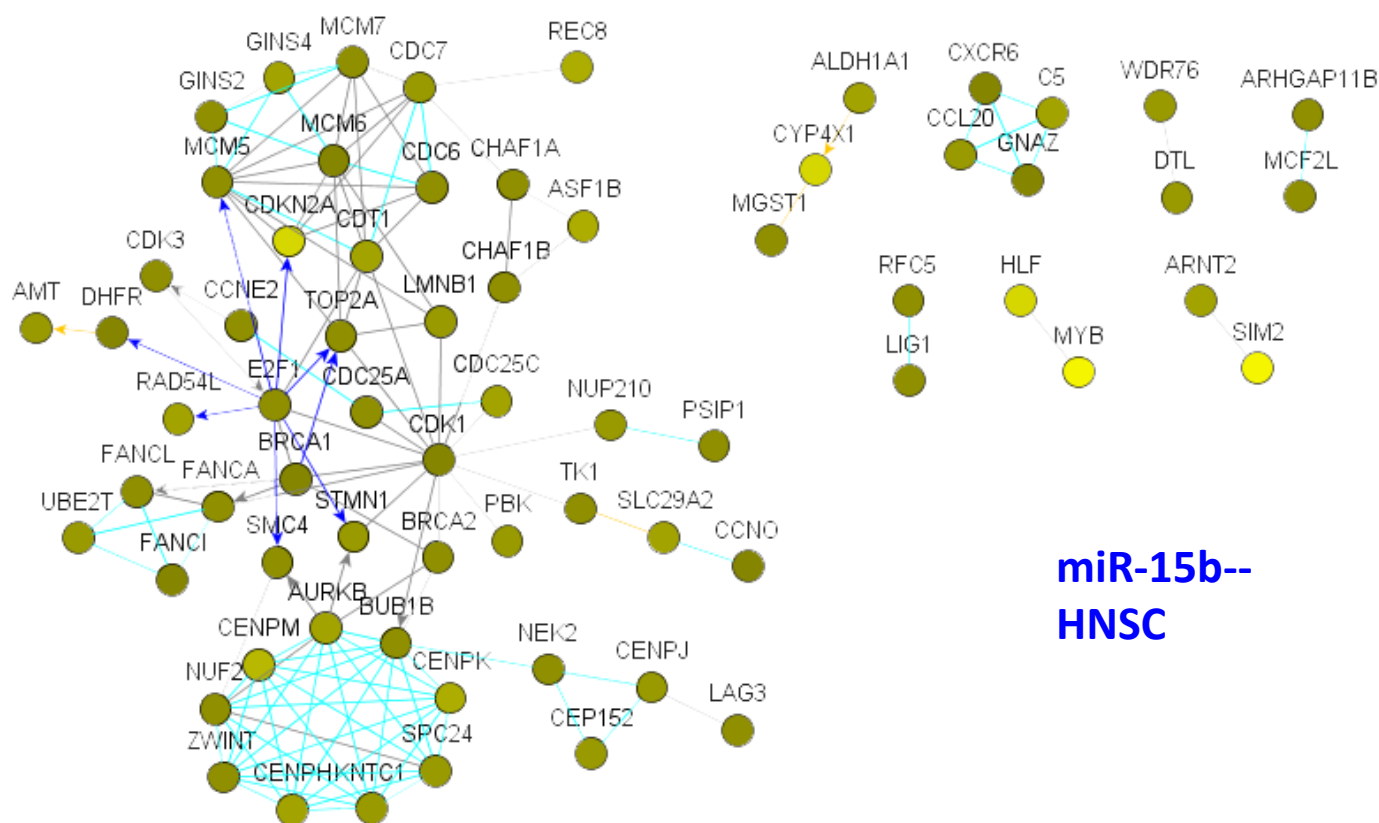

**miR-15b--  
HNSC**

Supplement: S15 Fig — (PDF) [file pone.0140072.s015.pdf]

**miR-24-1\*--  
BRCA**

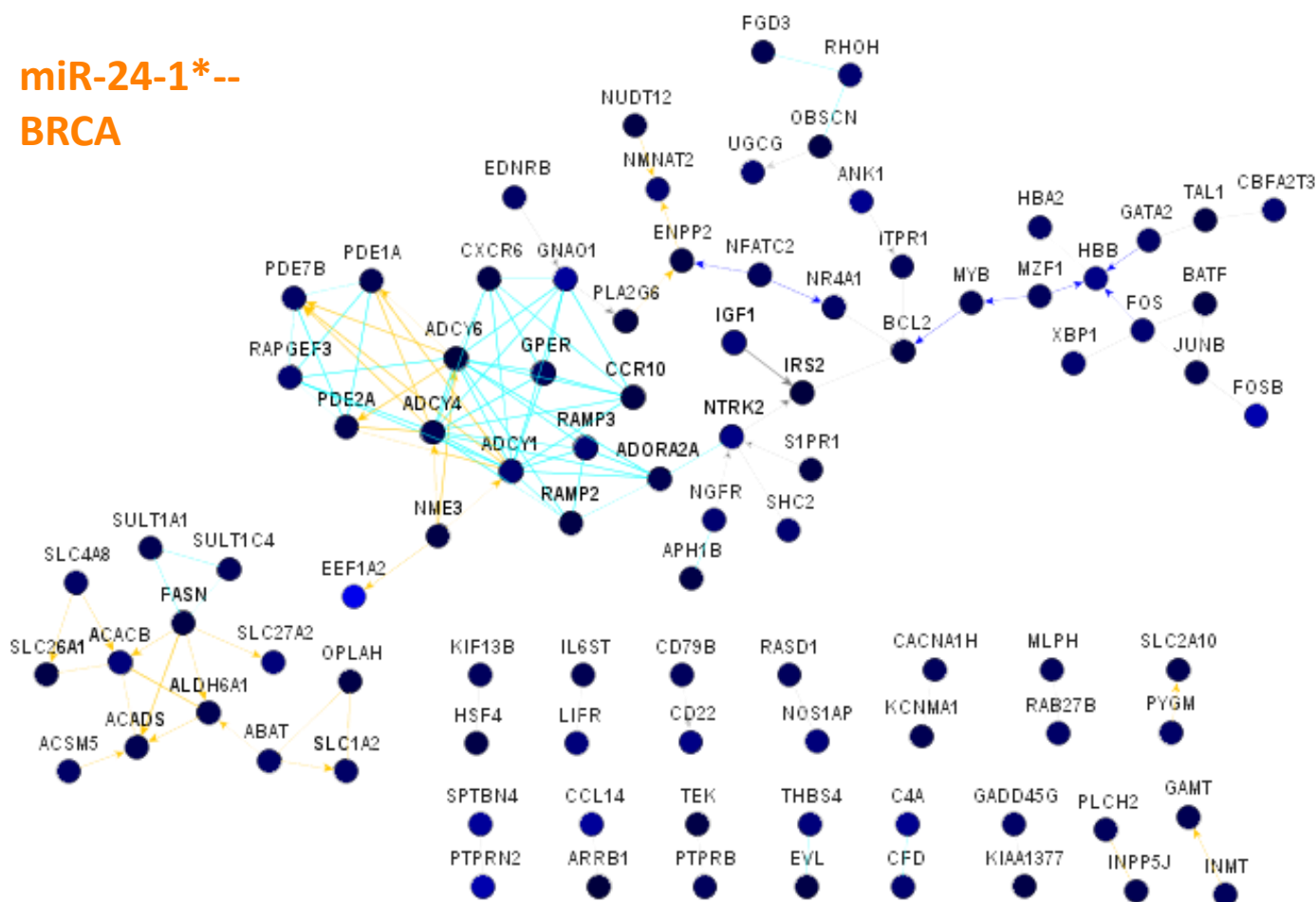

**miR-24-1\*--  
KIRC**

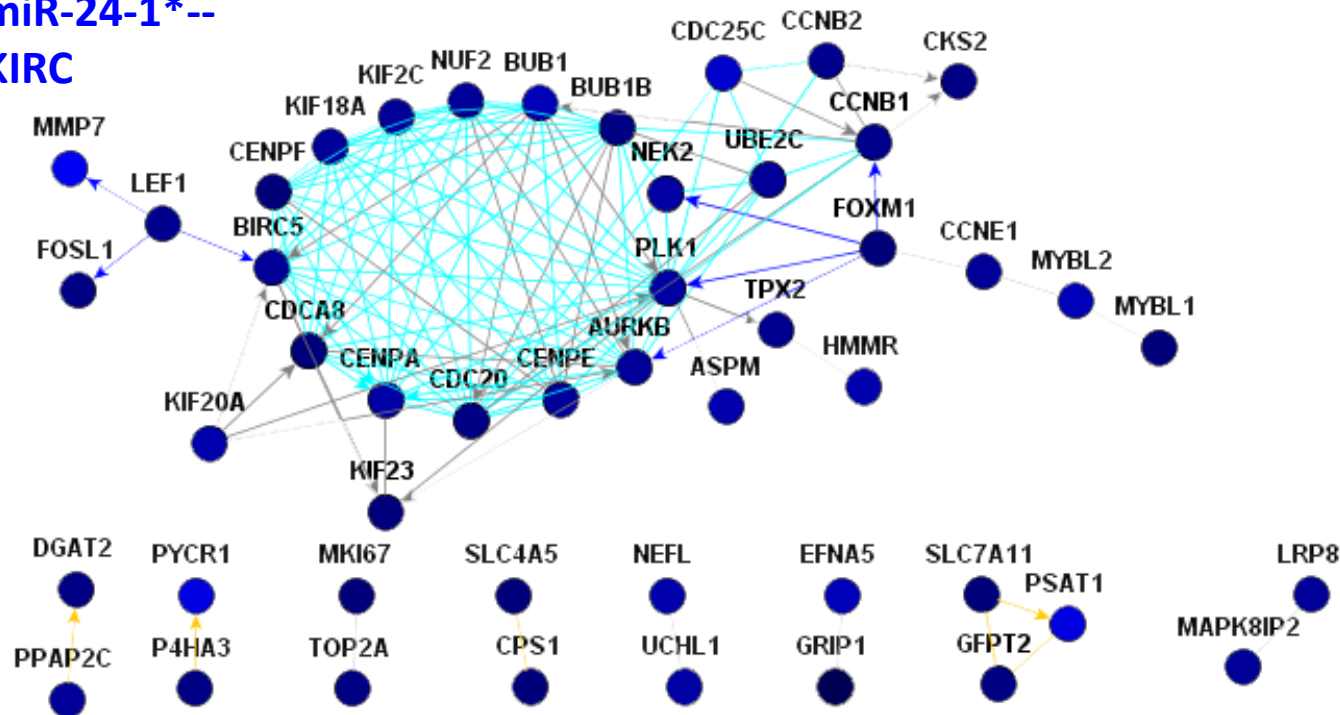

Supplement: S16 Fig — (PDF) [file pone.0140072.s016.pdf]

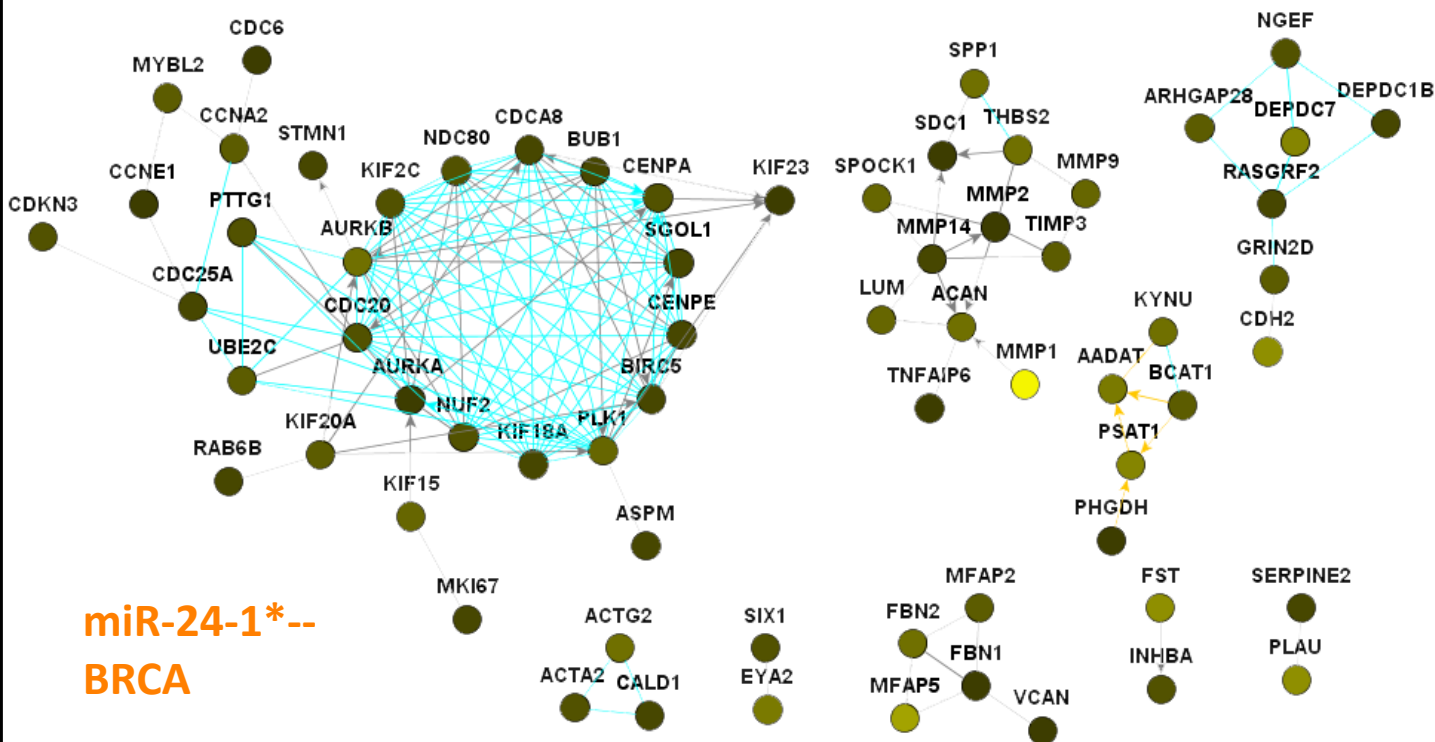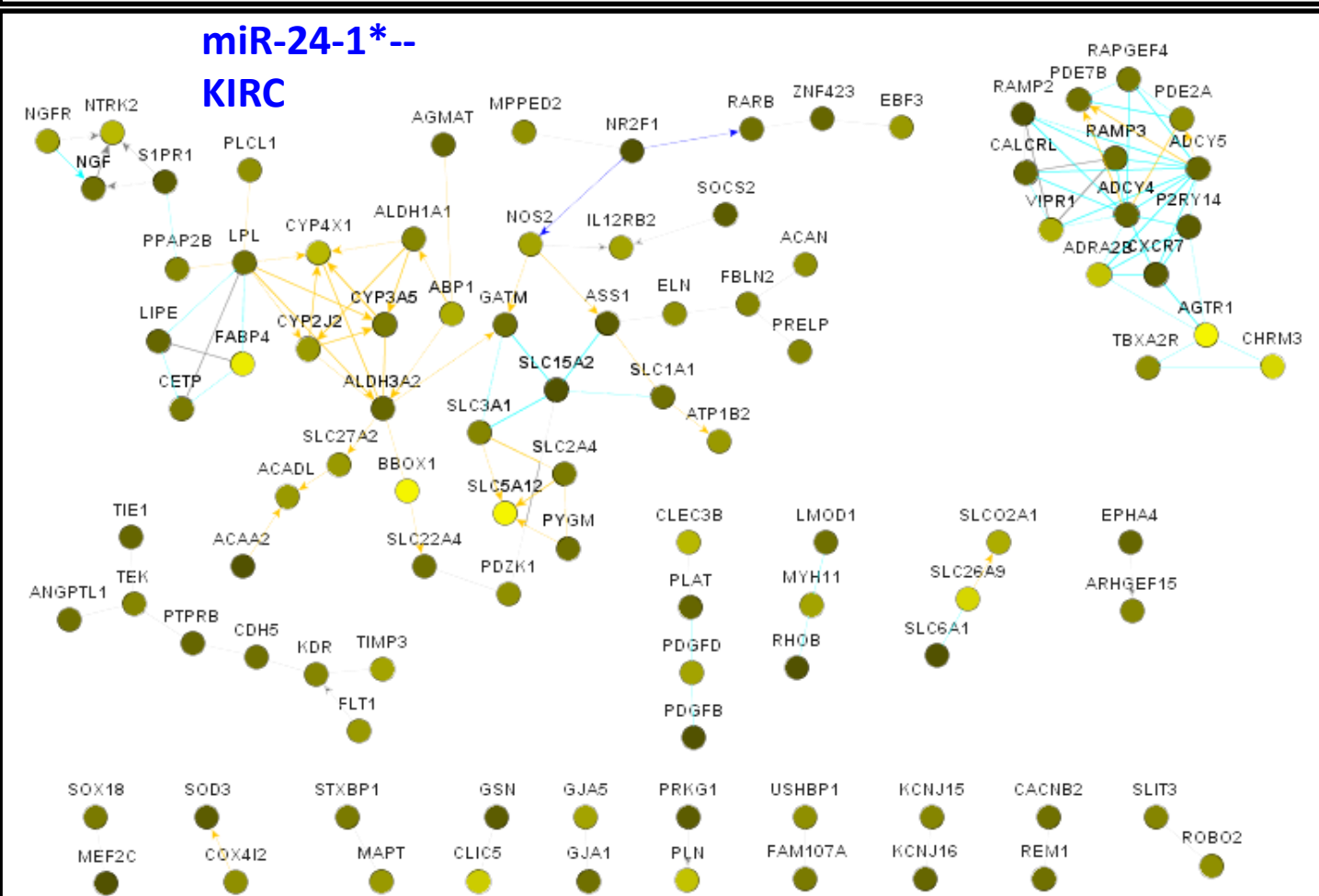

Supplement: S18 Fig — (PDF) [file pone.0140072.s018.pdf]

**miR-485--  
KIRC**

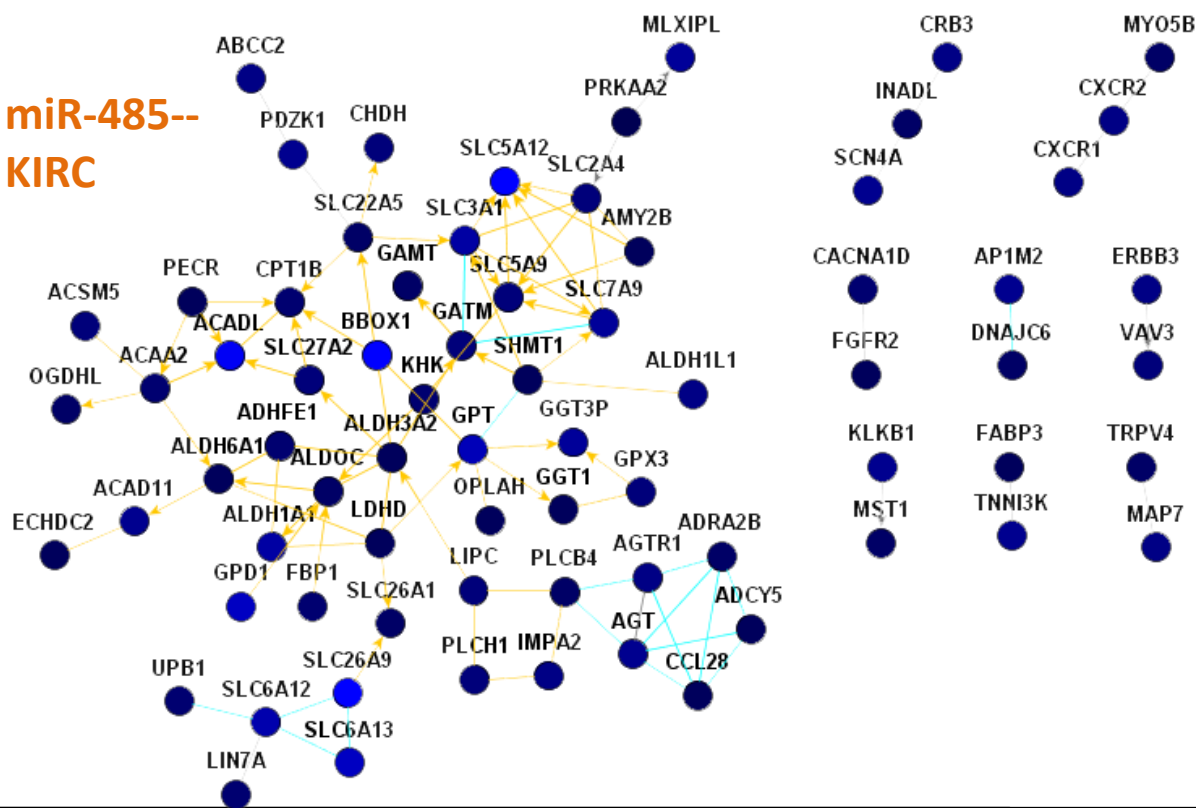

**miR-485--  
HNSC**

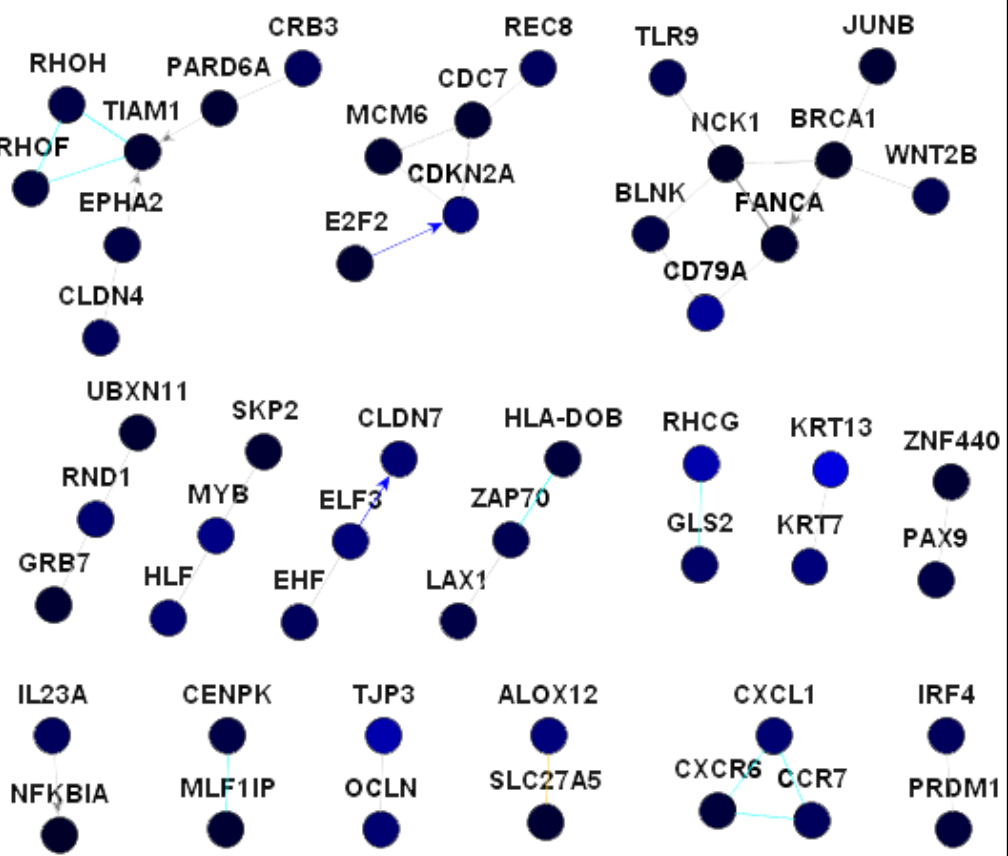

Supplement: S20 Fig — (PDF) [file pone.0140072.s020.pdf]

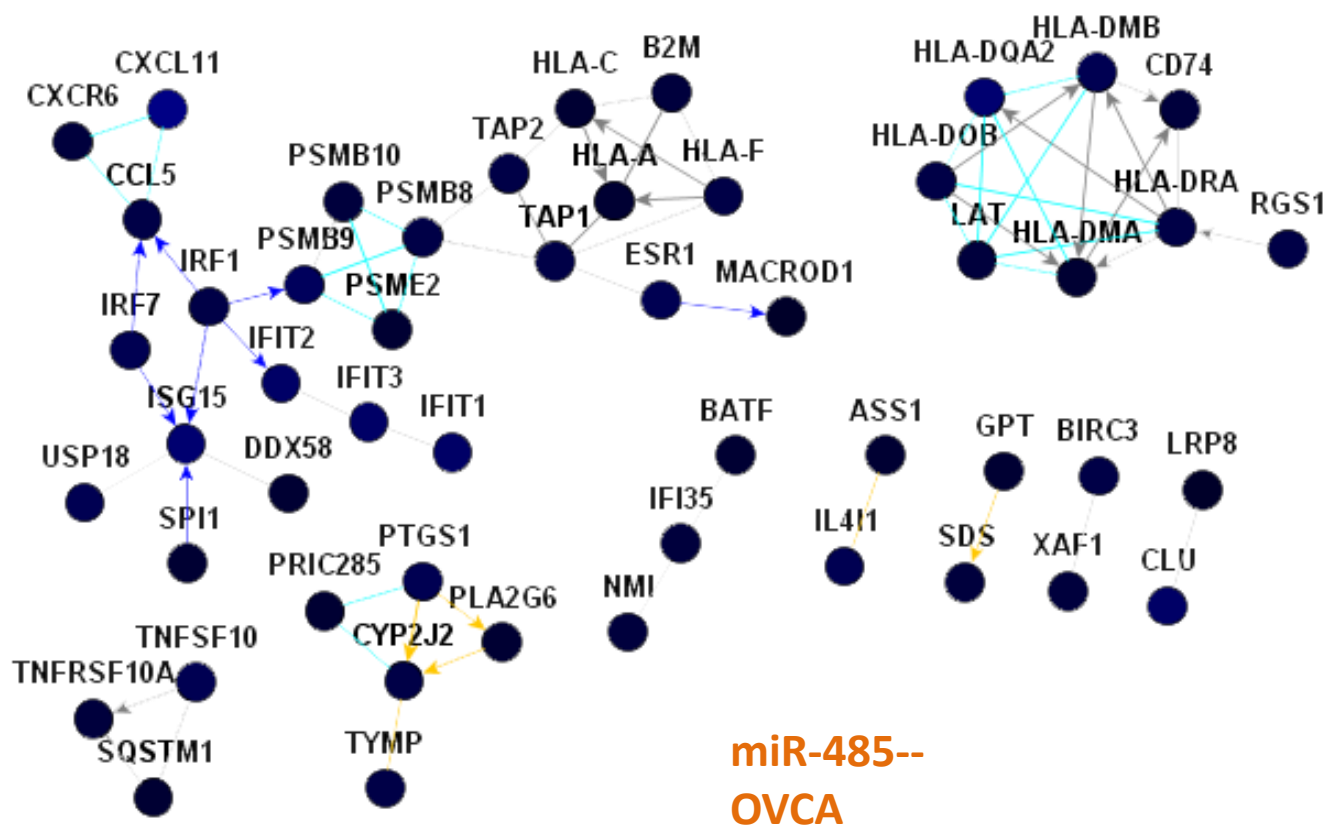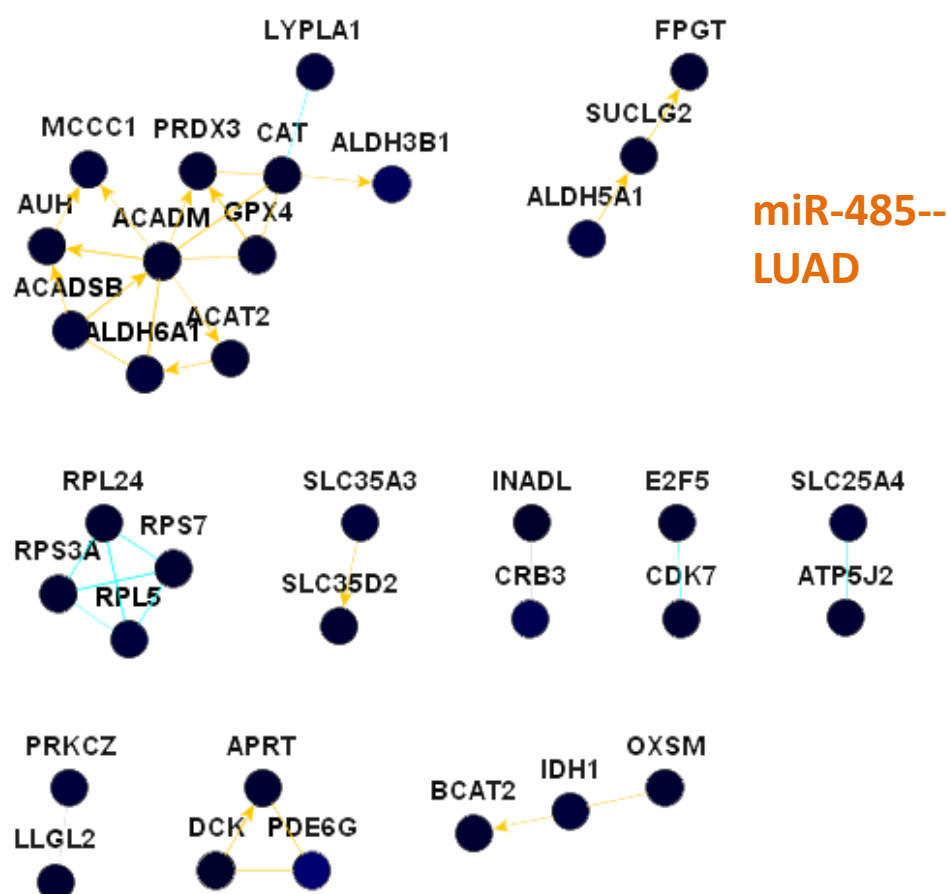

Supplement: S21 Fig — (PDF) [file pone.0140072.s021.pdf]

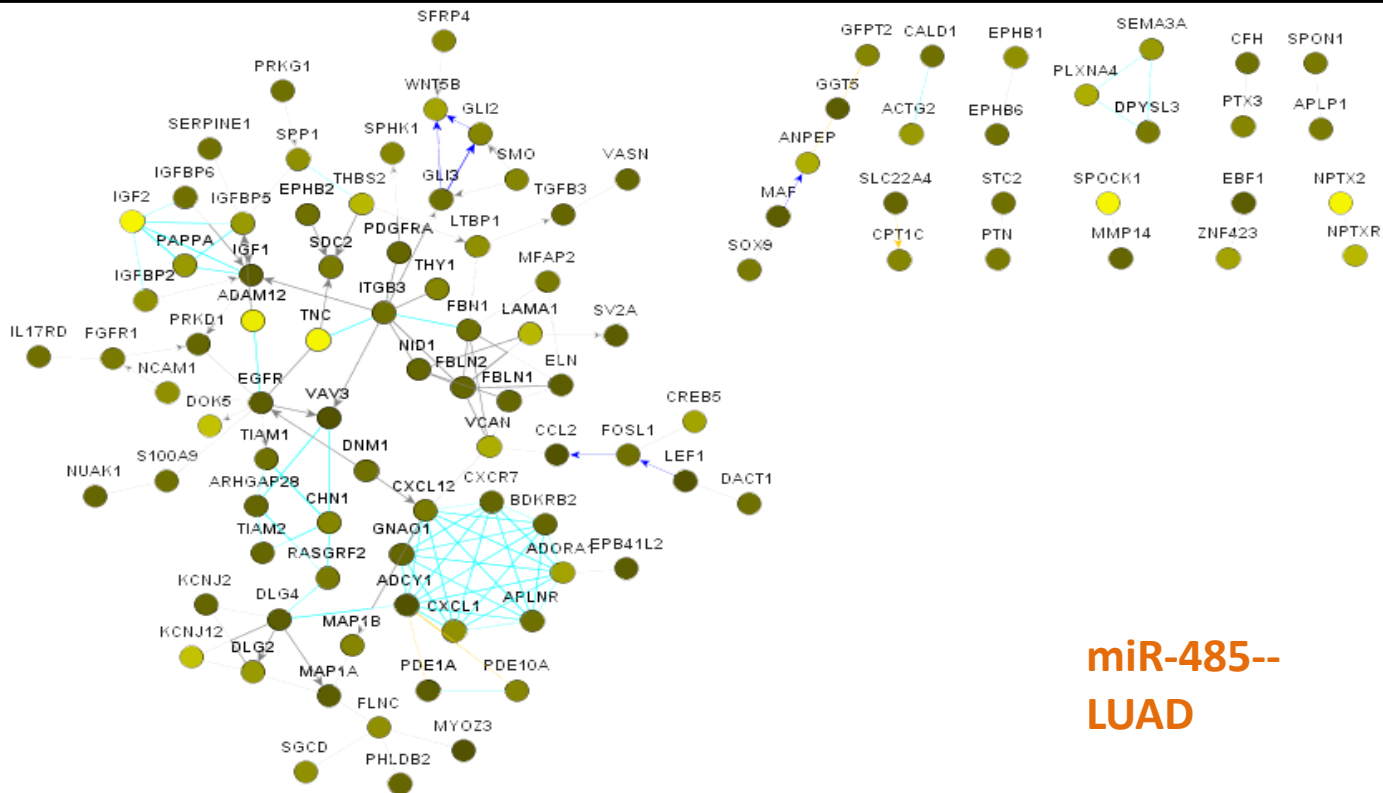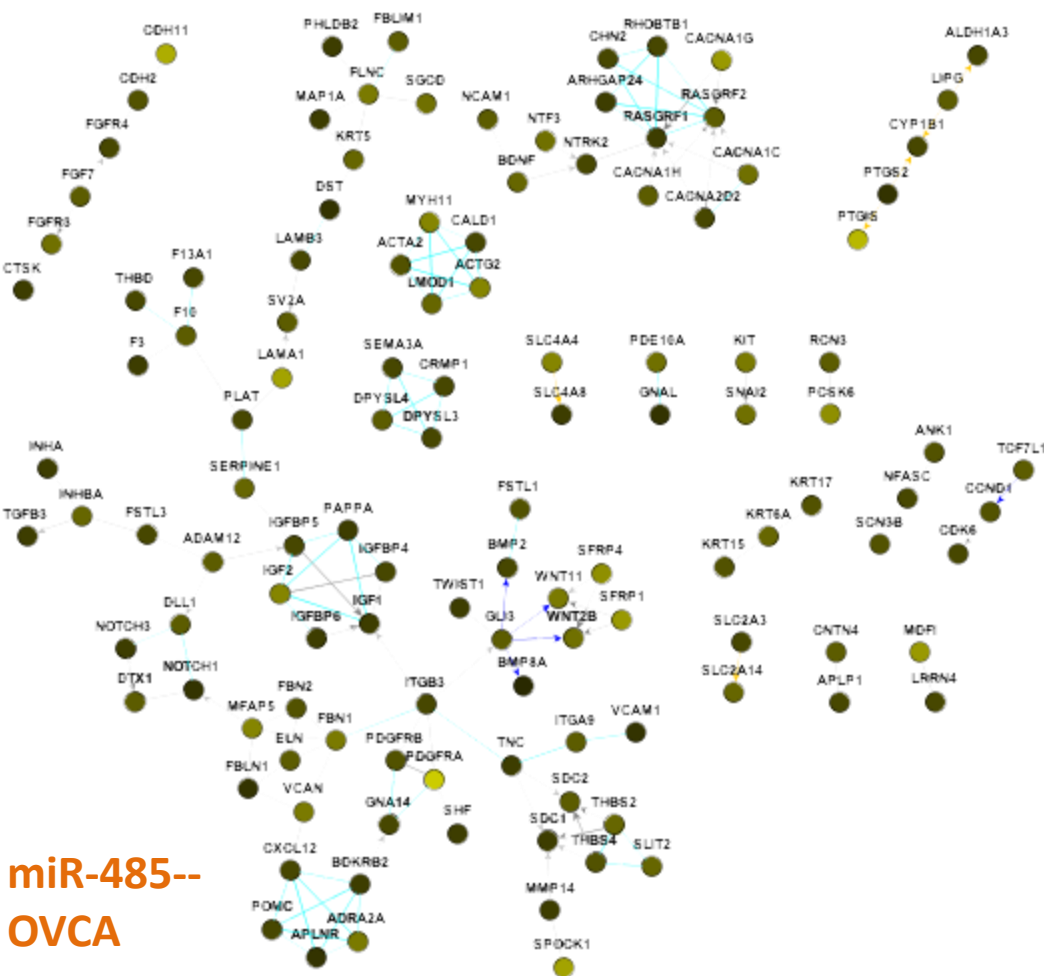

Supplement: S23 Fig — (PDF) [file pone.0140072.s023.pdf]

miR-30e --  
BRCA

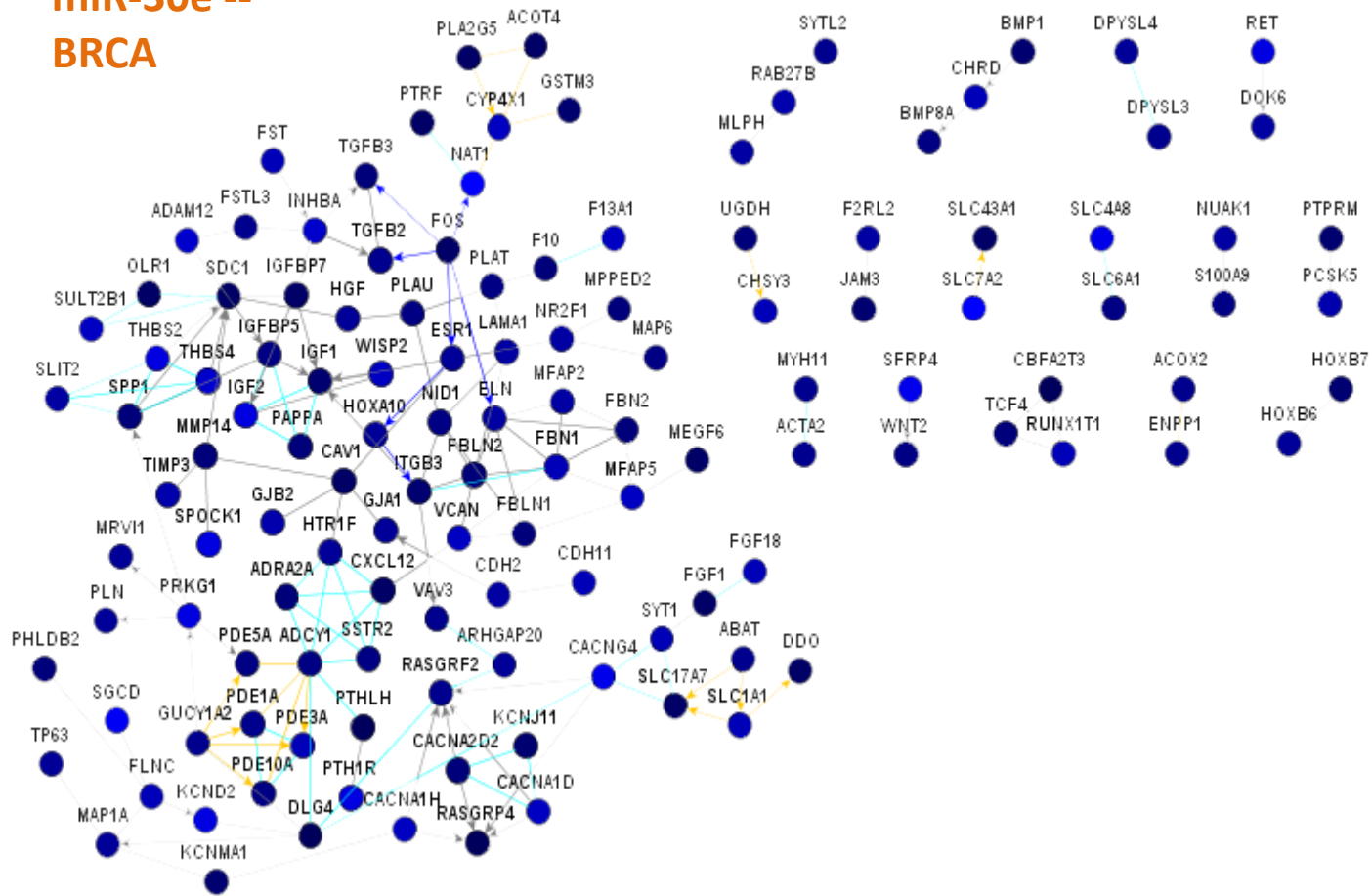

miR-30e --  
BRCA

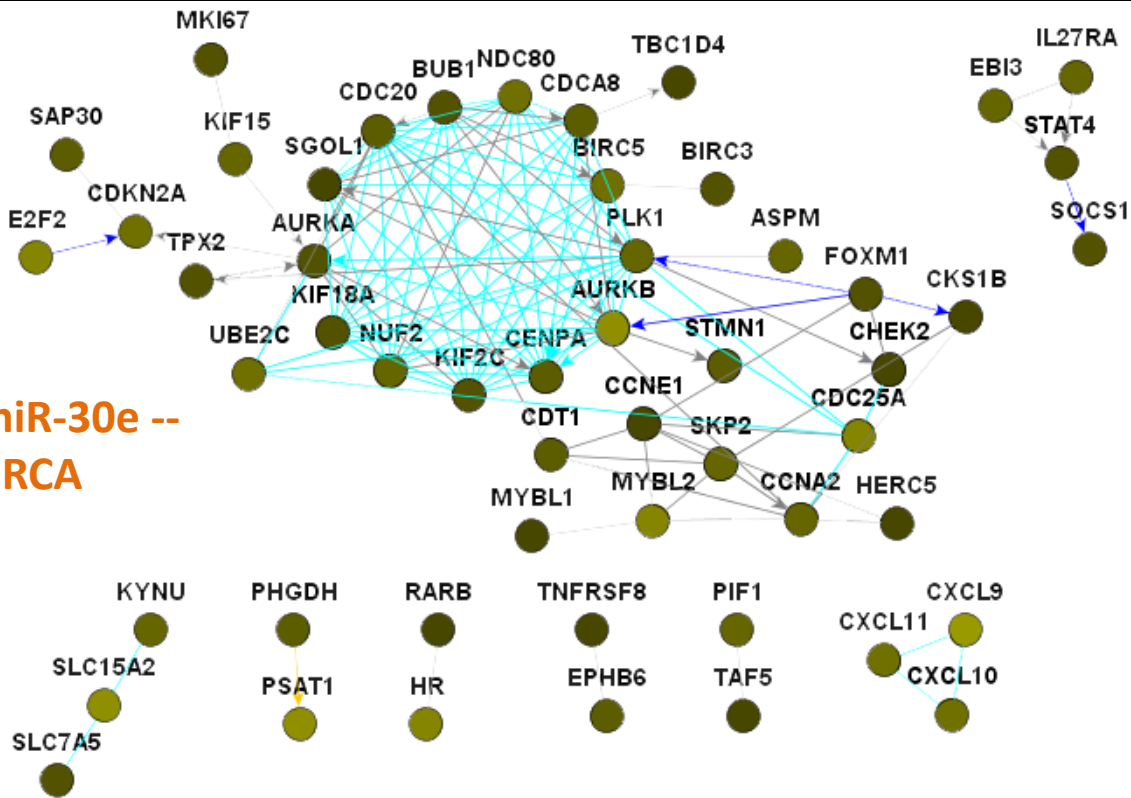

Supplement: S24 Fig — (PDF) [file pone.0140072.s024.pdf]

## miR-30e -- HNSC

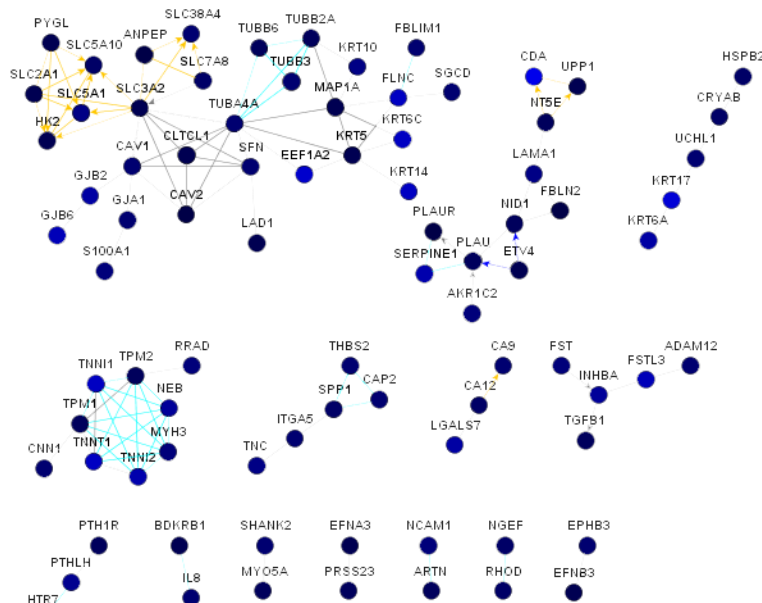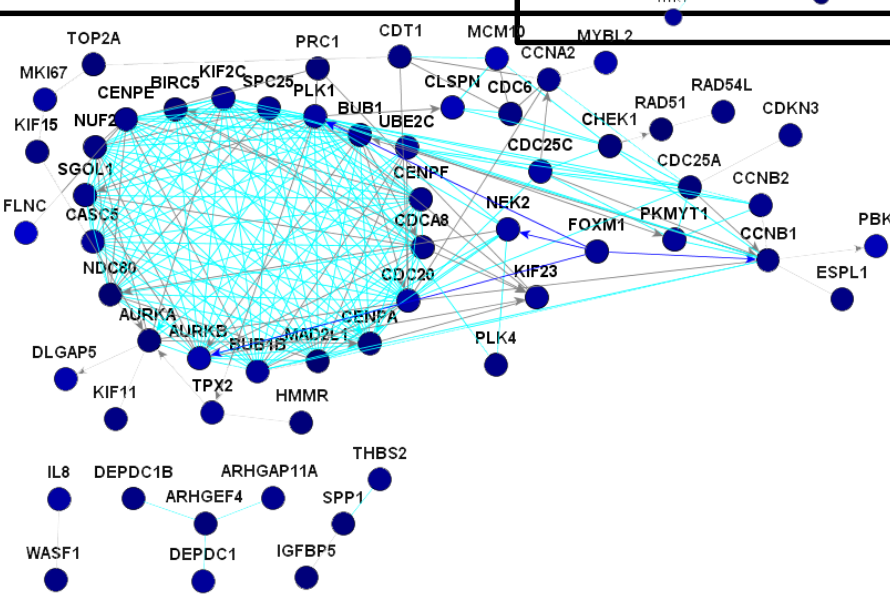

## miR-30e -- LUAD

## miR-30e -- KIRC

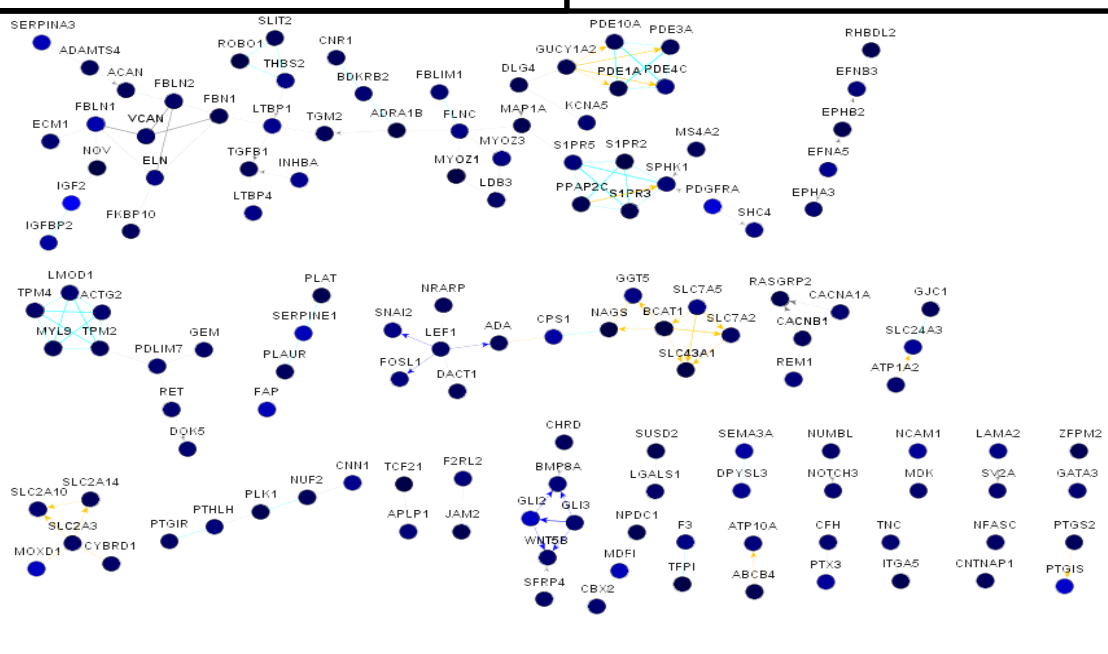

Supplement: S25 Fig — (PDF) [file pone.0140072.s025.pdf]

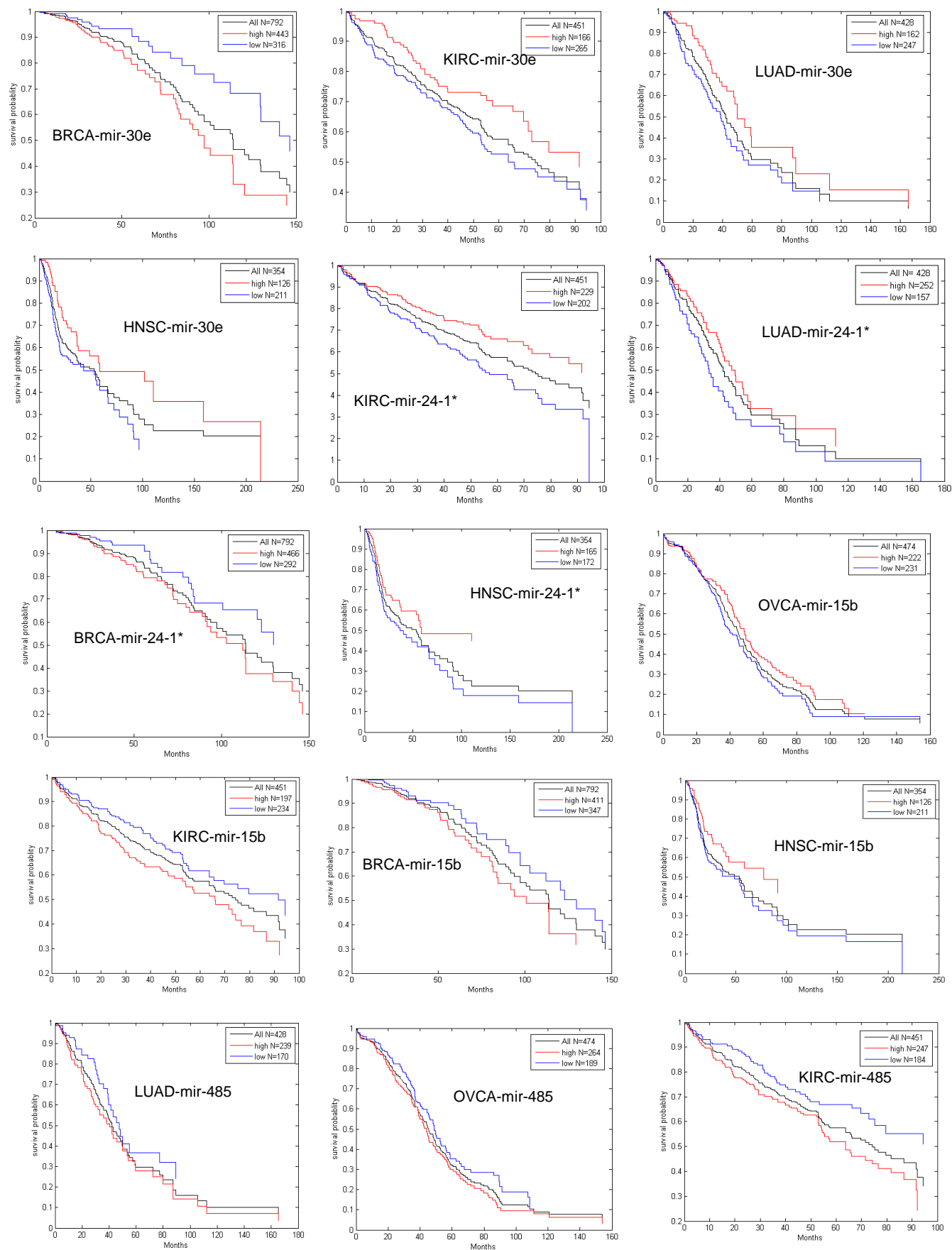

Supplement: S27 Fig — The curves for patients in the high–or low–miRNA expression groups, along with the overall survival curve for that population, are displayed. (PDF) [file pone.0140072.s027.pdf]

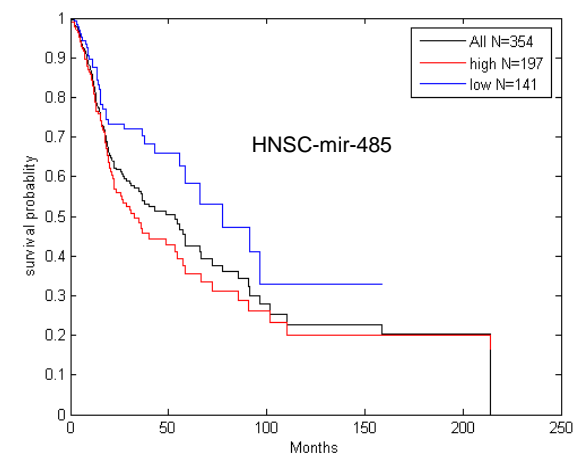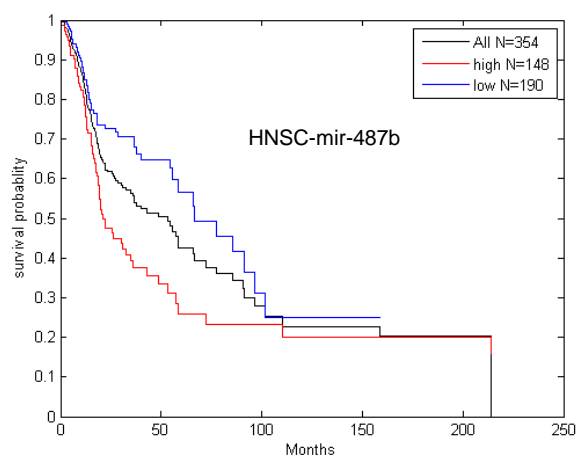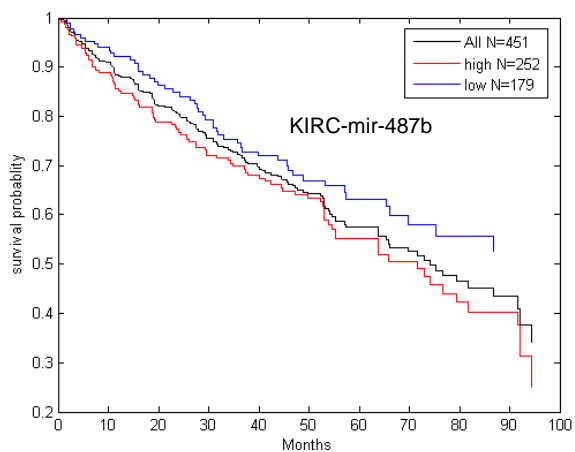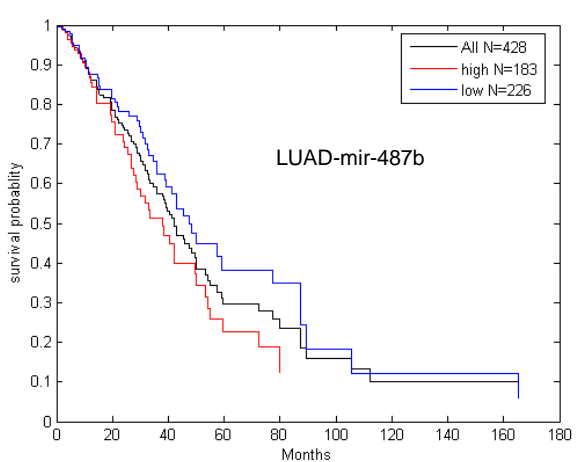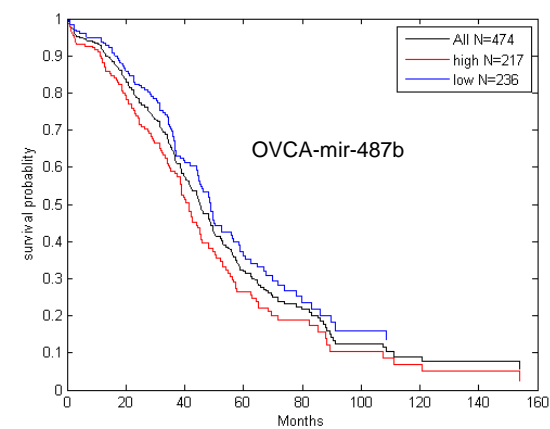

Supplement: S28 Fig — The curves for patients in the high–or low–miRNA expression groups, along with the overall survival curve for that population, are displayed. (PDF) [file pone.0140072.s028.pdf]

# Robust p-value

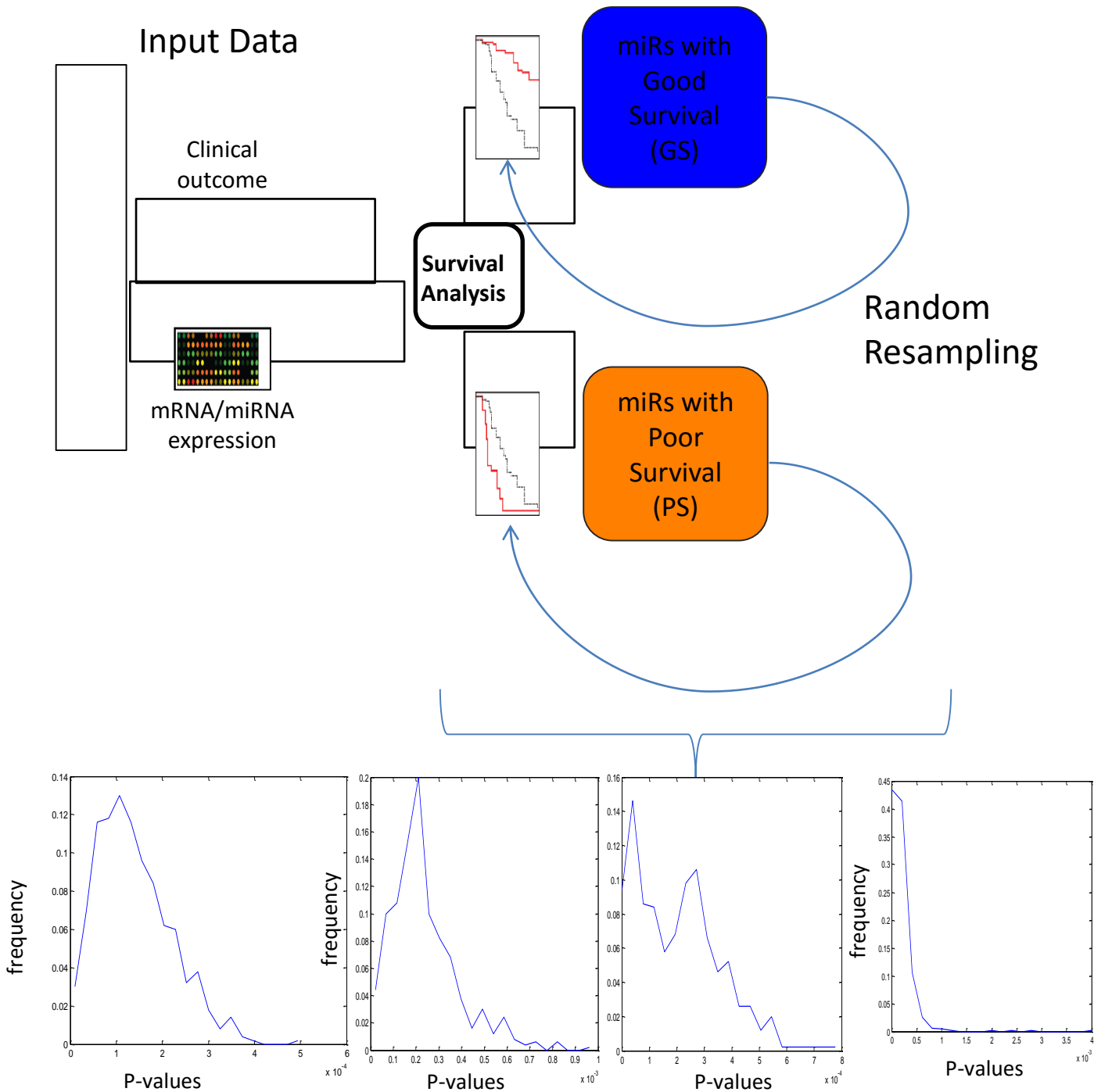

Supplement: S29 Fig — Some plots for the distribution of p-values are also displayed. (PDF) [file pone.0140072.s029.pdf]

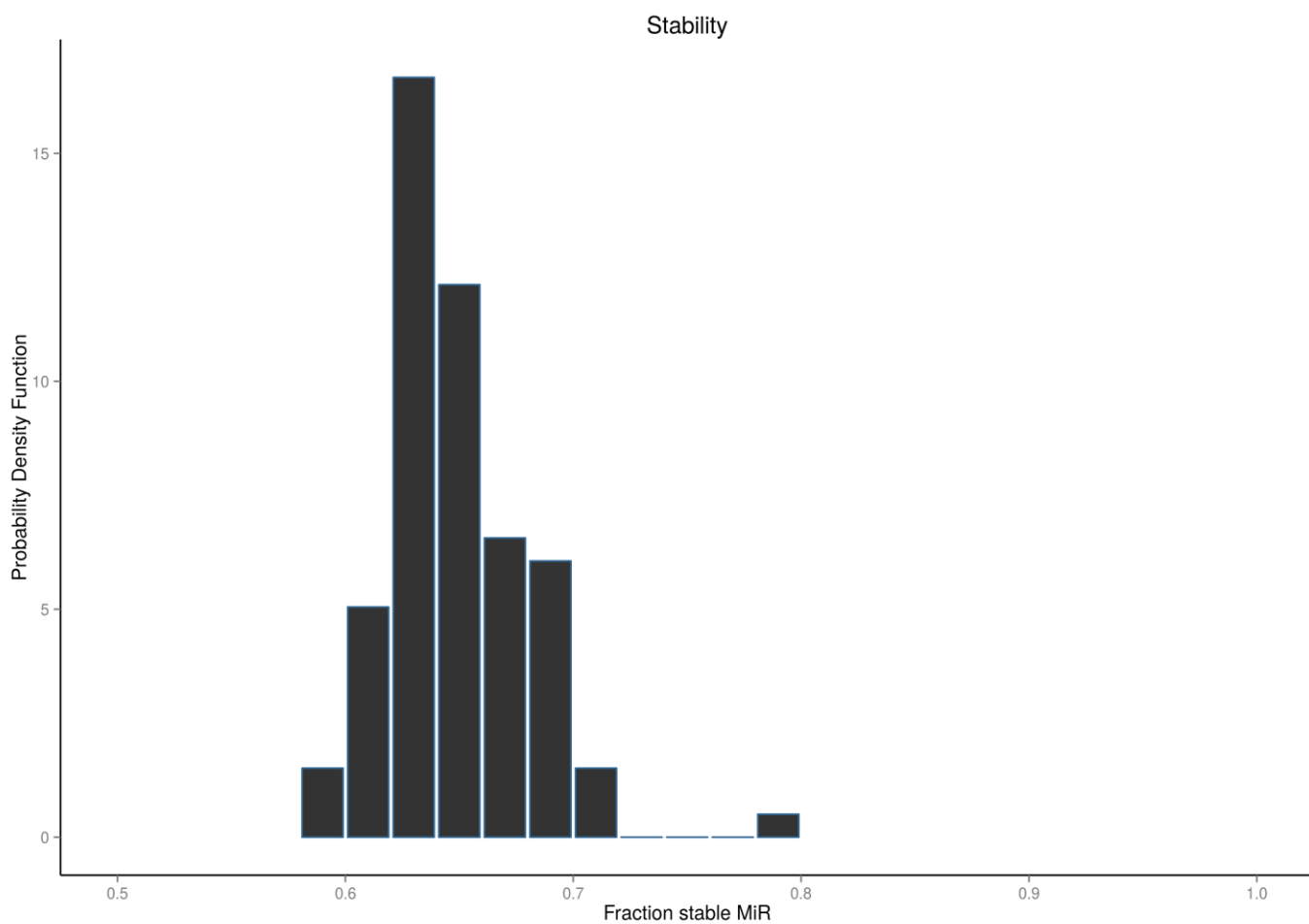

Supplement: S30 Fig — Starting with a kidney cancer dataset from TCGA, we created 100 simulated datasets by dropping 2% patients from the original dataset. On each simulated dataset, we then used the methodology of Reference [26] and create a list of miRNA with p-value smaller than 0.01. In this way we obtain 100 lists. We then enumerate miRNA which occur in 99 or more of these 100 lists; we will refer to this list of miRNA as stable miRNA. The displayed PDF is obtained by computing what fractions of the miRNA selected on each simulated dataset are stable. (PDF) [file pone.0140072.s030.pdf]

Figure A

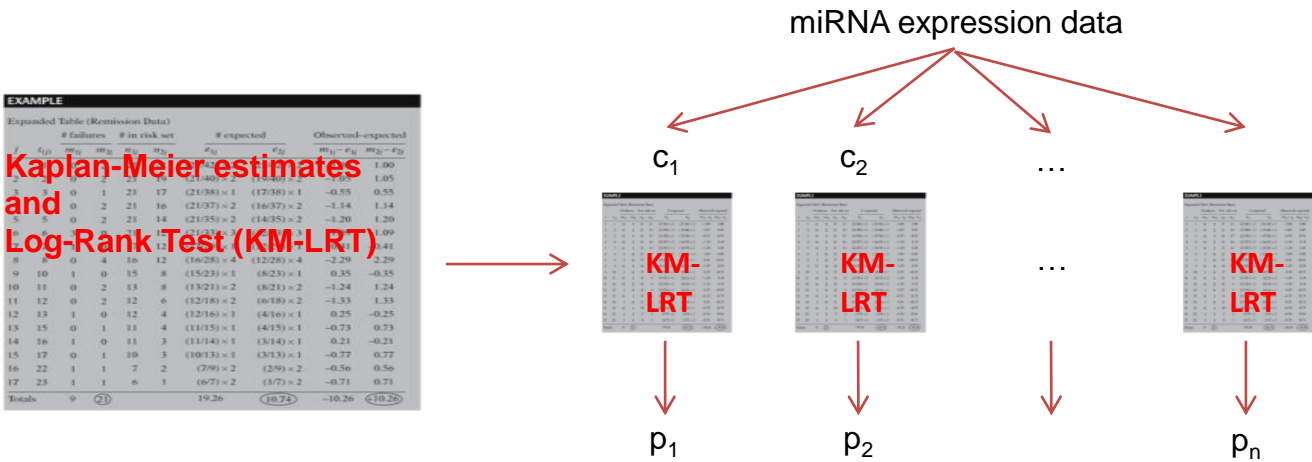

Figure B

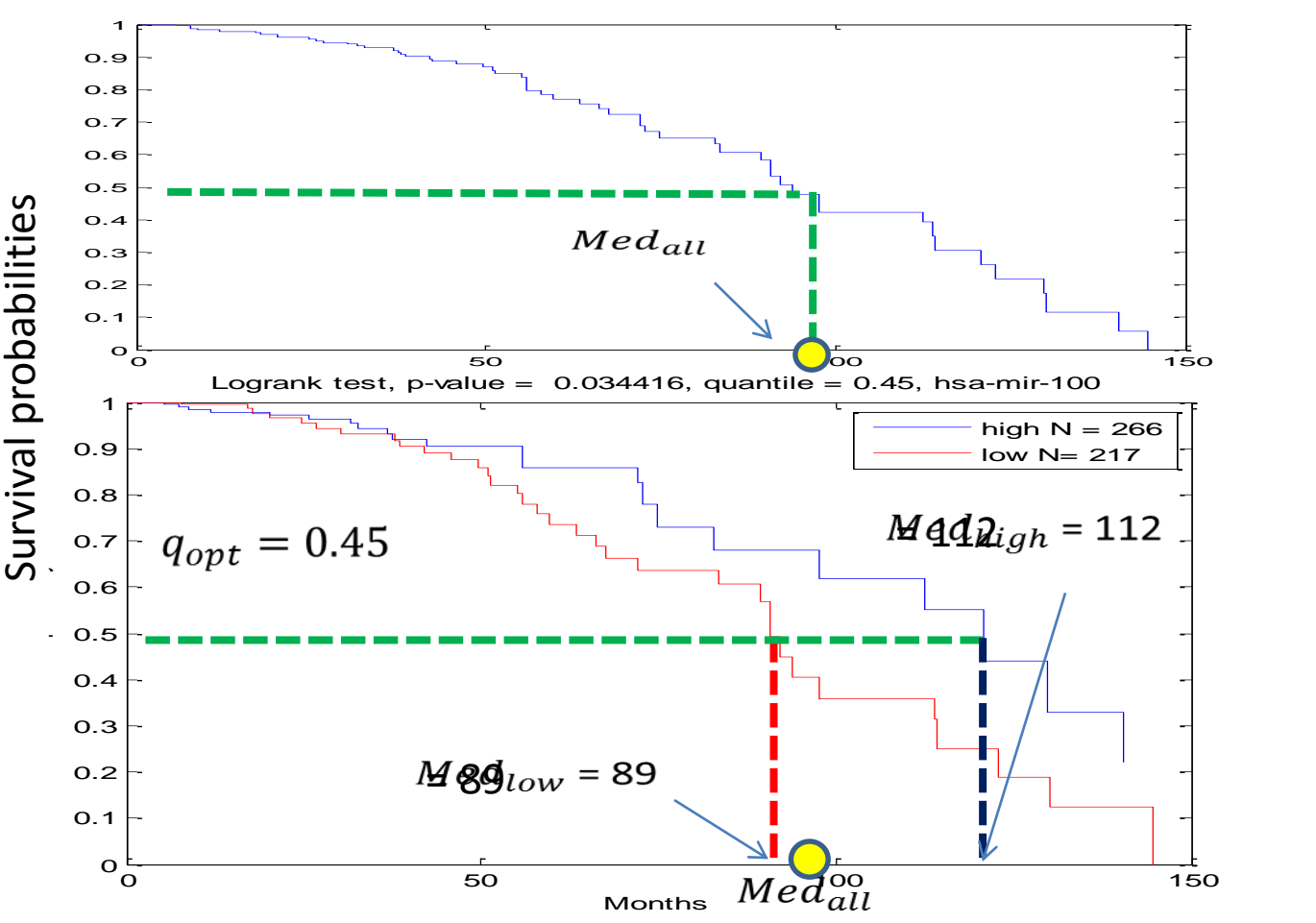

Supplement: S1 File — (Figure A) Schematic of our methodology, which involved computing Kaplan-Meier estimates and performing log-rank tests at different miRNA expression cut-offs. (Figure B) Schematic of our RSA. (PDF) [file pone.0140072.s031.pdf]
