# Supplementary material for: Robust Selection Algorithm (RSA) for Multi-Omic Biomarker Discovery; Integration with Functional Network Analysis to Identify miRNA Regulated Pathways in Multiple Cancers
Source: PLoS One. 2015 Oct 27;10(10):e0140072. doi: 10.1371/journal.pone.0140072 (PMC4623517; doi:10.1371/journal.pone.0140072)
Supplement: S8 Fig — (PDF) [file pone.0140072.s008.pdf]

List of genes associated to extracellular matrix

A2M	CIITA	COL6A2	FCN1	MBL2	PLOD3
ABI3BP	COL10A1	COL6A3	FCN2	MFAP4	PRELP
ACHE	COL11A1	COL6A6	FCN3	MMP1	PRSS2
ADAM15	COL11A2	COL7A1	FHOD1	MMP10	PRTN3
ADAM9	COL12A1	COL8A1	FMOD	MMP11	PTGES
ADAMTS2	COL13A1	COL8A2	FN1	MMP13	RELL2
ADIPOQ	COL14A1	COL9A1	GP6	MMP16	RPSA
ADORA2B	COL16A1	COL9A2	HDAC2	MMP19	SERPINH1
AMELX	COL17A1	COL9A3	HSD17B12	MMP2	SFRP2
AMN	COL18A1	COLEC12	IBSP	MMP26	SFTPA1
ANTXR1	COL1A1	COLQ	ID1	MMP3	SFTPA2
ANTXR2	COL1A2	COMP	KLK6	MMP7	SFTPD
ANXA2	COL20A1	CST3	LACRT	MMP8	SOD3
ARG1	COL23A1	CTGF	LAMA4	MMP9	SPARC
ATP7A	COL24A1	CTSL1	LAMB1	MRC2	SRGN
BGN	COL25A1	DCN	LAMB2	MSR1	THBS1
BMP4	COL29A1	DDR1	LAMC1	NF1	TLL1
C1QA	COL2A1	DDR2	LAMC2	NID2	TNXB
C1QB	COL3A1	DPP4	LAMC3	NOMO1	TRAM2
C1QC	COL4A1	DPT	LEPRE1	NOMO3	UCN
C1QL3	COL4A2	ECM2	LEPREL1	OPTC	USH2A
C1QTNF1	COL4A3	EDA	LEPREL2	P4HA1	VWF
C1QTNF5	COL4A3BP	ELANE	LMX1B	P4HA2	WDR33
C6orf15	COL4A4	EMID1	LOX	P4HA3	WNT4
CBLN1	COL4A5	EMID2	LOXL1	P4HB	XPNPEP2
CBLN3	COL4A6	EMILIN1	LOXL2	PCOLCE	ZBTB7B
CBLN4	COL5A1	EMILIN2	LOXL3	PEPD	
CD36	COL5A2	EP300	LOXL4	PITX2	
CD44	COL5A3	F2	LUM	PLOD1	
CEBPB	COL6A1	F2R	MARCO	PLOD2	

S8 Fig
