# Supplementary material for: Robust Selection Algorithm (RSA) for Multi-Omic Biomarker Discovery; Integration with Functional Network Analysis to Identify miRNA Regulated Pathways in Multiple Cancers
Source: PLoS One. 2015 Oct 27;10(10):e0140072. doi: 10.1371/journal.pone.0140072 (PMC4623517; doi:10.1371/journal.pone.0140072)
Supplement: S1 Table — Level 3 data was used for miRNA expression. For each cancer type, data can be found on the at the link using the platform type and last modified date mentioned in the table. (PDF) [file pone.0140072.s032.pdf]

Cancer Type	Platform	Date Last Modified
Breast	GA	April, 2013
	HiSeq	July, 2013
	Clinical	September, 2013
Ovarian	GA	NA
	HiSeq	April, 2013
	Clinical	September, 2013
Kidney	GA	April, 2013
	HiSeq	April, 2013
	Clinical	September, 2013
Lung	GA	April, 2013
	HiSeq	July, 2013
	Clinical	September, 2013
Head and Neck	GA	August, 2013
	HiSeq	April, 2013
	Clinical	September, 2013

S1 Table
