# Supplementary material for: Robust Selection Algorithm (RSA) for Multi-Omic Biomarker Discovery; Integration with Functional Network Analysis to Identify miRNA Regulated Pathways in Multiple Cancers
Source: PLoS One. 2015 Oct 27;10(10):e0140072. doi: 10.1371/journal.pone.0140072 (PMC4623517; doi:10.1371/journal.pone.0140072)
Supplement: S10 Fig — (PDF) [file pone.0140072.s010.pdf]

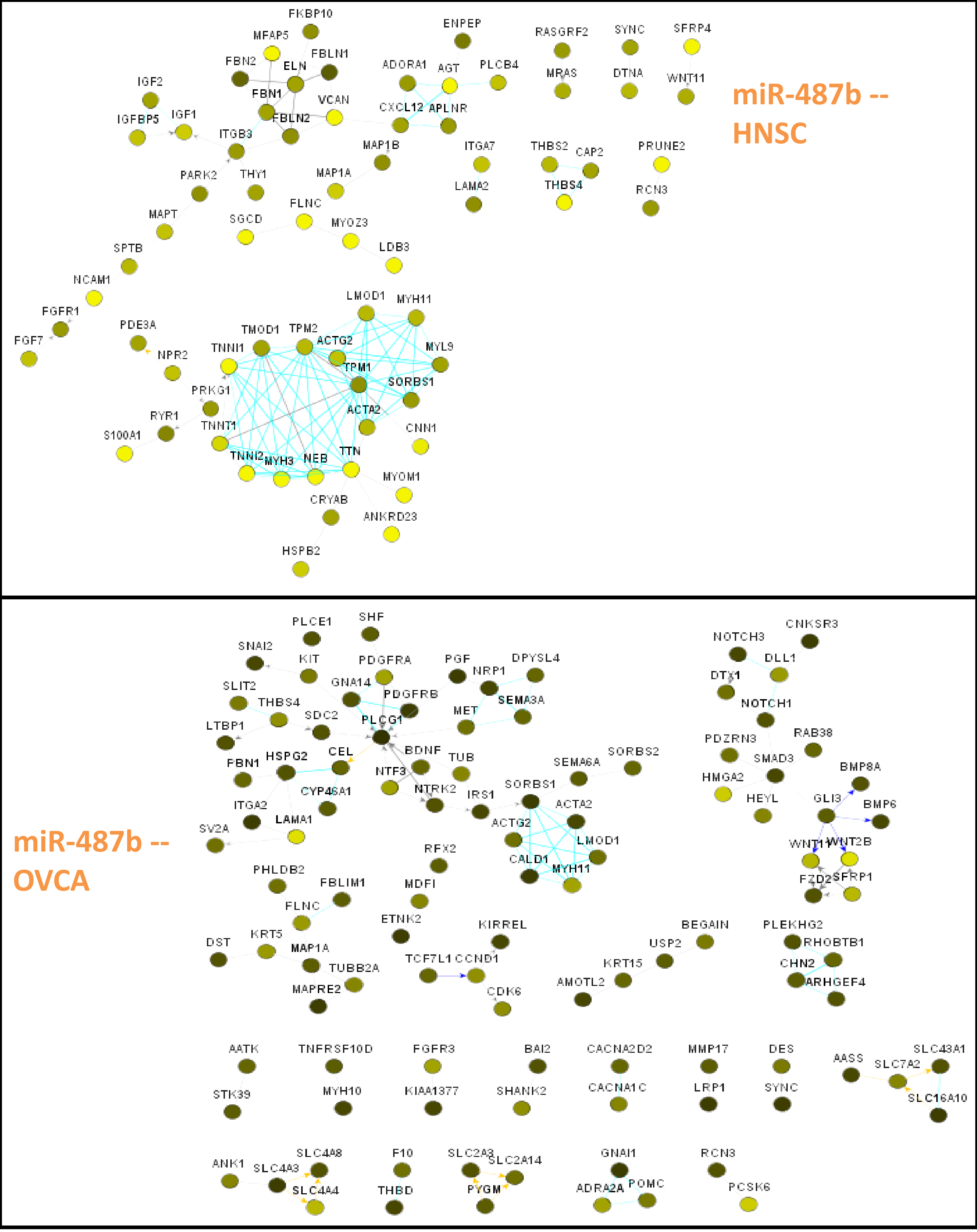
miR-487b -- HNSC
FKBP10
MFAP5
ENPEP
RASGRF2
SYNC
SFRP4
FBN2
ELN
FBLN1
ADORA1
AGT
PLCB4
MRAS
DTNA
WNT11
IGF2
FBN1
VCAN
CXCL12
APLNR
IGFBP5
IGF1
FBLN2
ITGB3
MAP1B
ITGA7
THBS2
CAP2
PRUNE2
PARK2
THY1
MAP1A
LAMA2
THBS4
RCN3
MAPT
SGCD
FLNC
MYOZ3
LDB3
SPTB
NCAM1
LMOD1
MYH11
FGFR1
PDE3A
TMOD1
TPM2
ACTG2
FGF7
NPR2
TNNI1
MYL9
TPM1
SORBS1
PRKG1
ACTA2
CNN1
RYR1
S100A1
TNNT1
TNNI2
MYH3
NEB
TTN
MYOM1
CRYAB
ANKRD23
HSPB2
miR-487b -- OVCA
PLCE1
SHF
SNAI2
KIT
PDGFRA
PGF
NRP1
DPYSL4
GNA14
SLIT2
PDGFRB
SEMA3A
THBS4
SDC2
MET
LTBP1
PLCG1
CEL
BDNF
TUB
FBN1
HSPG2
NTF3
CYP4A11
NTRK2
IRS1
ITGA2
LAMA1
SV2A
SEMA6A
SORBS2
SORBS1
ACTA2
ACTG2
LMOD1
CALD1
MYH11
RFX2
PHLDB2
FBLIM1
MDFI
FLNC
ETNK2
KIRREL
DST
KRT5
MAP1A
TUBB2A
MAPRE2
TCF7L1
CCND1
CDK6
NOTCH3
CNKSR3
DLL1
DTX1
NOTCH1
PDZRN3
RAB38
SMAD3
HMGA2
BMP8A
HEYL
GLI3
BMP6
WNT11
WNT2B
FZD2
SFRP1
BEGAIN
USP2
KRT15
AMOTL2
PLEKHG2
RHOBTB1
CHN2
ARHGEF4
AATK
TNFRSF10D
FGFR3
BAI2
CACNA2D2
MMP17
DES
AASS
SLC7A2
SLC43A1
SLC16A10
STK39
MYH10
KIAA1377
SHANK2
CACNA1C
LRP1
SYNC
ANK1
SLC4A3
SLC4A8
SLC4A4
F10
THBD
SLC2A3
SLC2A14
PYGM
GNAI1
ADRA2A
POMC
RCN3
PCSK6

S10 Fig
