# Supplementary material for: Robust Selection Algorithm (RSA) for Multi-Omic Biomarker Discovery; Integration with Functional Network Analysis to Identify miRNA Regulated Pathways in Multiple Cancers
Source: PLoS One. 2015 Oct 27;10(10):e0140072. doi: 10.1371/journal.pone.0140072 (PMC4623517; doi:10.1371/journal.pone.0140072)
Supplement: S17 Fig — (PDF) [file pone.0140072.s017.pdf]

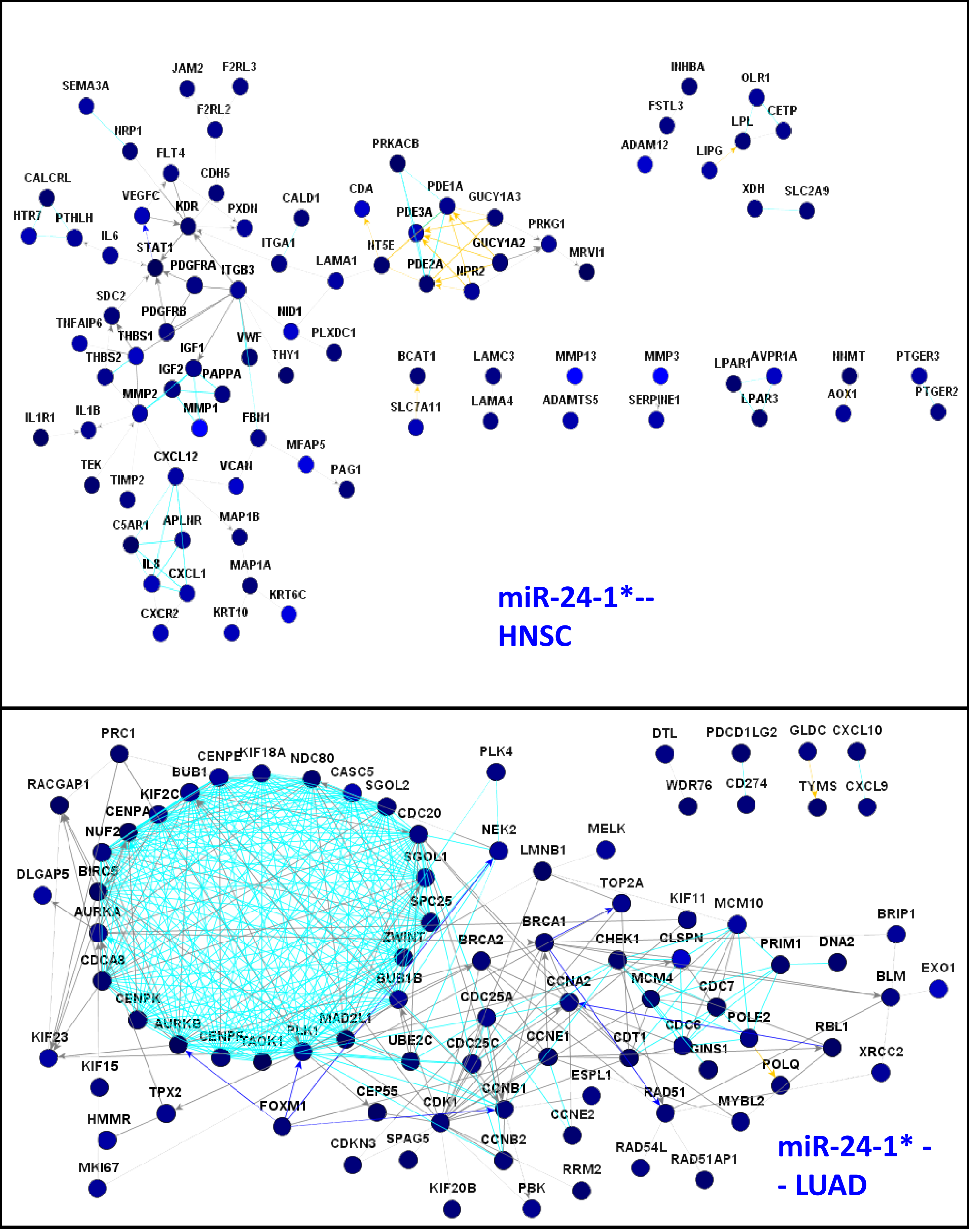
SEMA3A
JAM2
F2RL3
F2RL2
NRP1
FLT4
CDH5
CALCRL
VEGFC
KDR
PXDN
HTR7
PTHLH
IL6
STAT1
PDGFRA
ITGB3
ITGA1
CALD1
SDC2
PDGFRB
TNFAIP6
THBS1
THBS2
IGF1
IGF2
PAPPA
MMP2
MMP1
IL1R1
IL1B
TEK
TIMP2
CXCL12
C5AR1
APLNR
IL8
CXCL1
CXCR2
KRT10
VWF
THY1
NID1
PLXDC1
FBN1
MFAP5
PAG1
VCAN
MAP1B
MAP1A
KRT6C
LAMA1
PRKACB
CDA
PDE1A
GUCY1A3
PDE3A
NT5E
GUCY1A2
PRKG1
PDE2A
NPR2
MRVI1
INHBA
OLR1
FSTL3
CETP
LPL
ADAM12
LIPG
XDH
SLC2A9
BCAT1
SLC7A11
LAMC3
LAMA4
MMP13
ADAMTS5
MMP3
SERPINE1
LPAR1
AVPR1A
LPAR3
NNMT
AOX1
PTGER3
PTGER2
miR-24-1*-- HNSC
PRC1
RACGAP1
CENPE
KIF18A
NDC80
BUB1
CASC5
SGOL2
KIF2C
CENPA
CDC20
NUF2
PLK4
NEK2
MELK
LMNB1
SGOL1
DLGAP5
BIRC5
AURKA
SPC25
TOP2A
ZWINT
BRCA1
KIF11
MCM10
BRIP1
CDCA8
BRCA2
CHEK1
CLSPN
PRIM1
DNA2
BUB1B
CCNA2
MCM4
CDC7
BLM
EXO1
CENPK
CDC25A
CDC6
POLE2
RBL1
AURKB
MAD2L1
CENPF
TAOK1
PLK1
UBE2C
CDC25C
CCNE1
CDT1
GINS1
XRCC2
KIF23
POLQ
KIF15
ESPL1
TPX2
CEP55
CCNB1
RAD51
MYBL2
HMMR
FOXM1
CDK1
CCNE2
CDKN3
SPAG5
CCNB2
RAD54L
RAD51AP1
MKI67
RRM2
KIF20B
PBK
DTL
PDCD1LG2
GLDC
CXCL10
WDR76
CD274
TYMS
CXCL9
miR-24-1* -- LUAD

S17 Fig
