# Supplementary material for: Robust Selection Algorithm (RSA) for Multi-Omic Biomarker Discovery; Integration with Functional Network Analysis to Identify miRNA Regulated Pathways in Multiple Cancers
Source: PLoS One. 2015 Oct 27;10(10):e0140072. doi: 10.1371/journal.pone.0140072 (PMC4623517; doi:10.1371/journal.pone.0140072)
Supplement: S19 Fig — (PDF) [file pone.0140072.s019.pdf]

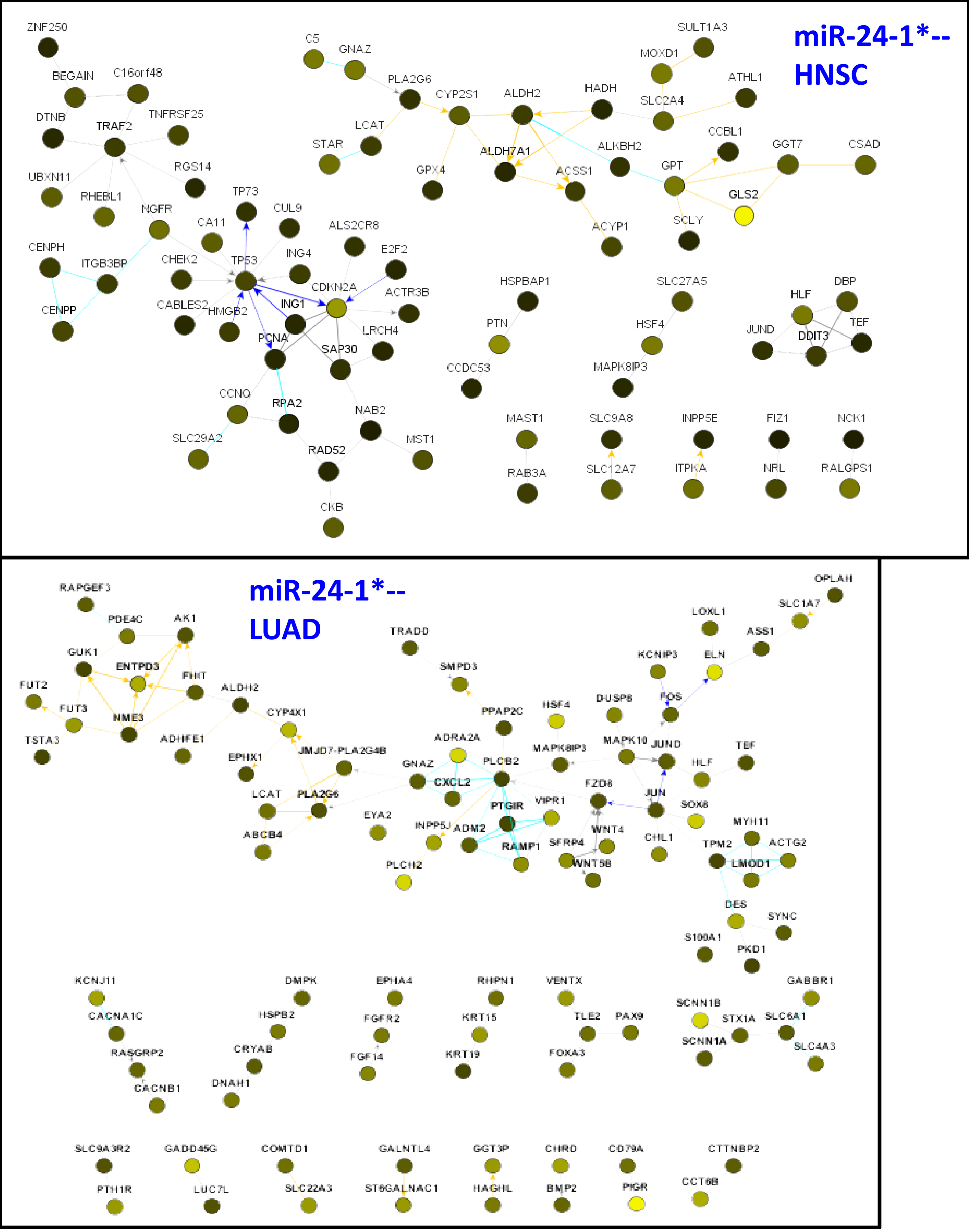
miR-24-1*--HNSC
ZNF250
BEGAIN
C16orf48
DTNB
TRAF2
TNFRSF25
RGS14
UBXN11
RHEBL1
NGFR
TP73
CUL9
CA11
CENPH
ITGB3BP
CHEK2
TP53
ING4
CENPP
CABLES2
HMGB2
ING1
CDKN2A
PCNA
SAP30
LRCH4
ACTR3B
ALS2CR8
E2F2
CCNO
RPA2
NAB2
SLC29A2
RAD52
MST1
CKB
C5
GNAZ
PLA2G6
CYP2S1
LCAT
STAR
GPX4
ALDH2
HADH
ALDH7A1
ACSS1
ALKBH2
ACYP1
SULT1A3
MOXD1
SLC2A4
ATHL1
CCBL1
GGT7
CSAD
GPT
GLS2
SCLY
HSPBAP1
PTN
CCDC53
SLC27A5
HSF4
MAPK8IP3
HLF
DBP
JUND
DDIT3
TEF
MAST1
RAB3A
SLC9A8
SLC12A7
INPP5E
ITPKA
FIZ1
NRL
NCK1
RALGPS1
miR-24-1*--LUAD
RAPGEF3
PDE4C
AK1
GUK1
ENTPD3
FHIT
FUT2
FUT3
NME3
TSTA3
ALDH2
ADHFE1
CYP4X1
EPHX1
JMJD7-PLA2G4B
LCAT
PLA2G6
ABCB4
TRADD
SMPD3
PPAP2C
ADRA2A
GNAZ
CXCL2
PLCB2
EYA2
INPP5J
ADM2
PTGIR
VIPR1
RAMP1
PLCH2
HSF4
MAPK8IP3
DUSP8
MAPK10
FOS
JUND
FZD8
JUN
WNT4
SFRP4
WNT5B
CHL1
SOX8
KCNIP3
ELN
LOXL1
ASS1
SLC1A7
OPLAH
TEF
HLF
MYH11
TPM2
ACTG2
LMOD1
DES
SYNC
S100A1
PKD1
KCNJ11
CACNA1C
RASGRP2
CACNB1
DMPK
HSPB2
CRYAB
DNAH1
EPHA4
FGFR2
FGF14
RHPN1
KRT15
KRT19
VENTX
TLE2
PAX9
FOXA3
SCNN1B
STX1A
SCNN1A
SLC6A1
GABBR1
SLC4A3
SLC9A3R2
PTH1R
GADD45G
LUC7L
COMTD1
SLC22A3
GALNTL4
ST6GALNAC1
GGT3P
HAGHL
CHRD
BMP2
CD79A
PIGR
CTTNBP2
CCT6B

S19 Fig
