# Supplementary material for: Robust Selection Algorithm (RSA) for Multi-Omic Biomarker Discovery; Integration with Functional Network Analysis to Identify miRNA Regulated Pathways in Multiple Cancers
Source: PLoS One. 2015 Oct 27;10(10):e0140072. doi: 10.1371/journal.pone.0140072 (PMC4623517; doi:10.1371/journal.pone.0140072)
Supplement: S22 Fig — (PDF) [file pone.0140072.s022.pdf]

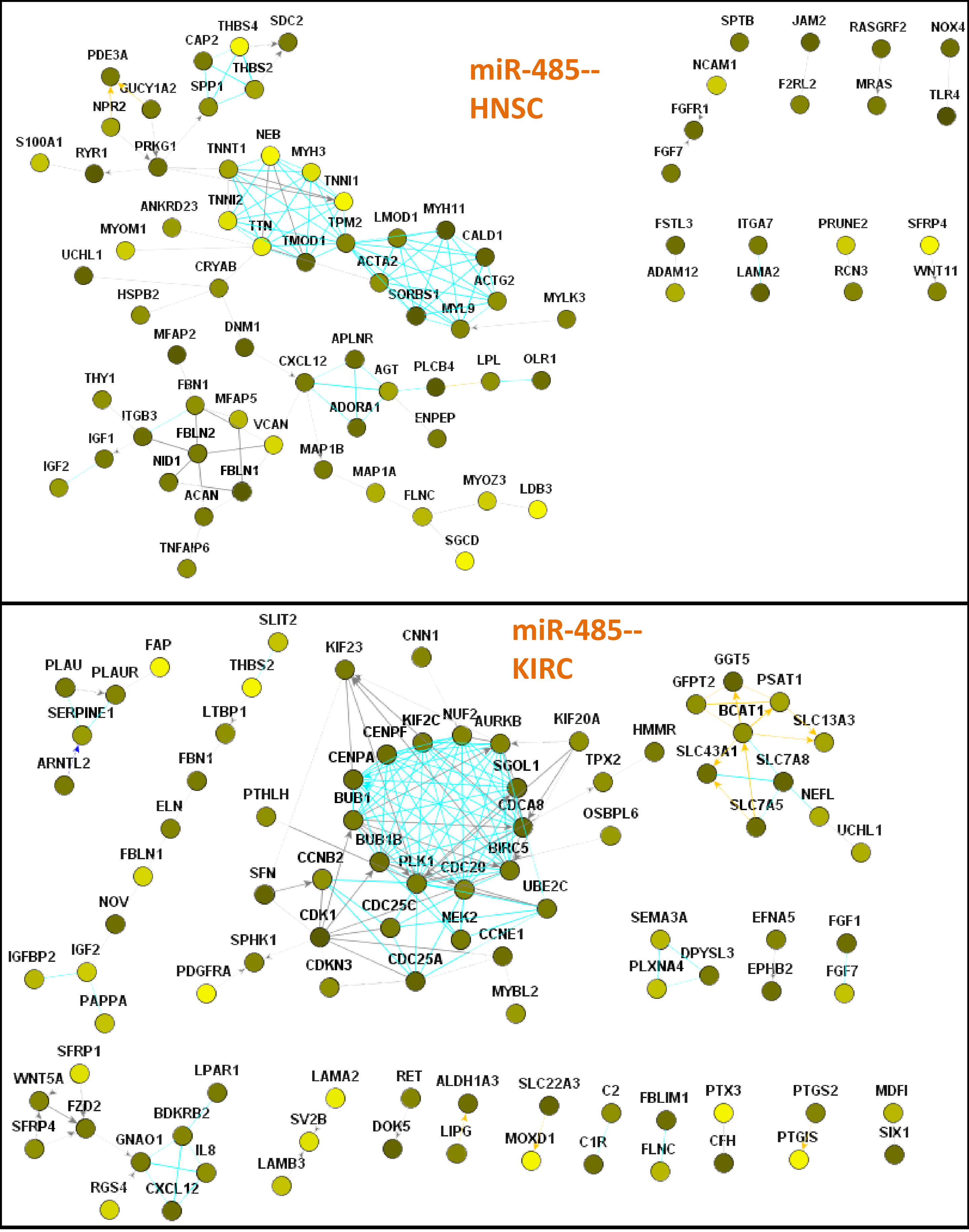
miR-485--HNSC
THBS4
SDC2
CAP2
PDE3A
THBS2
GUCY1A2
SPP1
NPR2
S100A1
RYR1
PRKG1
TNNT1
NEB
MYH3
TNNI1
ANKRD23
TNNI2
MYOM1
TTN
TPM2
LMOD1
MYH11
UCHL1
TMOD1
CALD1
CRYAB
ACTA2
ACTG2
HSPB2
SORBS1
MYL9
MYLK3
MFAP2
DNM1
APLNR
CXCL12
AGT
PLCB4
LPL
OLR1
THY1
FBN1
MFAP5
ITGB3
VCAN
ADORA1
ENPEP
IGF1
FBLN2
MAP1B
IGF2
NID1
FBLN1
MAP1A
ACAN
FLNC
MYOZ3
LDB3
TNFAIP6
SGCD
SPTB
JAM2
RASGRF2
NOX4
NCAM1
F2RL2
MRAS
TLR4
FGFR1
FGF7
FSTL3
ITGA7
PRUNE2
SFRP4
ADAM12
LAMA2
RCN3
WNT11
miR-485--KIRC
SLIT2
CNN1
FAP
KIF23
PLAU
PLAUR
THBS2
GGT5
GFPT2
PSAT1
SERPINE1
LTBP1
BCAT1
KIF2C
NUF2
AURKB
KIF20A
CENPF
HMMR
SLC13A3
ARNTL2
SLC43A1
CENPA
SGOL1
TPX2
SLC7A8
FBN1
PTHLH
BUB1
CDCA8
NEFL
ELN
OSBPL6
SLC7A5
UCHL1
BUB1B
BIRC5
FBLN1
CCNB2
PLK1
CDC20
SFN
UBE2C
NOV
CDC25C
CDK1
NEK2
CCNE1
SEMA3A
EFNA5
FGF1
SPHK1
DPYSL3
IGFBP2
IGF2
CDKN3
CDC25A
PLXNA4
EPHB2
FGF7
PDGFRA
MYBL2
PAPPA
SFRP1
LPAR1
WNT5A
LAMA2
RET
ALDH1A3
SLC22A3
C2
FBLIM1
PTX3
PTGS2
MDFI
FZD2
BDKRB2
SV2B
SFRP4
DOK5
LIPG
MOXD1
C1R
FLNC
CFH
PTGIS
SIX1
GNAO1
IL8
LAMB3
RGS4
CXCL12

S22 Fig
