# Supplementary material for: Robust Selection Algorithm (RSA) for Multi-Omic Biomarker Discovery; Integration with Functional Network Analysis to Identify miRNA Regulated Pathways in Multiple Cancers
Source: PLoS One. 2015 Oct 27;10(10):e0140072. doi: 10.1371/journal.pone.0140072 (PMC4623517; doi:10.1371/journal.pone.0140072)
Supplement: S26 Fig — (PDF) [file pone.0140072.s026.pdf]

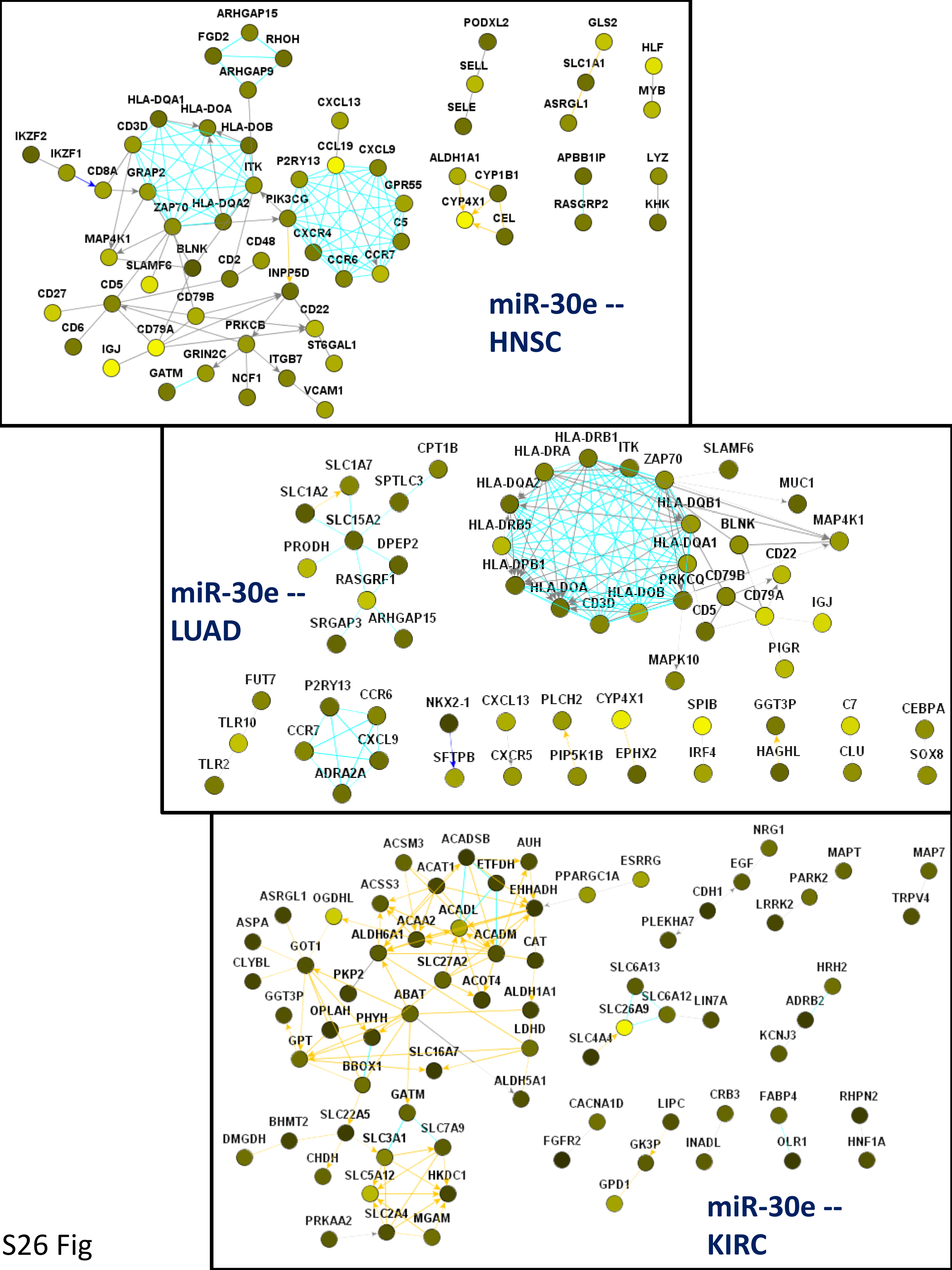
miR-30e -- HNSC
ARHGAP15
FGD2
RHOH
ARHGAP9
PODXL2
SELL
SELE
GLS2
SLC1A1
ASRGL1
HLF
MYB
HLA-DQA1
HLA-DOA
HLA-DOB
CD3D
IKZF2
IKZF1
CD8A
GRAP2
ITK
CXCL13
CCL19
CXCL9
P2RY13
GPR55
PIK3CG
C5
ZAP70
HLA-DQA2
CXCR4
CCR6
CCR7
MAP4K1
BLNK
CD48
SLAMF6
CD2
INPP5D
CD27
CD5
CD79B
CD22
CD6
CD79A
PRKCB
ST6GAL1
IGJ
GRIN2C
ITGB7
GATM
NCF1
VCAM1
ALDH1A1
CYP1B1
CYP4X1
CEL
APBB1IP
RASGRP2
LYZ
KHK
miR-30e -- LUAD
CPT1B
SLC1A7
SPTLC3
SLC1A2
SLC15A2
PRODH
DPEP2
RASGRF1
SRGAP3
ARHGAP15
HLA-DRB1
HLA-DRA
ITK
ZAP70
SLAMF6
HLA-DQA2
MUC1
HLA-DQB1
HLA-DRB5
BLNK
MAP4K1
HLA-DQA1
CD22
HLA-DPB1
PRKCQ
CD79B
HLA-DOA
HLA-DOB
CD3D
CD79A
IGJ
CD5
PIGR
MAPK10
FUT7
TLR10
TLR2
P2RY13
CCR6
CCR7
CXCL9
ADRA2A
NKX2-1
SFTPB
CXCL13
CXCR5
PLCH2
PIP5K1B
CYP4X1
EPHX2
SPIB
IRF4
GGT3P
HAGHL
C7
CLU
CEBPA
SOX8
miR-30e -- KIRC
ACSM3
ACADSB
AUH
ACAT1
ETFDH
ESRRG
PPARGC1A
EHHADH
ACSS3
ASRGL1
OGDHL
ACADL
ACAA2
ASPA
ACADM
ALDH6A1
CAT
GOT1
CLYBL
SLC27A2
PKP2
ACOT4
GGT3P
ABAT
ALDH1A1
OPLAH
PHYH
LDHD
GPT
SLC16A7
BBOX1
ALDH5A1
GATM
BHMT2
SLC22A5
SLC7A9
DMGDH
SLC3A1
CHDH
SLC5A12
HKDC1
SLC2A4
MGAM
PRKAA2
NRG1
EGF
MAPT
MAP7
CDH1
PARK2
TRPV4
LRRK2
PLEKHA7
SLC6A13
SLC6A12
LIN7A
SLC26A9
SLC4A4
HRH2
ADRB2
KCNJ3
CACNA1D
FGFR2
LIPC
GK3P
GPD1
CRB3
INADL
FABP4
OLR1
RHPN2
HNF1A

S26 Fig
